# Occurrence and Impact of Electric-Field-Induced Discontinuities in Correlation Energies from Localized Pair-Natural-Orbital Methods

**DOI:** 10.1021/acs.jpca.5c05210

**Published:** 2025-10-16

**Authors:** Jose P. Madriaga, T. Daniel Crawford

**Affiliations:** Department of Chemistry, Virginia Tech, Blacksburg, Virginia 24061, United States

## Abstract

We report an investigation of discontinuities in the correlation energy produced by external static electric fields within the local pair-natural-orbital coupled-cluster singles and doubles (LPNO-CCSD) method. Such discontinuities arise as a result of variations in both the dimensions and character of the pairwise virtual-orbital domains resulting from changes in the strength of the field. Using several small-molecule test cases – water, fluoroethylene, hypofluorous acid and *cis*-1,3-butadiene we observe that, although the discontinuities in the correlation energy are small (typically 1 μ*E*_*h*_), they can yield substantial errors in higher-order electric-field-dependent properties computed using finite-difference techniques. For the static hyperpolarizability (third derivative of the energy with respect to the field) of water, for example, the discrepancies between LPNO-CCSD and canonical-MO CCSD methods can exceed 100%. Furthermore, weak-field displacements that should normally decrease errors in numerical differentiation can yield orders-of-magnitude errors due to magnification of the energy discontinuities by small field-displacement denominators. For larger molecules, such fields can produce dramatic errors in the static polarizability (second derivative of the energy with respect to the field) and hyperpolarizability even with very tight PNO cutoffs. The use of basis sets containing diffuse functions, which are essential for reliable predictions of field-dependent response properties, tend to exacerbate the observed errors. In addition, the use of fixed virtual PNO dimensions does no resolve the problem due to mixing of the PNOs relative to zero-field orbitals as a result of large condition numbers of the pair-correlation densities.

## Graphical abstract

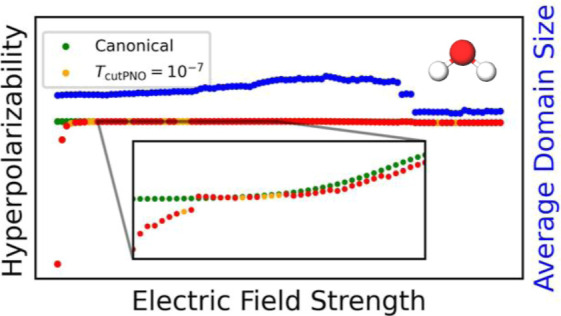

## 1 Introduction

Great progress has been reported over the last 15 years in the use of pair natural orbitals (PNOs)^1–18^ to reduce the polynomial scaling of wave function-based quantum chemical methods, especially for coupled cluster (CC) theory.^19–21^ In the PNO approach, compact virtual molecular orbital (MO) subspaces are constructed for each unique pair of localized occupied MOs using an approximate correlated one-electron density (conventionally second-order many-body perturbation-theory). As well demonstrated by Neese and co-workers,^22,23^ the LPNO-CC method and its domain-based (DLPNO-CC) counterpart^24–26^ produce highly predictable sparsity in the resulting ground-state CC wave function and have been shown to provide excellent recovery of correlation energies and related properties.^27,28^

In a recent effort to identify robust wave function-based alternatives to density-functional methods, Naim et al. reported^29^ benchmark computations of static-electric-field properties, including dipole moments, polarizabilities, and first- and second-hyperpolarizabilities for a large set of test molecules ranging from carbon monoxide to functionalized π-conjugated push–pull polymers. They explored a number of single-reference approaches, including second-order Møller–Plesset perturbation theory (MP2), coupled cluster singles and doubles (CCSD), CCSD including perturbative triples [CCSD­(T)], and found that all were capable of yielding accurate predictions of static nonlinear optical properties. However, citing the high-degree polynomial scaling of these methods, they also explored a wide range of reduced-scaling approaches, including local MP2 (LMP2), the localized natural orbital CC [LNO-CCSD and LNO-CCSD­(T)],^30^ DLPNO-CCSD, DLPNO-CCSD­(T), and others. They observed large numerical instabilities for these reduced-scaling methods, leading to sometimes dramatic errors (up to 100% in the worst cases) in the resulting higher-order properties. After careful exploration of the various convergence parameters and cutoffs required for such computations, Naim et al. concluded that these errors were the result of a lack of precision of the field-dependent, single-point energies needed for the numerical differentiation, and that these energies were highly dependent on the orbital localization cutoffs.

The goal of the present work is to provide a detailed examination of what may be the underlying source of these errors, viz. discontinuities in the correlation energy as a function of an external electric field, particularly for static hyperpolarizability (third derivative of the energy with respect to the field). Using four small molecular test cases, we find that, as the field varies in both magnitude and direction in order to obtain the single-point energies needed for the corresponding numerical derivatives, abrupt changes can occur in the sizes of the pairwise virtual natural-orbital domains. While these yield relatively small errors in the energies themselves, numerical differentiation across such discontinuities can produce large errors in the resulting properties, especially for higher-order derivatives. We examine the dependence of these discontinuities on a variety of factors, including PNO cutoffs, orbital relaxation effects, choice of basis set, etc. in order to estimate the magnitude of the errors and identify practical approaches to overcoming them. In addition, we examine an approach that may amerliorate these errors.

## 2 Theoretical Background

### 2.1 Local Pair Natural Orbital Coupled Cluster Theory

In CC theory, the correlated wave function makes use of an exponential *Ansatz*

1 $$|\Psi_{CC}\rangle =e^{\mathrm{T̂}}|0\rangle$$

where |0⟩ denotes the reference determinant built from the occupied MOs, typically the Hartree–Fock wave function. The cluster operator *T̂*, which contains the wave function amplitudes, generates substituted determinants from |0⟩ depending on a selected truncation level (e.g., the CCSD approach noted earlier includes only ${\mathrm{T̂}}_{1}$ and ${\mathrm{T̂}}_{2}$ ). Inserting eq 1 into the Schrödinger equation, multiplying by the inverse of the exponential, and projecting onto the reference determinant or the set of substituted determinants generated by *T̂* yields the CC energy and amplitude equations, viz.

2 $$E_{CCSD}=\langle 0|\mathrm{H̅}|0\rangle$$

and

$$\langle \phi_{ij...}^{ab...}|\mathrm{H̅}|0\rangle =0$$ 3

Here $\mathrm{H̅}=e^{-\mathrm{T̂}}\;Ĥ{\;e}^{\mathrm{T̂}}$ is the similarity-transformed Hamiltonian, and the indices *i*, *j*, ... (*a*, *b*, ...) denote occupied (virtual) MOs.

In the local pair natural orbital (LPNO) approach, the occupied MOs are first localized using standard methods such as that suggested by Pipek–Mezey^31^ or by Boys.^32^ Next, for each pair of occupied orbitals, *ij*, a compact set of virtual PNOs $\left(\{{\mathrm{a̅}}_{ij}\}\right)$ is obtained using a unitary transformation

$$\left|{\mathrm{a̅}}_{ij}\rangle =\underset{a}{\sum }Q_{\mathrm{a̅}a}^{ij}|a\right\rangle$$ 4

where the matrix $Q_{\mathrm{a̅}a}^{ij}$ is comprised of the eigenvectors of the pairwise virtual–virtual block of an approximate one-electron density matrix

5 $$D^{ij}Q^{ij}=n^{ij}Q^{ij}$$

In the most common LPNO-CC implementations, ***D*** ^*ij*^ is constructed from the doubles amplitudes, ***T*** ^*ij*^, from the first-order wave function of many-body perturbation theory (MBPT)

6 $$D^{ij}=\frac{2}{1+\delta_{ij}}\left(T^{ij}{\mathrm{T̃}}^{ij†}+T^{ij†}{\mathrm{T̃}}^{ij}\right)$$

where

7 $${\mathrm{T̃}}^{ij}=2T^{ij}-T^{ij†}$$

and the MOs are assumed to be spin-restricted. For the related PNO++ method,^33,34^ which is intended to obtain molecular response properties, the second-order (field) perturbed MBPT density is used. In eq 5, the eigenvalues, ***n*** ^*ij*^, are occupation numbers that are assumed to identify the most significant virtual PNOs for each pair, *ij*, for recovering the correlation energy. Thus, the size of the virtual space/correlation domain for the given pair is reduced by removing all PNOs with occupation numbers falling below a predetermined threshold, *T*_PNOcutoff_. The first-order wave function amplitudes, ***T*** ^*ij*^, are computed iteratively, with each update given by

8 $$\Delta T_{ab}^{ij}=\frac{R_{ab}^{ij}}{F_{ii}+F_{jj}-\epsilon_{a}^{ij}-\epsilon_{b}^{ij}}$$

where

9 $$R_{ab}^{ij}=P_{ab}^{ij}\left(\frac{1}{2}\left\langle ij|ab\right\rangle +T_{ae}^{ij}F_{be}-T_{ab}^{im}F_{mj}\right)$$

Here, the *F*_*ii*_ and *F*_*jj*_ are the diagonal elements of the Fock matrix corresponding to localized occupied orbitals, and ϵ_*a*_ and ϵ_*b*_ are virtual-MO energies, while the operator *P*_*ab*_^*ij*^ symmetrizes the residual, *R*_*ab*_^*ij*^, with respect to permutation between *ia* and *jb*. In addition, the virtual-PNO components of the energy denominator needed to update the wave function amplitudes [cf. eq 3] in each iteration are obtained by first transforming the virtual-MO block of the Fock matrix to the PNO space

10 $$F_{\mathrm{a̅}\mathrm{b̅}}^{ij}=\sum_{ab}Q_{\mathrm{a̅}a}^{ij}F_{ab}{\left(Q_{\mathrm{b̅}b}^{ij}\right)}^{†}$$

and then transforming the result to the semicanonical basis for the given pair

11 $$F^{ij}L^{ij}=\epsilon^{ij}L^{ij}$$

The addition of an external dipole field induces several responses within the PNO-CC wave function and associated energy. If the field is engaged at the beginning of the computation of the Hartree–Fock orbitals, the approximate (MP2) density, the PNOs, the CC wave function amplitudes, and finally the energy will change relative to those obtained in the absence of the field. This is commonly referred to as a “fully relaxed” calculation. If the field is switched on after the Hartree–Fock step, then the computation of the MP2 density and subsequent steps will still be affected because the field alters the Fock matrix, as shown in the denominator of eq 8, and thus the MO energies. This is referred to as a “PNO relaxed” calculation.^35^ In their work introducing DLPNO-CC calculations of first-order properties, Neese and co-workers found that the contribution of the latter is roughly an order of magnitude smaller than that of the full relaxation of the SCF MOs.^35^ If the field is added after the construction of the PNO space, then it only affects the calculation of the wave function amplitudes and CC energy (again due to its appearance in the Fock matrix), which is an “unrelaxed” calculation.

Since the field in the fully relaxed and PNO relaxed calculations can alter the magnitudes of the occupation numbers, ***n*** ^*ij*^, this introduces the possibility of discontinuities in the electric-field-dependent CC energy if the sizes of the pairwise virtual orbital domains change with the field strength. The central question of the present work is whether and by how much the domains change when the field strength changes (both in magnitude and direction), and how much the resulting energy discontinuities affect the numerical derivatives needed for static field-dependent properties, such as those reported by Naim et al.^29^ We note that further approximations such as domain-based LPNO-CCSD (which employs projected atomic orbitals to generate the virtual PNO sets), may alter the virtual orbital domains and introduce additional errors in the resulting properties.

## 3 Computational Details

We chose several molecular test cases for this work: water, hypofluorous acid, fluoroethylene and *cis*-1,3-butadiene because these exhibit strong dipole moments and corresponding higher-order field responses. The geometries for each molecule are reported in the Supporting Information. We carried out a series of computations to answer several questions related to the field-dependent-energy discontinuities described in the Section 2. First, do these discontinuities occur, and, if so, what affect do they have on the use of numerical differentiation to compute field-dependent response properties? Second, how much are these discontinuities affected by the choice of occupation-number cutoff, *T*_PNOcutoff_, used to obtain the PNO space for each occupied pair, and do we observe the expected convergence to the canonical-MO limit as this cutoff is tightened? How are the discontinuities and resulting properties affected by the choice of basis set; in particular, do diffuse functions, which are known to be important for accurate predictions of polarizabilities and hyperpolarizabilities, enhance or reduce the numerical instabilities.

To address these questions, we used central finite-difference expressions with fourth-order errors in the field strength to compute the energy derivatives corresponding to the electric dipole moment μ_*z*_

12 $$\mu_{z}=-{\frac{\partial E}{\partial F_{z}}|}_{F_{z}=0}=-\frac{-E\left(2F_{z}\right)+8E\left(F_{z}\right)-8E\left(-F_{z}\right)+E\left(-2F_{z}\right)}{12F_{z}}+O\left(F_{z}^{4}\right)$$

static dipole polarizability α_*zz*_

$$\alpha_{zz}=-{\frac{\partial^{2}E}{\partial F_{z}^{2}}|}_{F_{z}=0}=-\frac{-E\left(2F_{z}\right)+16E\left(F_{z}\right)-30E\left(0\right)+16E\left(-F_{z}\right)-E\left(-2F_{z}\right)}{12F_{z}^{2}}+O\left(F_{z}^{4}\right)$$ 13

and static dipole hyperpolarizability β_*zzz*_

14 $$\begin{array}{ll} \beta_{zzz} & =-{\frac{\partial^{3}E}{\partial F_{z}^{3}}|}_{F_{z}=0}\\ & =-\frac{-E\left(3F_{z}\right)+8E\left(2F_{z}\right)-13E\left(F_{z}\right)+13E\left(-F_{z}\right)-8E\left(-2F_{z}\right)+E\left(-3F_{z}\right)}{8F_{z}^{3}}+O\left(F_{z}^{4}\right) \end{array}$$

where the *z*-axis is as defined for each molecular structure in the Supporting Information. We have also examined expressions with lower-order errors, but our observations are similar; thus, we choose to use the above, which would normally be expected to yield small numerical errors compared to analytic differentiation. In accord with the field displacement sizes required for the numerical derivatives given above, all single-point-energy calculations were performed using external electric field strengths of ±0.001–0.1 au in increments of 0.001 au, from ±0.1–0.2 in increments of 0.002, and from ±0.2–0.3 in increments of 0.003. We note that we included electric field strengths that are typically too large for practical finite-field calculations to assess the questions for a variety of electric field strengths. For all chosen field strengths and directions, we carried out canonical-MO and LPNO-CCSD single-point energy calculations, with the latter using values of *T*_PNOcutoff_ ranging from 10^–6^ to 10^–10^. In addition, we employed tight convergence criteria for all Hartree–Fock SCF and CCSD iterative procedures: 10^–12^ in root-mean-squared density changes or amplitude increments. The carbon, oxygen, and fluoride 1*s* core orbitals were not frozen in most calculations, apart from specific tests to examine the impact of freezing the core on the resulting discontinuities. We used the correlation-consistent double-ζ basis sets of Dunning and co-workers^36–38^ both with and without diffuse functions (aug-cc-pVDZ and cc-pVDZ, respectively). We emphasize that our purpose in this work is to understand the origin and extent of discontinuities in the field-dependent LPNO-CCSD correlation energy, as well as the impact of those discontinuities on the properties described above when obtained through numerical differentiation. High-accuracy CC calculations that are intended for accurate predictions or comparison to experiment should be carried out with larger basis sets than those employed here and with freezing of core orbitals for basis sets designed to recover only valence–electron correlation effects.

All computations were carried out using the PyCC program, an open-source, Python-based pilot program that provides reference implementations for a range of CC methods.^39^ The code utilizes the Psi4 electronic structure package^40^ to compute the Hartree–Fock reference wave function, to localize the occupied orbitals using the Foster-Boys procedure,^32^ and to provide the MO-basis one- and two-electron integrals.

To identify the occurrence of discontinuities in the LPNO-CC correlation energy, we compute the average domain size across all occupied-orbital pairs, *n*, for each value of the external electric-field strength, *F*_*z*_

15 $${\mathrm{Q̅}}^{F_{z}}=\frac{\sum_{ij\in n}\dim\left(Q^{ij}\right)}{n}$$

Such discontinuities can be exacerbated when taking numerical derivatives of energies because of the need for multiple field strengths in a given finite-difference expression. To quantify such errors, we also report the average of the average domain sizes for all field strengths *F*_*z*_(*k*) required for a given numerical derivative

16 $${\mathrm{Q̿}}^{F_{z}}=\frac{\sum_{k}{\mathrm{Q̅}}^{F_{z}\left(k\right)}}{m}$$

where *m* is the number of single-point energies in the numerator of the expression. For example, for a given value of *F*_*z*_, ${\overset{®}{\overset{®}{Q}}}^{F_{z}}$ for the static dipole polarizability would be the average of ${\mathrm{Q̅}}^{F_{z}}$ , ${\mathrm{Q̅}}^{-F_{z}},{\mathrm{Q̅}}^{2F_{z}}$ , ${\mathrm{Q̅}}^{-2F_{z}}$ , and ${\mathrm{Q̅}}^{0F_{z}}$ [cf. eq 13].

## 4 Results and Discussion

In this section, we analyze LPNO-CCSD static electric properties in comparison to canonical-MO CCSD results. We will focus our attention on results computed using a value of *T*_PNOcutoff_ = 10^–7^ because it provides a representative example of the overall behavior of the method for each property. We first present the fully relaxed LPNO-CCSD correlation energy, dipole moment, static dipole polarizability, and static first dipole hyperpolarizability for H_2_O. We consider the impact of the choice of basis set and frozen-core on the frequency and magnitude of discontinuities in the correlation energy and its ultimate effect on these electric-field response properties. We then move forward to fluoroethylene (C_2_FH_3_) to examine the proliferation of these discontinuities as the size of the system/number of electrons increases. These two test cases establish a foundation for understanding the general behavior of the method. Additional results, including hypofluorous acid (HOF), *cis*-1,3-butadiene (C_4_H_6_), and PNO-relaxed calculations for water, are reported in the Supporting Information. Finally, although cutoff values were explored for all test cases, we present H_2_O with the aug-cc-pVDZ basis set as a representative example, illustrating how errors evolve as the cutoff is varied from 10^–6^ to 10^–10^. As shown in the Supporting Information, tightening this threshold reduces the discontinuity errors explored here, though at the cost of increased computational expense.

### 4.1 Water

Figure 1 presents correlation energies of water computed using CCSD (green dots) and LPNO-CCSD (orange dots) with the cc-pVDZ basis set as a function of external electric field strength. With the truncation at *T*_PNOcutoff_ = 1 × 10^–7^, the difference between the LPNO-CCSD and CCSD correlation energies is what we anticipate, viz. an accuracy within 99.9% for all field strengths with a maximum error of only 0.121 *mE*_*h*_. However, red dots on the LPNO-CCSD curve indicate discontinuities in the LPNO-CCSD correlation energy, all of which coincide with changes in the average domain size, ${\mathrm{Q̅}}^{F_{z}}$ , between consecutive values of *F*_*z*_ (blue dots using the right-hand axis). Quantifying these discontinuities as difference between the points (containing changes in the average domain size) of LPNO-CCSD correlation energies as well as the corresponding CCSD correlation energies then taking the difference in magnitude between the two differences (δ of LPNO-CCSD and δ of CCSD), the largest such discontinuity occurs between *F*_*z*_ = −0.129 and −0.130 au with a value of 6.09 μ*E*_*h*_, with the rest being within the same magnitude, which is roughly 3 orders of magnitude smaller than the localization errors at a given field strength. In addition, we observe more discontinuities in the correlation energies for positive field strengths than for negative. In the case of H_2_O, our choice of coordinate system (see the Supporting Information) places the oxygen atom on the positive *z*-axis and the hydrogen atoms on the negative *z*-axis, which means that a positive value of *F*_*z*_ produces a field oriented with the H_2_O dipole. This leads to an increase in the magnitude of the correlation energy as well as the total energy, which is dominated by the SCF component. (Total energies are provided in Section 6 of the Supporting Information.) The larger number of discontinuities is related to an increase in the sensitivity of the occupation numbers obtained from eq 5, and the central question is how these discontinuities affect the resulting properties obtained as numerical derivatives of the energy.

**Figure 1:**
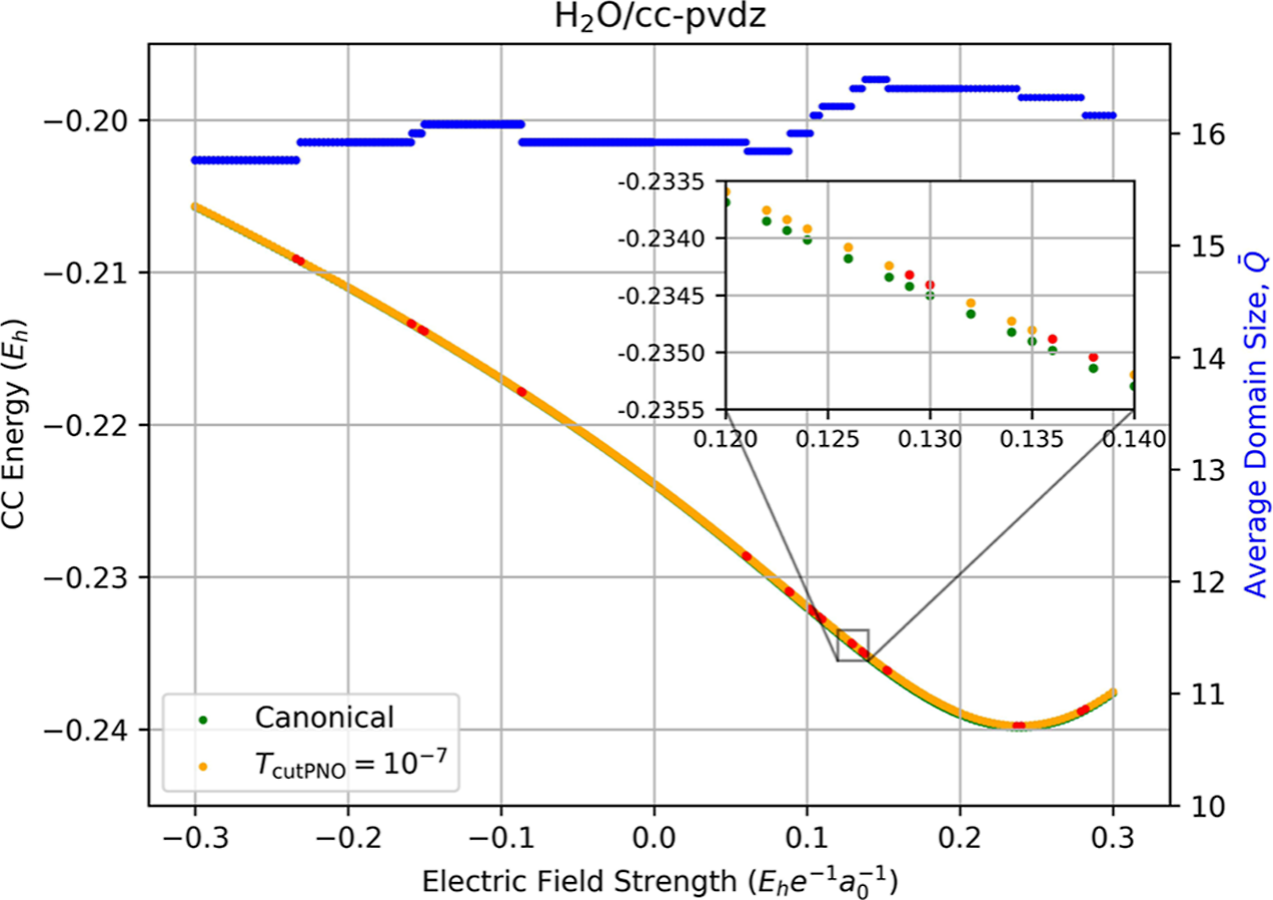
Fully relaxed correlation energies (left-hand axis) of water computed using canonical CCSD/cc-pVDZ (green dots) and LPNO-CCSD/cc-pVDZ (orange dots, *T*_PNOcutoff_ = 10^–7^) as a function of the external electric field strength (*F*_*z*_). Red dots indicate the occurrence of a change in the average domain sizes, ${\mathrm{Q̅}}^{F_{z}}$ , for the energies, which is indicated by the blue dots and the right-hand axis.

Figure 2 plots the correlation contribution to the water electric dipole moment as a function of the external electric field, again with green dots indicating the canonical-MO result and orange dots indicating LPNO-CCSD. The chosen orientation of the molecule along the *z*-axis yields a positive value of μ_*z*_ ≈ 0.07511 au (Total dipole moments are provided in Section 7 of the Supporting Information.) Numerical differentiation using weak field strengths (*F*_*z*_ < 0.01 au) yields a value of μ_*z*_ ≈ 0.07491 au, i.e. a localization error of roughly 2.0 × 10^–4^ au (0.27%). However, as we increase the field strength, we observe changes in the average domain size for the four energies needed for the derivative in eq 12 relative to their canonical-MO counterparts. (This “average of average domain sizes,” ${\overset{®}{\overset{®}{Q}}}^{F_{z}}$ , is indicated in Figure 2 by the blue dots using the right-hand axis.) These changes in the average domain size are small, and they yield discontinuities comparable to the localization errors in the resulting dipole moments. One of the largests errors occurs for a field strength of *F*_*z*_ = 0.079 au with a gap of approximately 7.53 × 10^–5^ au. The discontinuities in the LPNO-CCSD correlation energy thus produce insignificant errors in the dipole moment obtained via numerical derivatives compared to CCSD, at least for this small test case.

**Figure 2:**
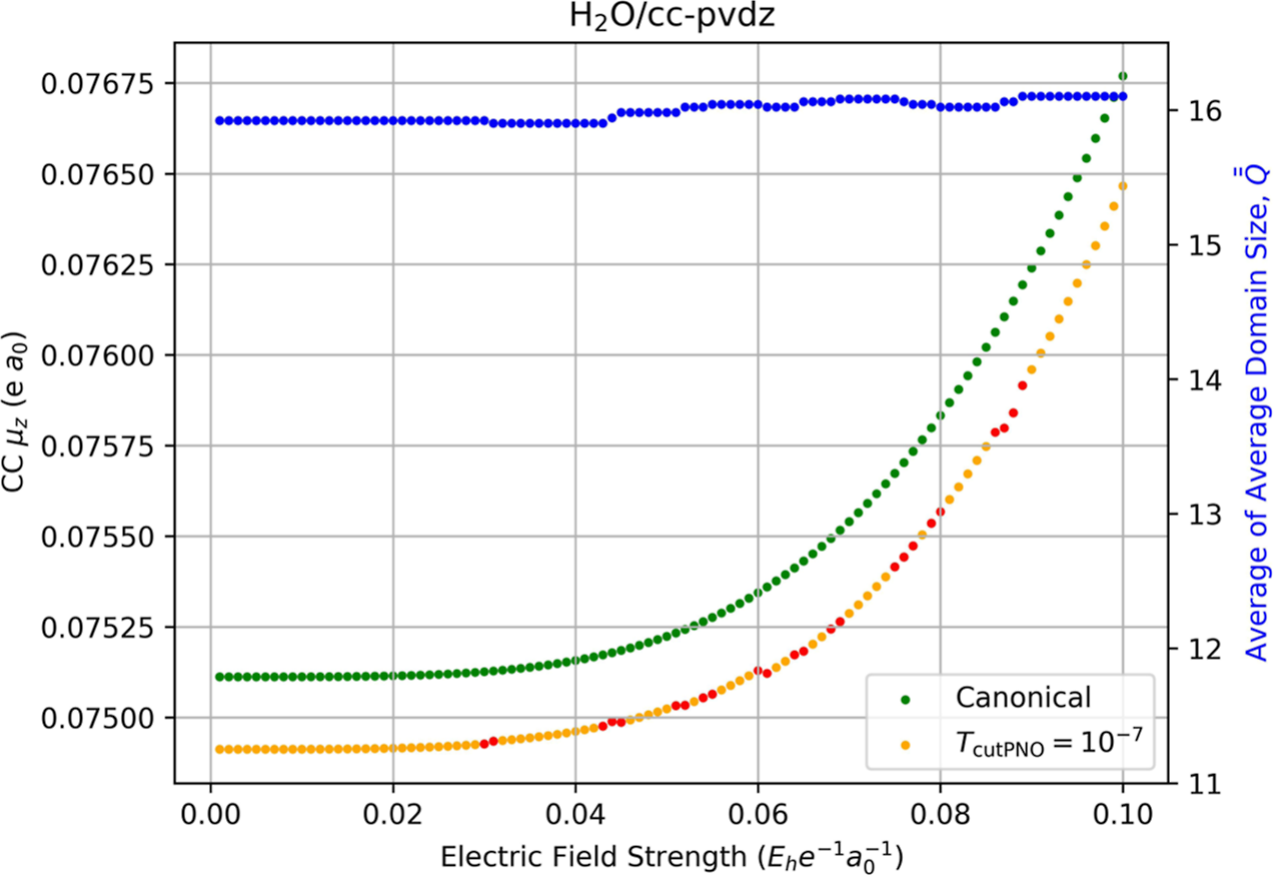
Fully relaxed correlation contribution to the electric dipole moment (left-hand axis) of water computed using canonical CCSD/cc-pVDZ (green dots) and LPNO-CCSD/cc-pVDZ (orange dots, *T*_PNOcutoff_ = 10^–7^) as a function of the external electric field strength (*F*_*z*_) used for numerical differentiation with eq 12. Red dots indicate the occurrence of a change in the average of average domain sizes, ${\overset{®}{\overset{®}{Q}}}^{F_{z}}$ , for the energies required for the derivative, which is indicated by the blue dots and the right-hand axis.

The impact of the discontinuities in the correlation energy becomes more apparent in higher-order properties as observed in Figures 3 and 4, which depict the correlation contributions to the polarizability and first hyperpolarizability, respectively. (Total polarizability and hyperpolarizability are provided in Sections 8 and 9 in the Supporting Information.) For the former, canonical-MO CCSD gives a value of α_*zz*_ = 0.1330 au, and, for weak field displacements (*F*_*z*_ < 0.03 au), the LPNO-CCSD approach yields α_*zz*_ = 0.1285 au for a localization error of ca. 3.5%. As the field displacement increases beyond 0.029 au, shifts in ${\overset{®}{\overset{®}{Q}}}^{F_{z}}$ appear due to the changes in pair-domain between the five energies needed for eq 13. (It is noteworthy that the values of ${\overset{®}{\overset{®}{Q}}}^{F_{z}}$ for α_*zz*_ in Figure 3 are nearly the same as those for μ_*z*_ in Figure 2. This occurs because of the similarity in the numerical differentiation expressions for the two properties; the field displacements needed for μ_*z*_ and α_*zz*_ are identical except that the latter also requires the *F*_*z*_ = 0.0 energy.) These shifts produce discontinuities in the computed value of α_*zz*_, with the largest appearing between *F*_*z*_ = 0.088 au and *F*_*z*_ = 0.089 au, where α_*zz*_ jumps from 0.1328 au to 0.1337 au, a gap of approximately 8.8 × 10^–4^ au.

**Figure 3:**
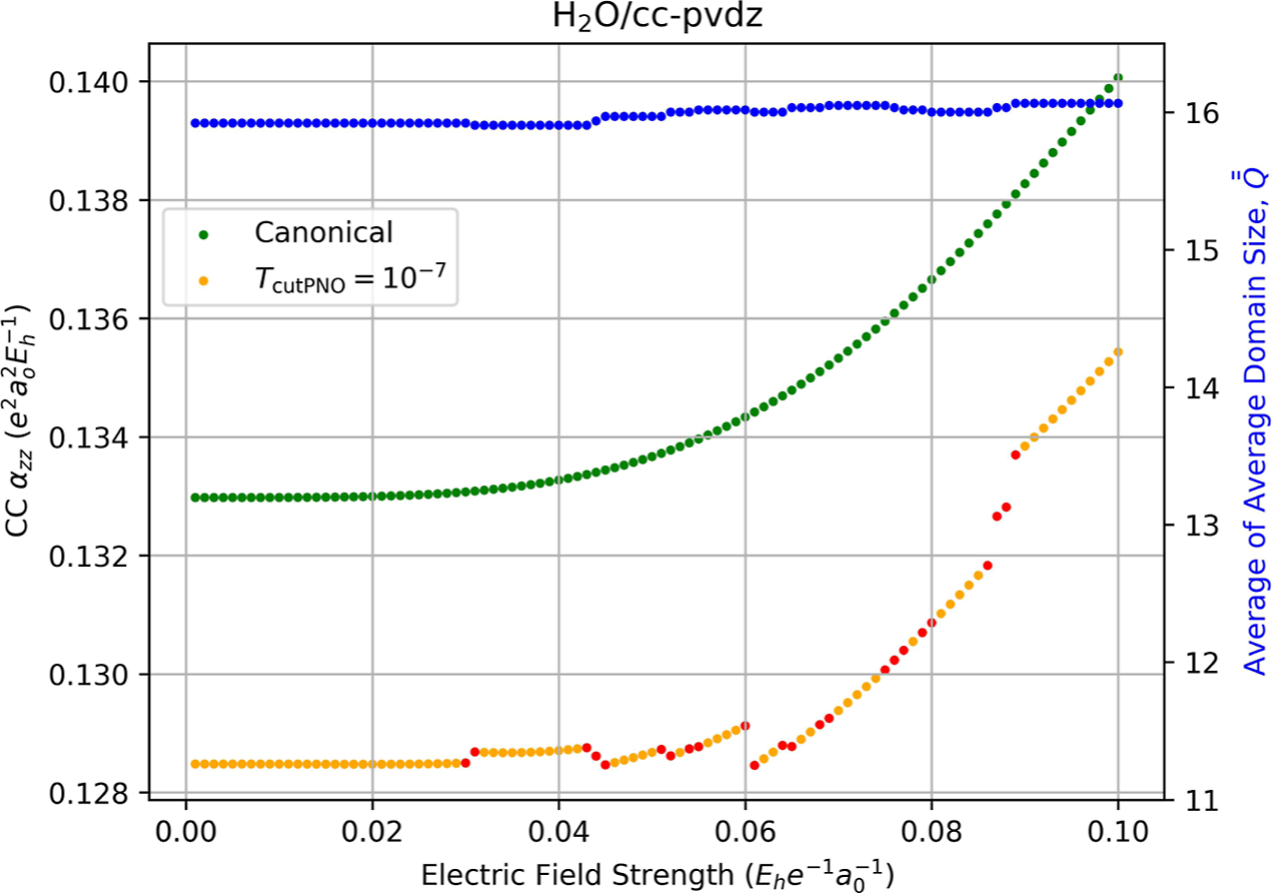
Fully relaxed correlation contribution to the static dipole polarizability (left-hand axis) of water computed using canonical CCSD/cc-pVDZ (green dots) and LPNO-CCSD/cc-pVDZ (orange dots, *T*_PNOcutoff_ = 10^–7^) as a function of the external electric field strength (*F*_*z*_) used for numerical differentiation with eq 13. Red dots indicate the occurrence of a change in the average of average domain sizes, ${\overset{®}{\overset{®}{Q}}}^{F_{z}}$ , for the energies required for the derivative, which is indicated by the blue dots and the right-hand axis.

**Figure 4:**
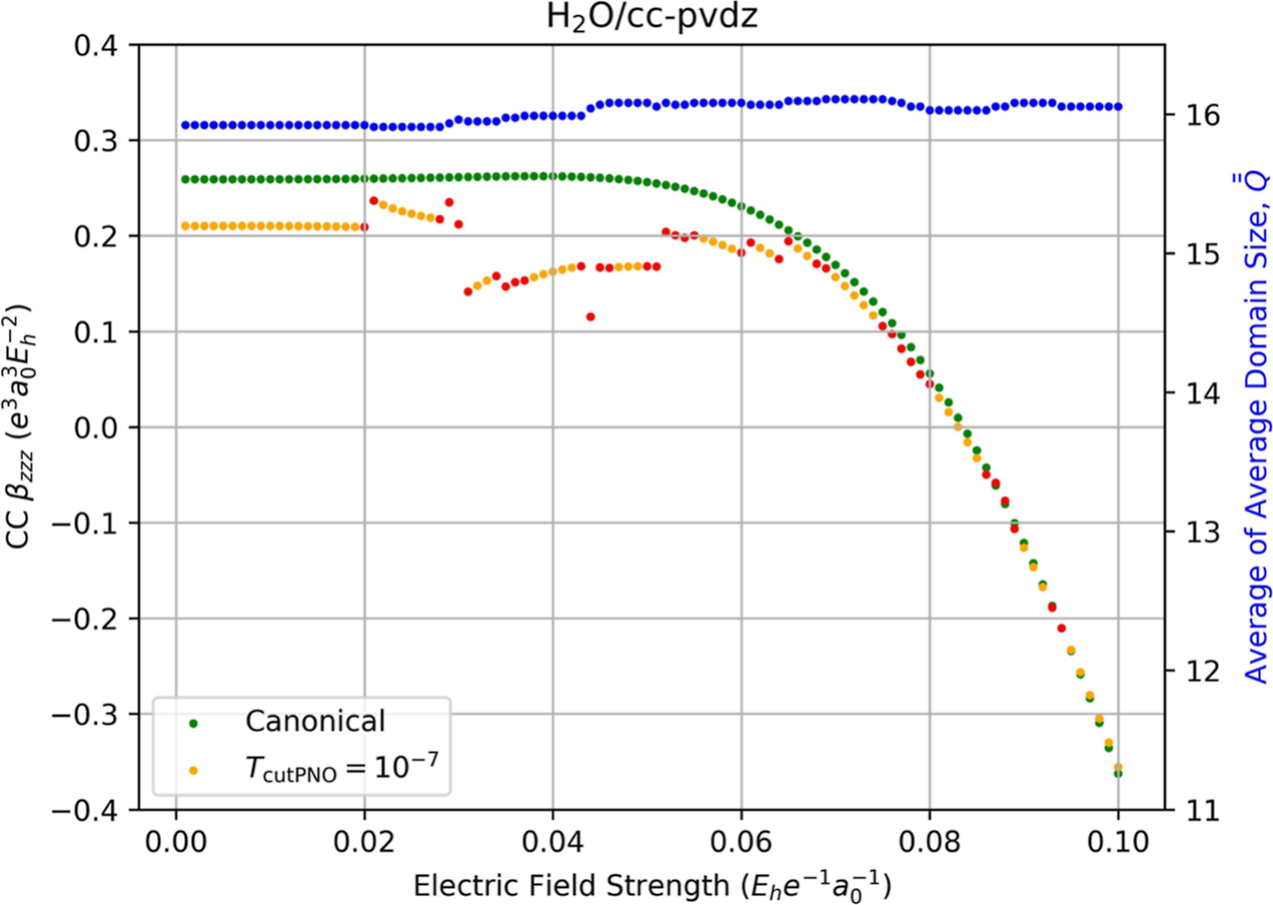
Fully relaxed correlation contribution to the static dipole hyperpolarizability (left-hand axis) of water computed using canonical CCSD/cc-pVDZ (green dots) and LPNO-CCSD/cc-pVDZ (orange dots, *T*_PNOcutoff_ = 10^–7^) as a function of the external electric field strength (*F*_*z*_) used for numerical differentiation with eq 14. Red dots indicate the occurrence of a change in the average of average domain sizes, ${\overset{®}{\overset{®}{Q}}}^{F_{z}}$ , for the energies required for the derivative, which is indicated by the blue dots and the right-hand axis.

For the correlation contributions to the hyperpolarizabilities in Figure 4, the canonical-MO CCSD value of β_*zzz*_ = 0.2590 au is numerically stable over a wide range of values of *F*_*z*_ used in eq 14. However, more discontinuities in the LPNO-CCSD values of β_*zzz*_ occur than for α_*zz*_ or μ_*z*_ because of the larger number of field displacements needed for the numerical derivative. For instance, the finite difference expression for the electric dipole moment, eq 12, requires four points along the *z*-axis of the external electric field, while the corresponding expression for the hyperpolarizability, eq 14, requires six points, resulting in more opportunities for the correlation domain structure to change for each given point and thus a higher chance for a discontinuity to occur. Futhermore, with a value of β_*zzz*_ = 0.2102 au, the localization error (ca. 18.9%, estimated using the field strengths below 0.02 au) is exacerbated for β_*zzz*_ significantly more than that observed for μ_*z*_ and α_*zz*_. Furthermore, for hyperpolarizabilities, the observed discontinuities can cause much greater errors than that of the localization error. For example, one of the largest discontinuities can be seen between *F*_*z*_ = 0.043 au and *F*_*z*_ = 0.044 au, with a gap of 0.053 au; the corresponding LPNO-CCSD value of β_*zzz*_ at *F*_*z*_ = 0.044 au is 0.1151 au while the canonical-MO CCSD results to 0.2611 au, i.e., the LPNO-CCSD value underestimates β_*zzz*_ by more than 50% at this particular point.

#### 4.1.1 Diffuse Basis Functions and Frozen Core Orbitals

It is well established that basis sets containing diffuse functions are important for accurate and robust predictions of field-response properties,^37,38,41–43^ such as polarizabilities and hyperpolarizabilities. Thus, it is important to consider the occurrence of field-dependent discontinuities with such basis sets. Figure 5 depicts the correlation contribution to the static hyperpolarizability using canonical-MO CCSD and LPNO-CCSD methods with the aug-cc-pVDZ basis set of Dunning and co-workers.^37,38^ For weak-field displacements, the canonical-MO CCSD value of β_*zzz*_ is approximately 0.06937 au and it remains relatively stable until the field strength exceeds ca. 0.03 au, at which point it begins to increase significantly. The corresponding value of β_*zzz*_ for LPNO-CCSD is unstable as discontinuities plague the weak field displacements (*F*_*z*_ < 0.018 au), with values ranging from −2750 to −0.6764 au, leading to a *y*-axis compression in the figure that masks the behavior at stronger fields. To capture the impact of discontinuities on higher field strengths, we have added an inset to the plot. We estimate the LPNO-CCSD value of β_*zzz*_ at *F*_*z*_ = 0.03 au to be 0.3740 au while that for canonical-MO CCSD is 0.4563 au, resulting in a localization error of ca. 22%.

**Figure 5:**
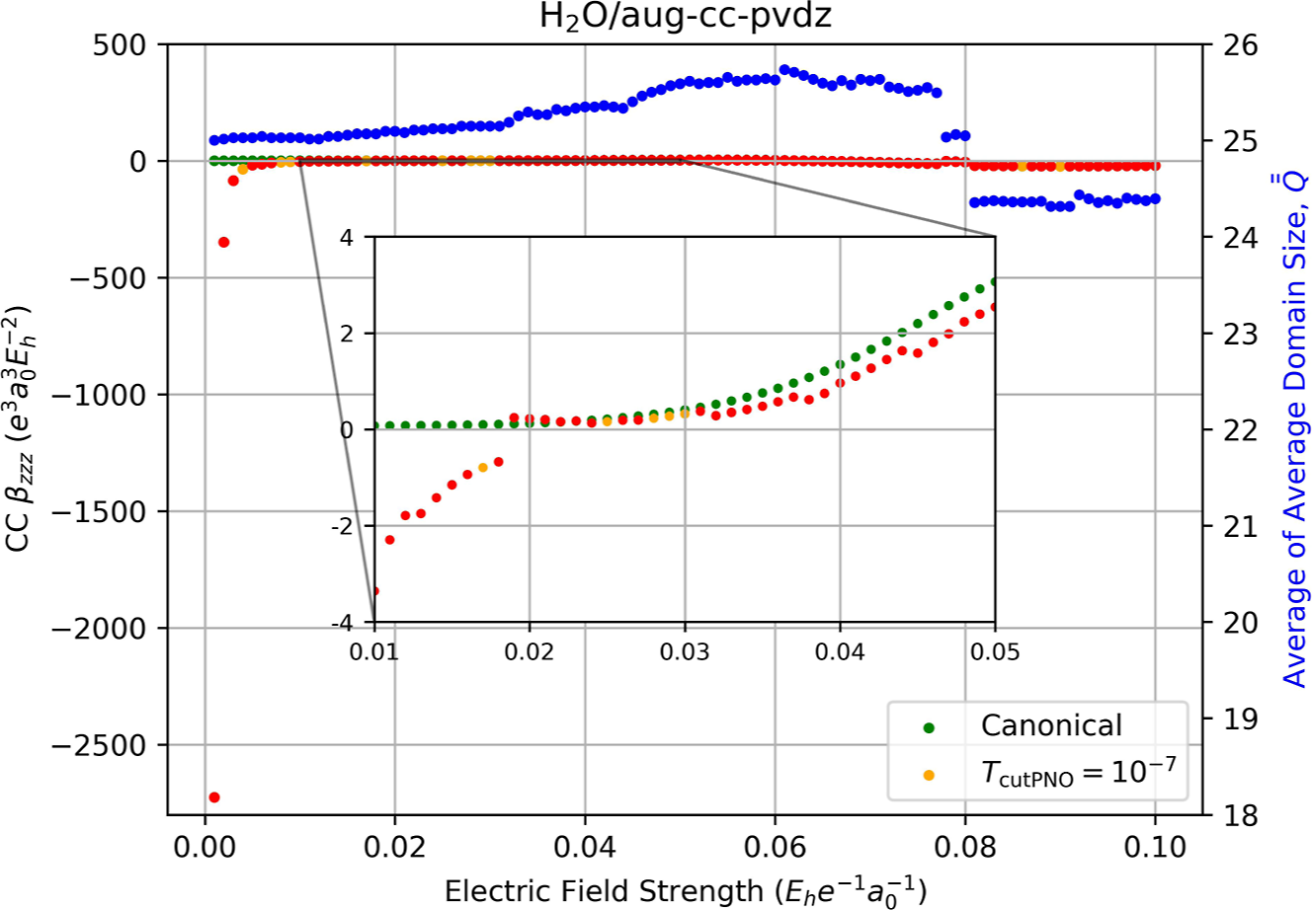
Fully relaxed correlation contribution to the static hyperpolarizability (left-hand axis) of water computed using canonical CCSD/aug-cc-pVDZ (green dots) and LPNO-CCSD/aug-cc-pVDZ (orange dots, *T*_PNOcutoff_ = 10^–7^) as a function of the external electric field strength (*F*_*z*_) used for numerical differentiation with eq 14. Red dots indicate the occurrence of a change in the average of average domain sizes, ${\overset{®}{\overset{®}{Q}}}^{F_{z}}$ for the energies required for the derivative, which is indicated by the blue dots and the right-hand axis.

We note two particularly large discontinuities that occur for both LPNO-CCSD and canonical-MO CCSD at stronger field strengths: the first jump occurring at *F*_*z*_ = 0.078 au and the second at *F*_*z*_ = 0.081 au These occur due to switching between Hartree–Fock SCF solutions induced by the stronger electric fields and yield even larger discontinuities in β_*zzz*_, as is evident from the correlation energies depicted in Figure S3. These switches impact only the hyperpolarizability in this case and not the lower order properties (see Figures S19 and S34) because the numerical derivative in eq 14 requires ±3 × *F*_*z*_ displacements, while μ_*z*_ in eq 12 and α_*zz*_ in eq 13 require ±2 × *F*_*z*_ displacements at most. Ignoring these two SCF-related discontinuities, much of the observed LPNO-CCSD instability appears to lessen at field strengths *F*_*z*_ > 0.018 au, but the inset reveals that large relative errors still persist. For example, at *F*_*z*_ = 0.032 au, the LPNO-CCSD value of β_*zzz*_ = 0.2814 au compared to canonical-MO CCSD value of β_*zzz*_ = 0.5164 au, an error of 46%.

The chaotic behavior observed above for LPNO-CCSD hyperpolarizabilities for weak-field displacements is not due to inherent numerical instability of the finite-difference approach, because they do not occur for the canonical-MO CCSD results. Instead, they are related to the relatively constant magnitude of the LPNO-CCSD localization error and the presence of discontinuities across a wide range of field strengths. The upper plot in Figure 6 depicts the localization error in the LPNO-CCSD correlation energy relative to canonical-MO CCSD to highlight discontinuities that occur as a function of the electric-field strength for extremely weak fields, while the lower plot shows the correlation contribution to the hyperpolarizability over the same range of field strengths. In both figures we have chosen field increments of 0.000005 au up to 0.0001 au, followed by increments of 0.000025 au up to 0.001 au, though in the upper plot the corresponding field strengths of ±*F*_*z*_, ±2*F*_*z*_, and ±3*F*_*z*_ are included because these are needed for eq 14. Over this range of weak field strengths, the upper plot reveals only one discontinuity in the energy at *F*_*z*_ = 0.000755 which occurs due to a change in the average dimensionality of the LPNO space, $\overset{®}{\overset{®}{Q}}$ , from 24.96 to 25.04 virtual PNOs.

**Figure 6:**
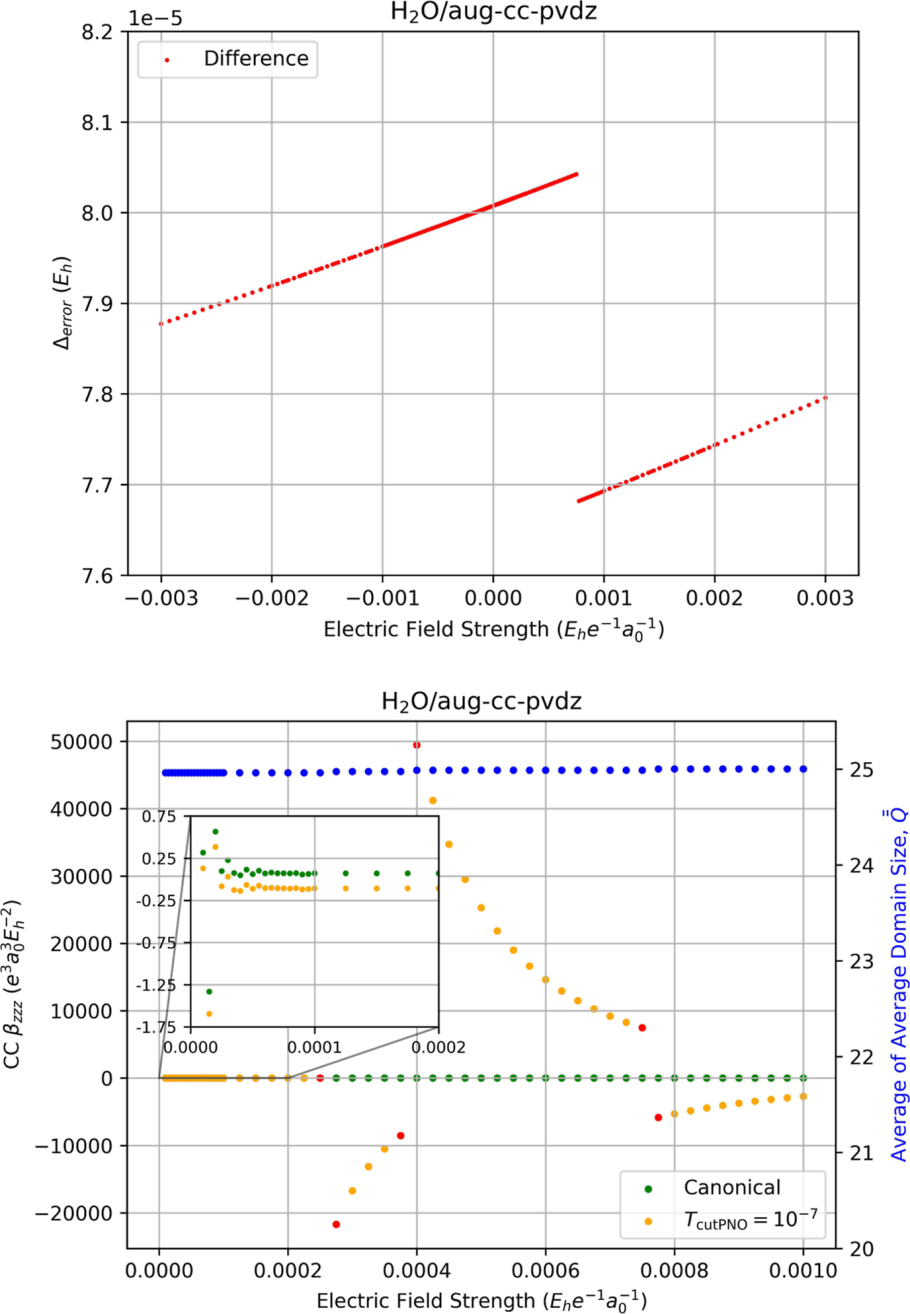
Upper figure: The difference between LPNO-CCSD/aug-cc-pVDZ and canonical-MO CCSD/aug-cc-pVDZ correlation energies of water. Lower-figure: the fully relaxed correlation-energy contribution to the static hyperpolarizability (left-hand axis) of water computed using canonical CCSD/aug-cc-pVDZ (green dots) and LPNO-CCSD/aug-cc-pVDZ (orange dots, *T*_PNOcutoff_ = 10^–7^) as a function of the external electric field strength (*F*_*z*_) used for numerical differentiation with eq 14. Red dots indicate the occurrence of a change in the average of average domain sizes, ${\overset{®}{\overset{®}{Q}}}^{F_{z}}$ , for the energies required for the derivative, which is indicated by the blue dots and the right-hand axis.

In the hyperpolarizabilities in the lower plot, we observe numerical instability at extremely weak fields (*F*_*z*_ < 0.00006 au), which, as the inset plot in the figure reveals, arises for both the canonical-MO CCSD and LPNO-CCSD hyperpolarizabilities due to the limit of the numerical differentation even for the tight convergence criteria used here. For field strengths above 0.00006 au, we also observe three discontinuities at *F*_*z*_ = 0.000275, 0.000400, and 0.000775, all of which occur due to the discontinuity in the LPNO-CCSD energy at *F*_*z*_ = 0.000755 au That is, in accord with eq 14 the value of β_*zzz*_ at each of these points require energies on each side of the discontinuity in Figure 6 (lower). For example, the hyperpolarizability computed at *F*_*z*_ = 0.000400 au requires the LPNO-CCSD energies at 2 × *F*_*z*_ = 0.000800 and 3 × *F*_*z*_ = 0.001600 au, both of which are beyond the discontinuity and thus introduce an error in the numerator of eq 14. Furthermore, the magnitude of the error is exacerbated by the small field displacement in the denominator because *F*_*z*_^3^ = 2.1 × 10^–11^ au. The combination of these two factors yields dramatic errors in the values of β_*zzz*_ compared to the canonical-MO CCSD value of ca. 0.06938 au: at *F*_*z*_ = 0.000275, 0.000400, and 0.000775 au, LPNO-CCSD yields β_*zzz*_ = −27,623, +49,451, and −5856 au, respectively. This behavior is also observed for tighter cutoffs (see Section 5 of the Supporting Information), though the effects of the discontinuity naturally become less pronounced as the LPNO-CCSD converges to the canonical-MO CCSD limit.

Returning to the cc-pVDZ basis set while freezing the 1*s* core MO on the oxygen atom yields reduces the number of discontinuities we observe in all of the properties considered here. For the correlation energies, for example, freezing the core orbital reduces the number of discontinuities from 13 to seven (cf. S1 and S8), primarily because of the reduction in the number of occupied pairs relative to the all-electron calculation. While the reduction of discontinuities is evident in the correlation contribution of the hyperpolarizability, plotted in Figure 7, we still observe large discontinuity errors, e.g. at *F*_*z*_ = 0.027 au with a LPNO-CCSD value of β_*zzz*_ = 0.1636 au compared to canonical-MO CCSD value of β_*zzz*_ = 0.2610 au, an error of 37%. Interestly, the localization error in regions far from a discontinuity diminishes (e.g., at weak field strengths) and matches the canonical-MO value of 0.2594 au up to the fourth decimal places. We also note that the “average of average domain sizes,” ${\overset{®}{\overset{®}{Q}}}^{F_{z}}$ , metric does not capture all the discontinuities. For example, a gap of 0.026 au can be seen in β_*zzz*_ between *F*_*z*_ = 0.053 and 0.054 au even though ${\overset{®}{\overset{®}{Q}}}^{F_{z}}$ stays consistent at 18.82 virtual PNOs due to identical averaging over changes in PNO dimensionality between the two.

**Figure 7:**
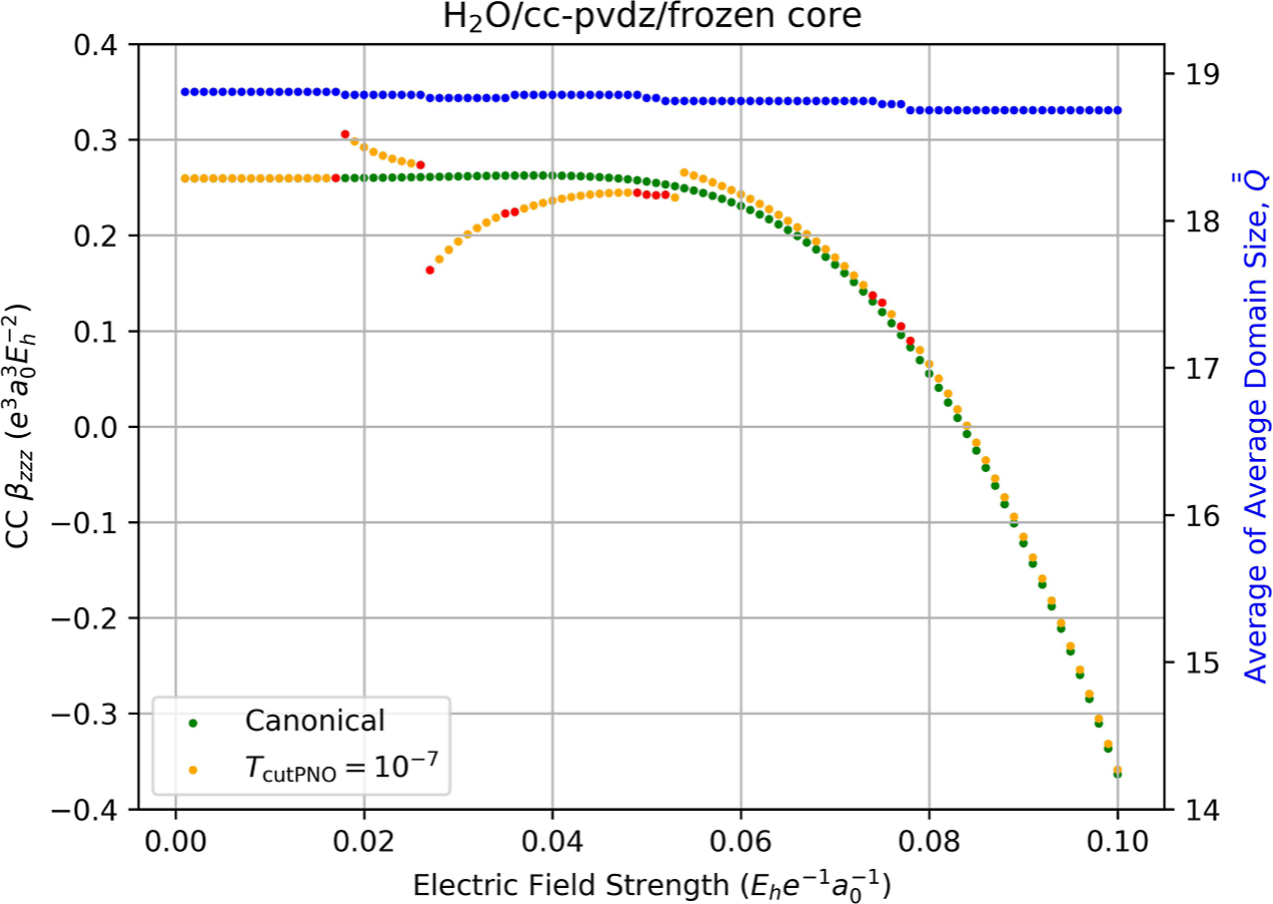
Fully relaxed frozen-core correlation contribution to the static hyperpolarizability (left-hand axis) of water computed using canonical CCSD/cc-pVDZ (green dots) and LPNO-CCSD/cc-pVDZ (orange dots, *T*_PNOcutoff_ = 10^–7^) as a function of the external electric field strength (*F*_*z*_) used for numerical differentiation with eq 14. Red dots indicate the occurrence of a change in the average of average domain sizes, ${\overset{®}{\overset{®}{Q}}}^{F_{z}}$ , for the energies required for the derivative, which is indicated by the blue dots and the right-hand axis.

If we continue to freeze the 1*s* core but switch back to the aug-cc-pVDZ basis, the number of discontinuities in the correlation contribution to the hyperpolarizability again increases dramatically, as indicated in Figure 8. The corresponding values of LPNO-CCSD β_*zzz*_ are still plagued by discontinuities, though over a slightly smaller range from −2096 to −0.2238 au for the weak-field displacements (*F*_*z*_ < 0.018 au). In addition, we observe a similar localization error of 30% (LPNO-CCSD β_*zzz*_ of 0.3633 au vs a canonical-MO CCSD value of 0.5185 au). Note that we obtained β_*zzz*_ values for LPNO-CCSD only up to *F*_*z*_ = 0.076 au due to the lack of convergence of single-point field-dependent energies beyond 2 × *F*_*z*_ = 0.154 au in this case.

**Figure 8:**
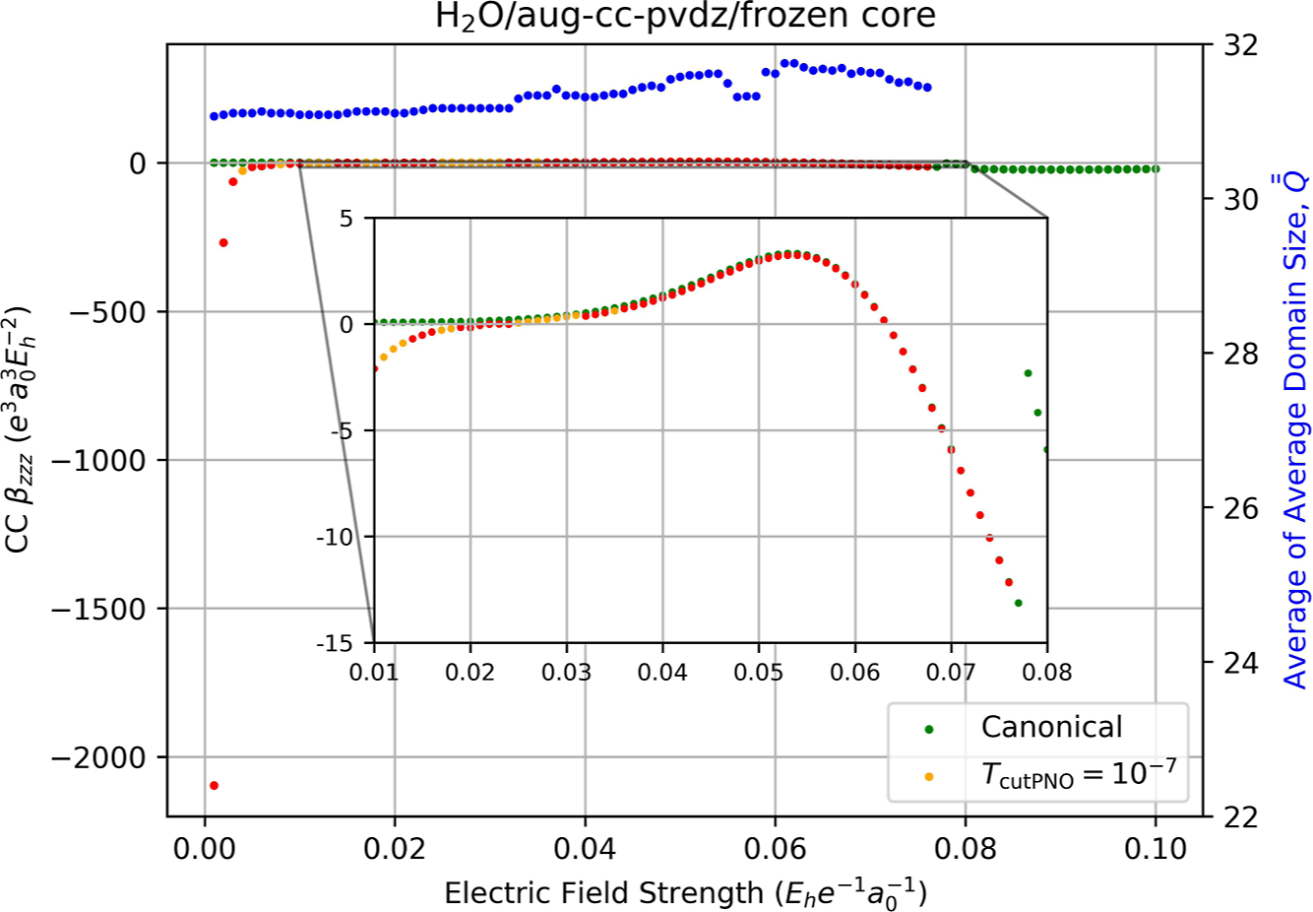
Fully relaxed frozen-core correlation contribution to the static hyperpolarizability (left-hand axis) of water computed using canonical CCSD/aug-cc-pVDZ (green dots) and LPNO-CCSD/aug-cc-pVDZ (orange dots, *T*_PNOcutoff_ = 10^–7^) as a function of the external electric field strength (*F*_*z*_) used for numerical differentiation with eq 14. Red dots indicate the occurrence of a change in the average of average domain sizes, ${\overset{®}{\overset{®}{Q}}}^{F_{z}}$ , for the energies required for the derivative, which is indicated by the blue dots and the right-hand axis.

### 4.2 Fluoroethylene

In order to investigate further the appearance and impact of discontinuities in the LPNO-CCSD correlation energy with the strength of an external electric field, we consider fluoroethylene (C_2_FH_3_), a strongly polar and polarizable molecule. Figure S12 depicts the correlation energy of C_2_FH_3_ in the cc-pVDZ basis as a function of the external electric field strength, *F*_*z*_. Discontinuities in the correlation energy occur at almost every step along the curve, and, while most retain the expected accuracy of within 99.9% compared to the canonical-MO CCSD values, they lead to magnification of errors in the numerical derivatives.

Figure 9 plots the static polarizability of fluoroethylene as a function of the field strength, *F*_*z*_, used for the finite-field differentiation in eq 13. Similar to H_2_O with an aug-cc-pVDZ basis set, the use of weak field strengths (*F*_*z*_ < 0.007 au) produces larger discrepancies compared to the canonical-MO CCSD value of α_*zz*_ = −0.0956 au, ranging from −0.1590 au to 0.0641 au Indeed, the second weakest choice of *F*_*z*_ = 0.002 au produces the largest error of 0.1597 au (167%) between the canonical-MO CCSD and LPNO-CCSD α_*zz*_ data. The values stabilize somewhat at approximately *F*_*z*_ = 0.007 au, where the error between the canonical-MO CCSD and LPNO-CCSD β_*zzz*_ is approximately 15.7%. For larger field displacements, the errors fluctuate unpredictably between 16.6% (*F*_*z*_ = 0.012 au) and 0.001% (*F*_*z*_ = 0.02 au).

**Figure 9:**
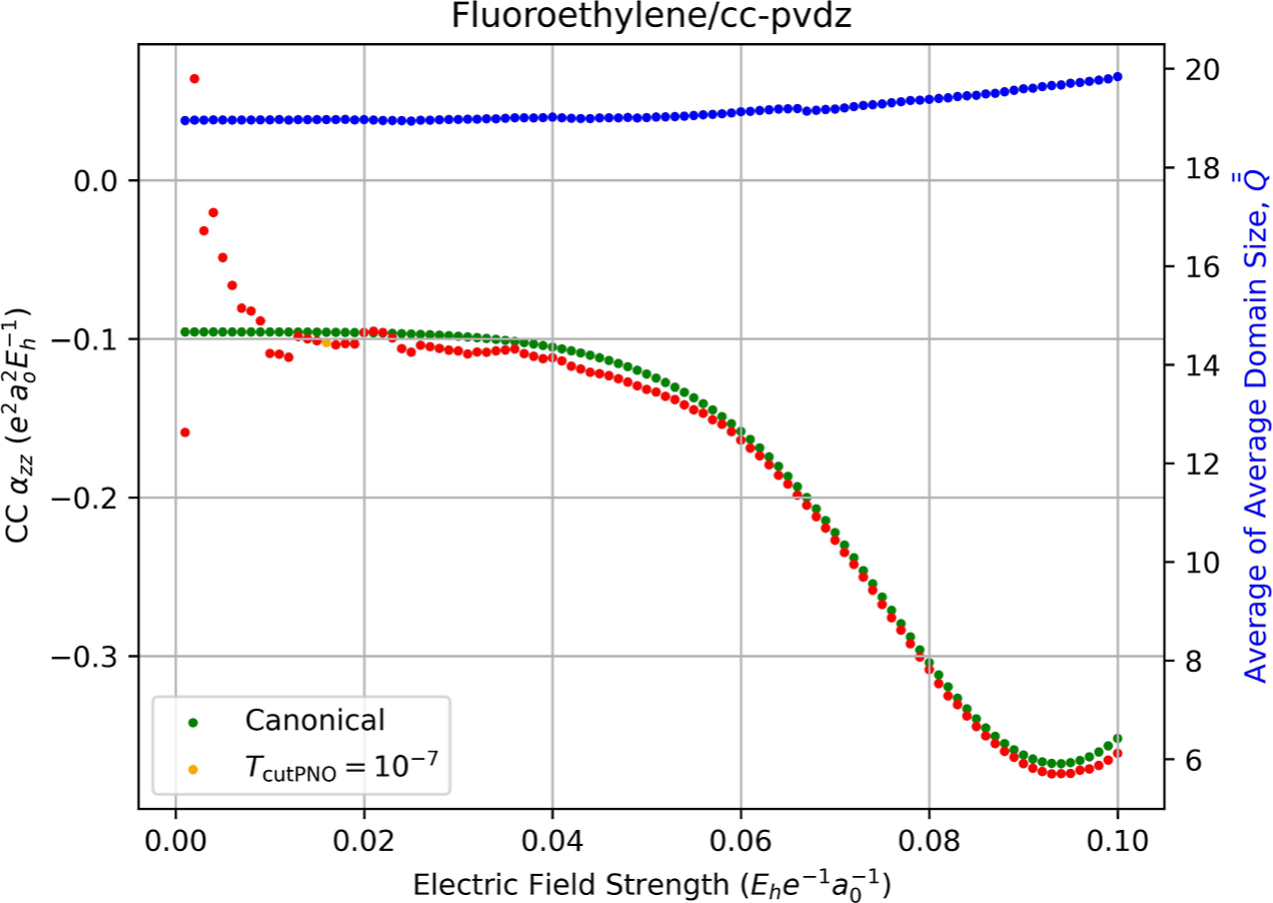
Fully relaxed correlation contribution to static dipole polarizability (left-hand axis) of fluoroethylene computed using canonical CCSD/cc-pVDZ (green dots) and LPNO-CCSD/cc-pVDZ (orange dots, *T*_PNOcutoff_ = 10^–7^) as a function of the external electric field strength (*F*_*z*_) used for numerical differentiation with eq 13. Red dots indicate the occurrence of a change in the average of average domain sizes, ${\overset{®}{\overset{®}{Q}}}^{F_{z}}$ , for the energies required for the derivative, which is indicated by the blue dots and the right-hand axis.

The correlation contribution to the static hyperpolarizabilities of fluoroethylene, depicted in Figure 10, exhibit larger errors. The LPNO-CCSD static hyperpolarizability ranges from 520.47 to −11.41 au for *F*_*z*_ < 0.006 au, fluctuating around the canonical-MO CCSD value of β_*zzz*_ = 1.7086 au Larger field displacements exhibit greater stability, though the errors are still significant, e.g. at *F*_*z*_ = 0.018 au where LPNO-CCSD overestimates the canonical-MO CCSD value of β_*zzz*_ = 1.6855 au by more than 100% with a β_*zzz*_ of 3.529 au For field strengths exceeding *F*_*z*_ = 0.025 au, most of the errors are within 15% of the canonical-MO CCSD results, though such fields also introduce larger errors from the numerical differentiation for both canonical-MO CCSD and LPNO-CCSD as compared to the weak-field values of β_*zzz*_. Between *F*_*z*_ = 0.042 and 0.044 au the errors are roughly 25% of the canonical-MO CCSD β_*zzz*_ values while those at strongest field strengths considered here (*F*_*z*_ < 0.093 au) can deviate more than 100%, e.g. *F*_*z*_ = 0.098 au Futhermore, similar to H_2_O, inclusion of diffuse functions further increase the number and magnitude of discontinuity errors for fluoroethylene, as shown for the aug-cc-pVDZ basis set in Figure S58.

**Figure 10:**
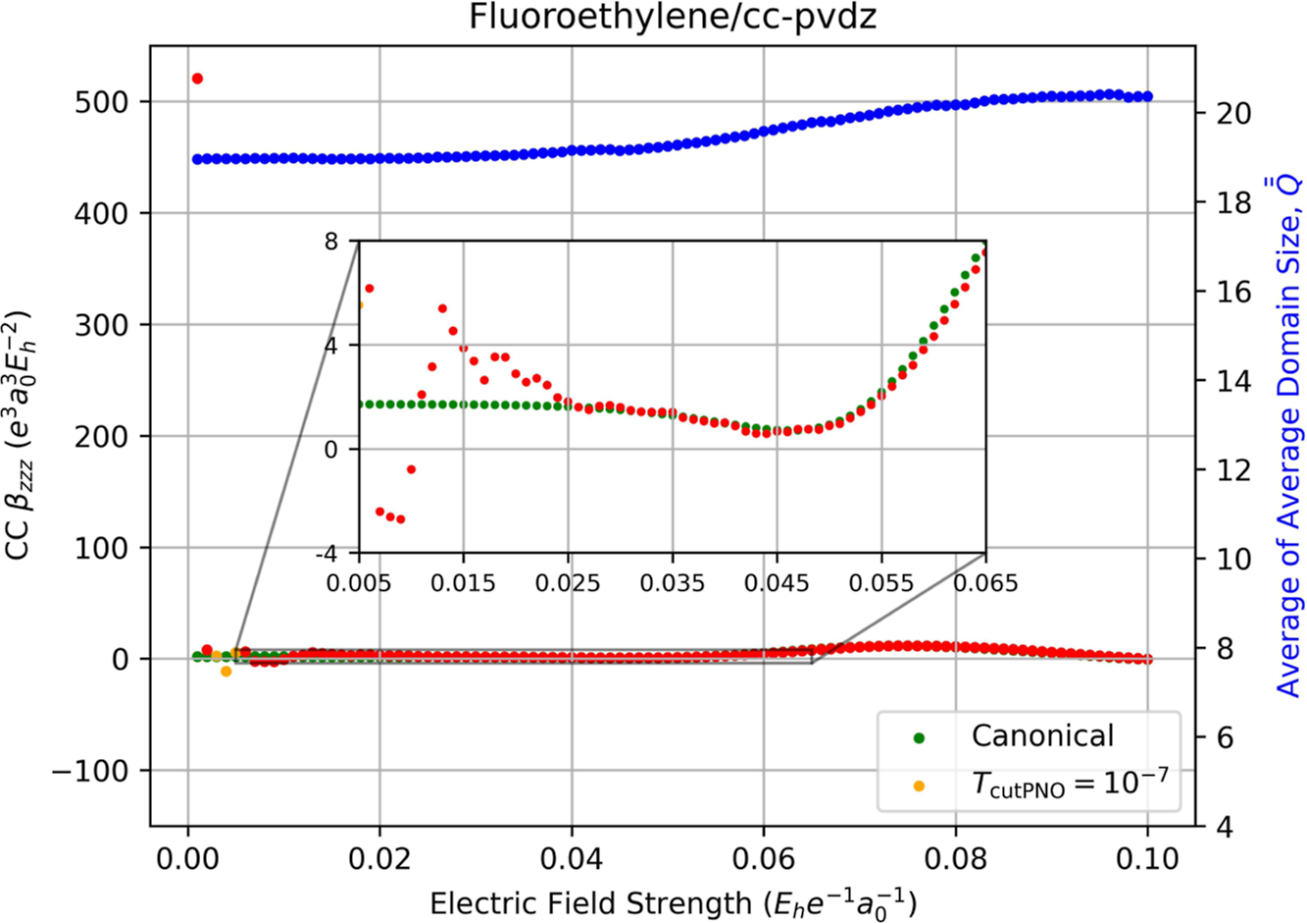
Fully relaxed correlation contribution to static dipole hyperpolarizability (left-hand axis) of fluoroethylene computed using canonical CCSD/cc-pVDZ (green dots) and LPNO-CCSD/cc-pVDZ (orange dots, *T*_PNOcutoff_ = 10^–7^) as a function of the external electric field strength (*F*_*z*_) used for numerical differentiation with eq 14. Red dots indicate the occurrence of a change in the average of average domain sizes, ${\overset{®}{\overset{®}{Q}}}^{F_{z}}$ , for the energies required for the derivative, which is indicated by the blue dots and the right-hand axis.

### 4.3 Do Fixed PNO-Space Dimensions Resolve the Discontinuity Problem?

As demonstrated above, the appearance of discontinuities in LPNO-CCSD correlation energies as a function of the external electric field strength and in properties obtained as numerical derivatives of such energies is due to the variation in the dimension of the virtual PNO spaces with field displacements. Thus, one approach to overcoming this error might be to freeze the dimensions of the PNO spaces based on a reference calculation such as the zero-field dimensions. Figure 11 depicts the results of such a method for the hyperpolarizability of H_2_O using the cc-pVDZ basis set where the average domain size, ${\mathrm{Q̅}}^{F_{z}}$ , is fixed to the *F*_*z*_ = 0 dimensions for all field displacements. Comparison to Figure 4 reveals a clear reduction in the number of magnitude of the discontinuities appearing in the LPNO-CCSD β_*zzz*_ curves, and the LPNO-CC localization error in the hyperpolarizabilities relative to the canonical-MO CCSD values is approximately roughly 19%, which is close to that observed earlier for the variable-field LPNO-CCSD for H_2_O in the same basis set. Nevertheless, some small discontinuities remain, such as that between *F*_*z*_ = 0.052 and 0.053 au, where a small jump in the LPNO-CCSD β_*zzz*_ appears from 0.1862 to 0.1805 au, a gap of 0.0057 au.

**Figure 11:**
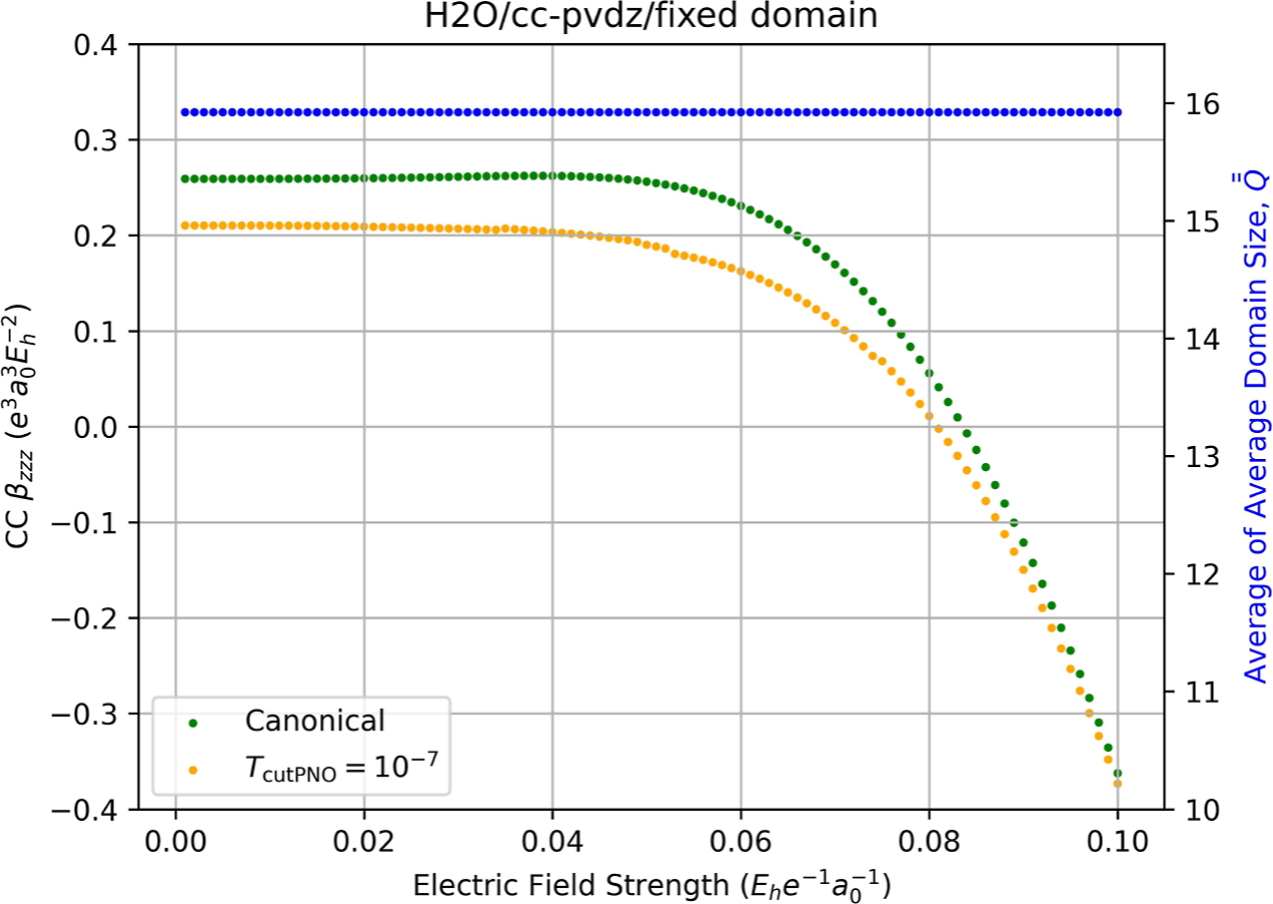
Fully relaxed correlation contribution to static dipole hyperpolarizability (left-hand axis) of water computed using canonical-MO CCSD/cc-pVDZ (green dots) and LPNO-CCSD/cc-pVDZ (orange dots, *T*_PNOcutoff_ = 10^–7^) as a function of the external electric field strength (*F*_*z*_) used for numerical differentiation with eq 14. The domain size of each occupied pair is fixed based on a zero field calculation. Red dots indicate the occurrence of a change in the average of average domain sizes, ${\overset{®}{\overset{®}{Q}}}^{F_{z}}$ , for the energies required for the derivative, which is indicated by the blue dots and the right-hand axis.

Increasing the basis set to aug-cc-pVDZ also increases the number of discontinuities, as shown in Figure 12 for the hyperpolarizability of H_2_O, though the number of such skips is reduced relative to the variable-field results depicted in Figure 5. Interestingly, the use of fixed PNO domains also shifts the direction of the LPNO-CCSD localization error yielding the incorrect sign of β_*zzz*_. Compared with the canonical-MO results, the largest error (62%) occurs at *F*_*z*_ = 0.022 au where the LPNO-CCSD hyperpolarizability is −0.2455 au compared to the canonical-MO CCSD value of 0.1509 au.

**Figure 12:**
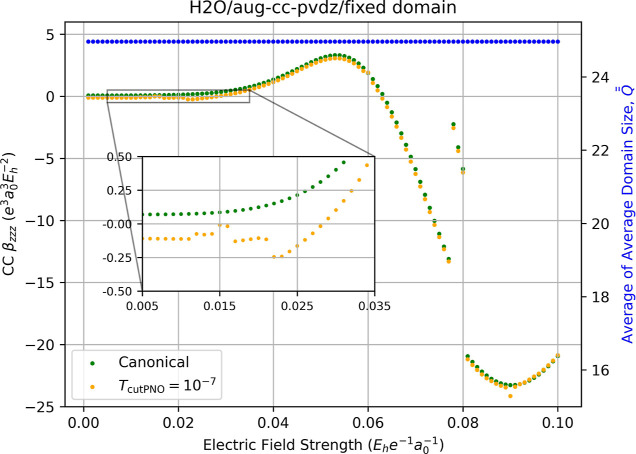
Fully relaxed correlation contribution to static dipole hyperpolarizability (left-hand axis) of water computed using canonical CCSD/aug-cc-pVDZ (green dots) and LPNO-CCSD/aug-cc-pVDZ (orange dots, *T*_PNOcutoff_ = 10^–7^) as a function of the external electric field strength (*F*_*z*_) used for numerical differentiation with eq 14. The domain size of each occupied pair is fixed based on a zero field calculation. Red dots indicate the occurrence of a change in the average of average domain sizes, ${\overset{®}{\overset{®}{Q}}}^{F_{z}}$ , for the energies required for the derivative, which is indicated by the blue dots and the right-hand axis.

For fluoroethylene, the errors are pervasive across a wider range of field displacements for fixed-domain vs variable-domain LPNO-CCSD. Examination of the cc-pVDZ hyperpolarizabilities in Figure 13 reveals numerical instabilities up to *F*_*z*_ = 0.053 au with β_*zzz*_ values ranging from −225.9 to 155.7 au with most values one to 2 orders of magnitude larger than the canonical-MO CCSD value of 2.037 au.

**Figure 13:**
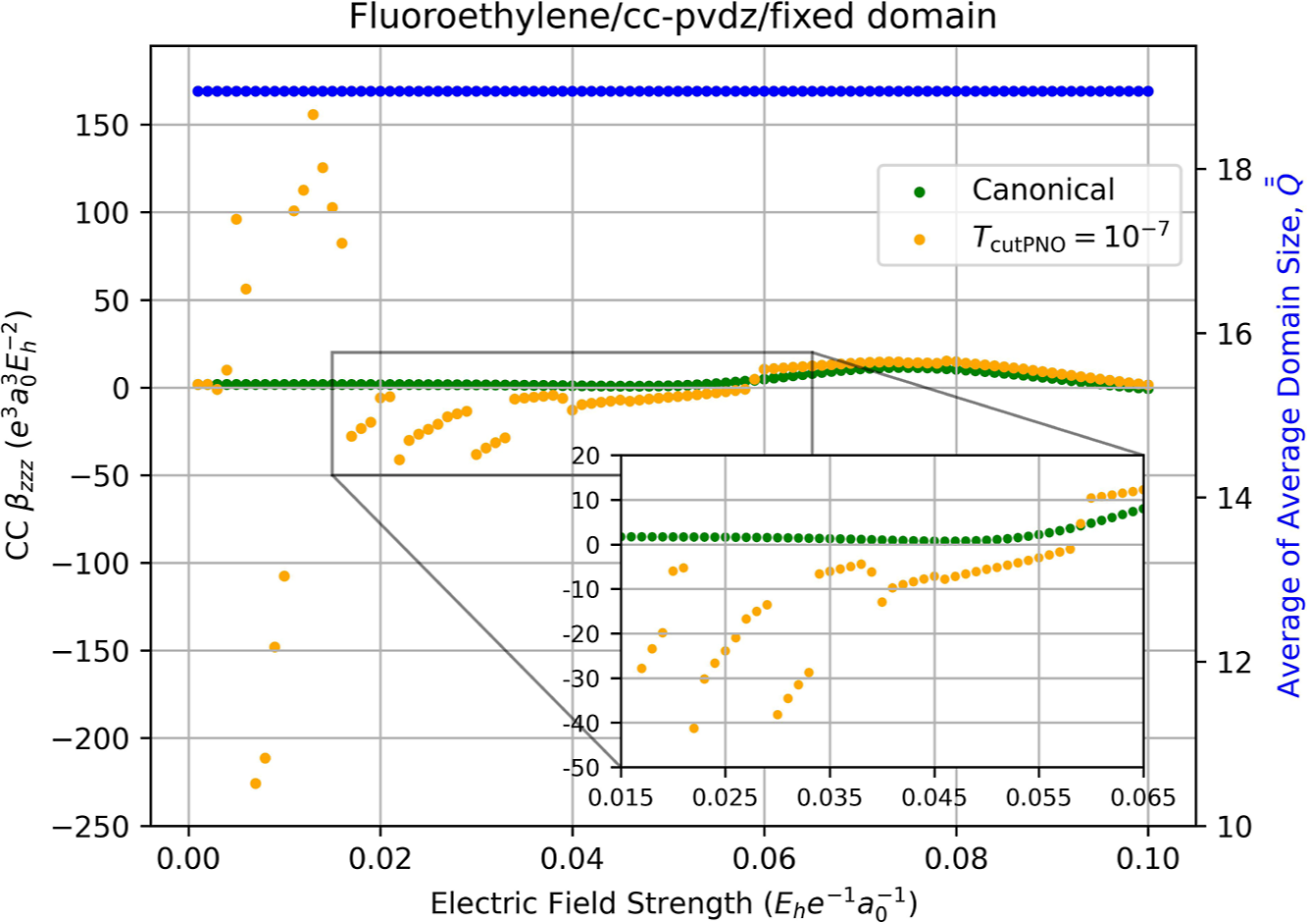
Fully relaxed correlation contribution to static dipole hyperpolarizability (left-hand axis) of fluoroethelene computed using canonical CCSD/aug-cc-pVDZ (green dots) and LPNO-CCSD/aug-cc-pVDZ (orange dots, *T*_PNOcutoff_ = 10^–7^) as a function of the external electric field strength (*F*_*z*_) used for numerical differentiation with eq 14. The domain size of each occupied pair is fixed based on a zero field calculation. Red dots indicate the occurrence of a change in the average of average domain sizes, ${\overset{®}{\overset{®}{Q}}}^{F_{z}}$ , for the energies required for the derivative, which is indicated by the blue dots and the right-hand axis.

The fundamental reason for the failure of both the fixed- and variable-domain approaches is that the *ordering* and the *composition* of the PNOs can vary considerably with even small changes in the external field strength. To illustrate this, consider the overlap between the zero-field PNOs and their field-dependent counterparts, i.e.

17 $$S_{ij}^{0^{\prime }}={\left[Q_{ij}^{0}\right]}^{T}{\left[C^{0}\right]}^{T}SC^{\prime }Q_{ij}^{\prime }$$

where ***S*** is the atomic orbital overlap matrix, ***C*** is the MO coefficient matrix, ***Q***_*ij*_ is the MO-to-PNO transformation matrix for occupied-MO pair *ij*, and the superscripts 0 and ′ denote *F*_*z*_ = 0 and *F*_*z*_ ≠ 0, respectively. If both the left- and right-hand PNOs and MOs are identical (e.g., both at zero field), then ***S***_*ij*_ is simply the identity matrix, but as the field varies the overlap matrix reveals the nature of differences in the relevant PNOs.

Figures 14 and 15 provide heatmaps of PNO overlaps for fluoroethylene in the cc-pVDZ basis set for two occupied-MO pairs, *ij* = 39 and 143, both of which are diagonal pairs (i.e., *i* = *j*). In each heatmap, the row index corresponds to *F*_*z*_ = 0 PNOs and the column index to *F*_*z*_ = ± 0.022, 2 × *F*_*z*_ = ± 0.044, or 3 × *F*_*z*_ = ± 0.066 au. The blue lines indicate the truncation point of the PNO space for *T*_PNOcutoff_ = 10^–7^ such that the PNOs to the right of the vertical blue line or below the horizontal blue line would be those retained in a subsequent LPNO-CC calculation.

**Figure 14:**
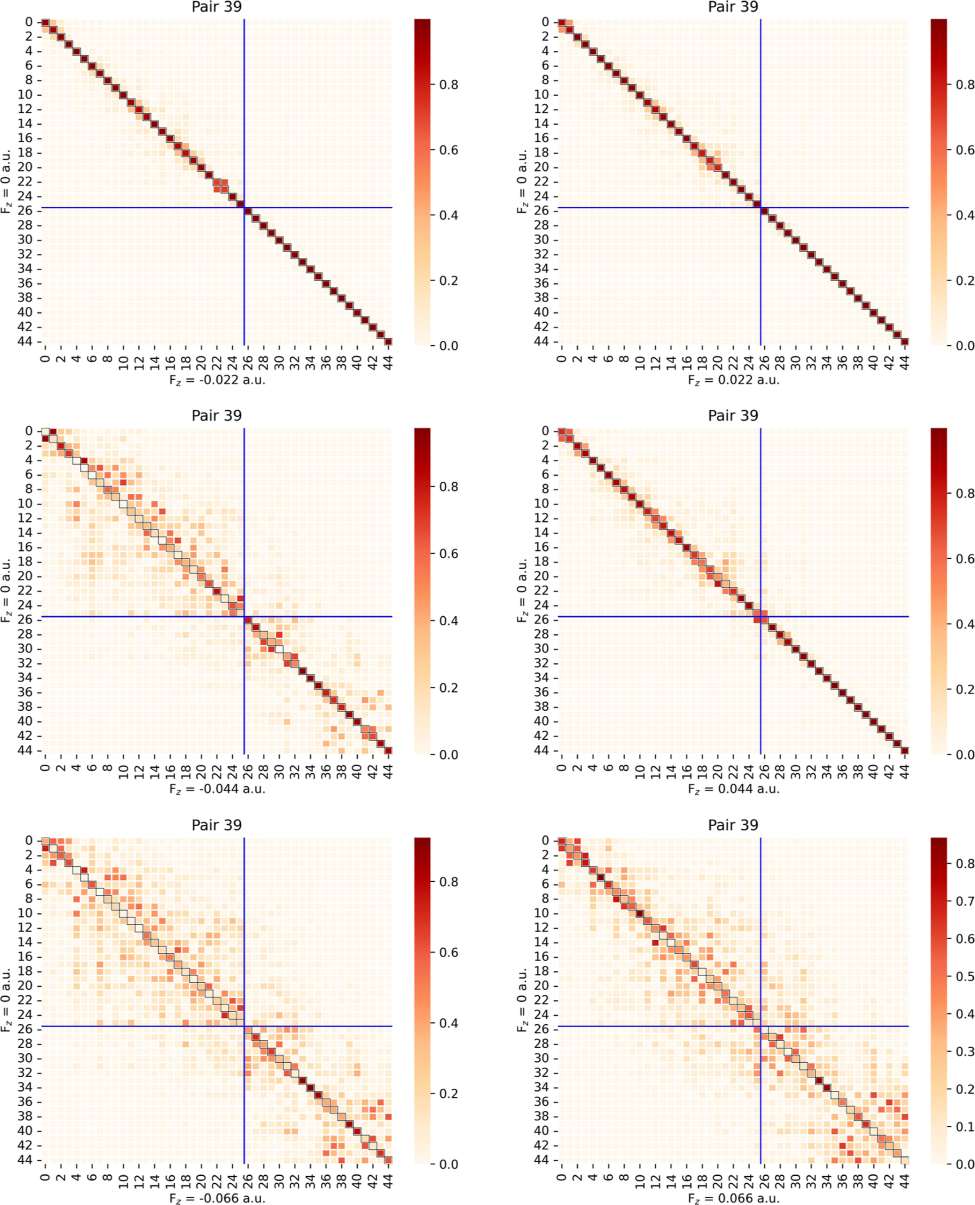
Heatmaps of PNO overlaps between *F*_*z*_ = 0 and *F*_*z*_ = ± 0.022 (first row), ±0.044 (second row), and ±0.066 au (third row) for localized occupied MO pair 39 of fluoroethylene with the cc-pVDZ basis set. The lower right quadrant demarcated by the blue cross represents the truncated space at *T*_PNOcutoff_ = 10^–7^.

**Figure 15:**
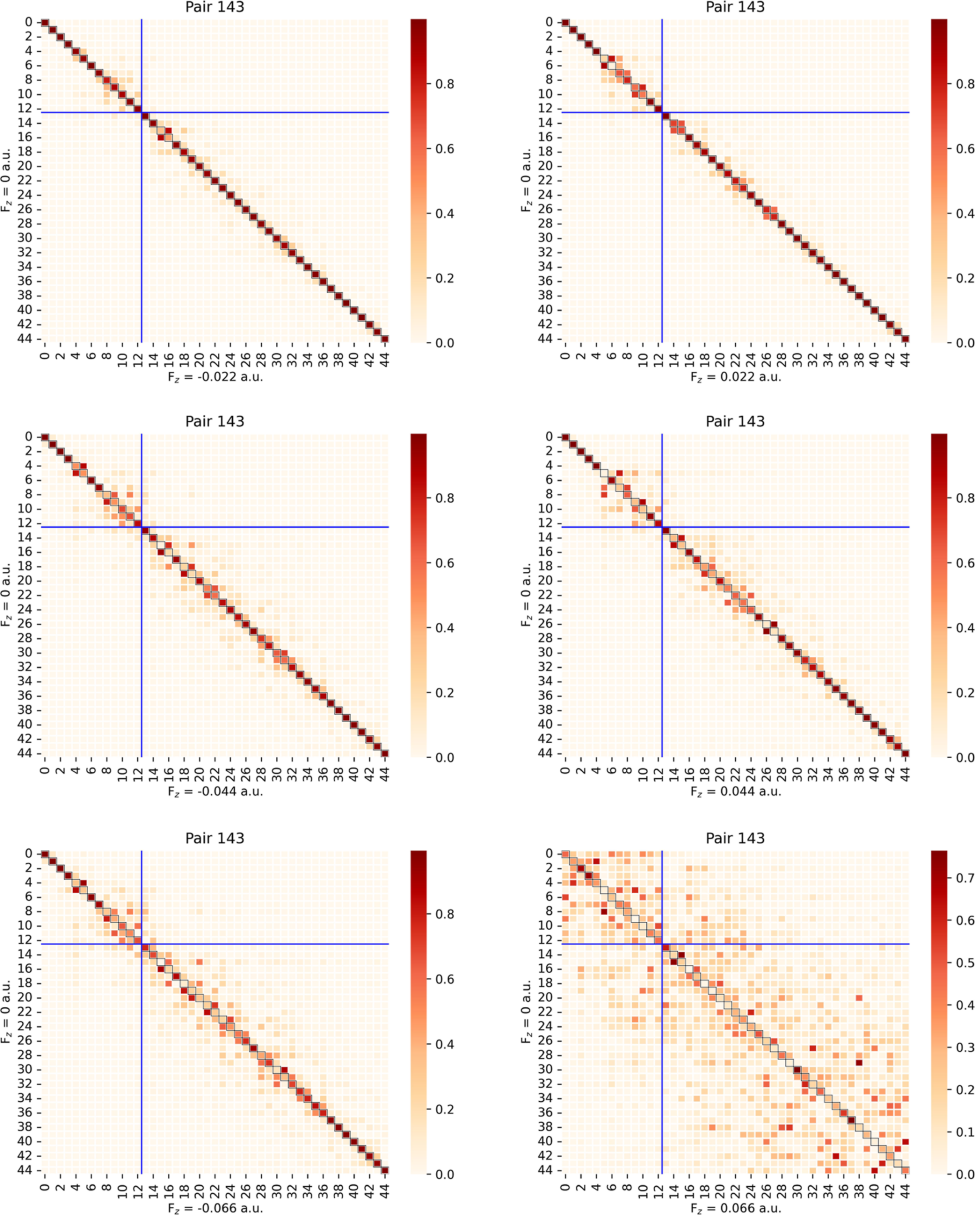
Heatmaps of PNO overlaps between *F*_*z*_ = 0 and *F*_*z*_ = ± 0.022 (first row), ±0.044 (second row), and ±0.066 au (third row) for localized occupied MO pair 143 of fluoroethylene with the cc-pVDZ basis set. The lower right quadrant demarcated by the blue cross represents the truncated space at *T*_PNOcutoff_ = 10^–7^.

For the overlaps of *ij* = 39 in Figure 14, the smaller field displacements of *F*_*z*_ = ± 0.022 au do not significantly alter the dominant diagonal terms, and for those below the PNO cutoff (blue lines at PNO 26) the overlap is essentially the identity matrix, though some mixing of the truncated vs retained *F*_*z*_ ≠ 0 PNOs still occurs relative to their zero-field counterparts, as it indicated by the small off-diagonal overlap values near the crossing point of the blue lines. For PNOs 0–25 that would be removed in a truncated LPNO-CC calculation, we can see considerable mixing, particularly PNOs 22 and 23 for *F*_*z*_ = −0.022 and PNOs 19 and 20 for *F*_*z*_ = 0.022 au For the *F*_*z*_ = ±0.022 overlaps of *ij* = 143 in Figure 15, the mixings in the retained space (PNOs 14–44) are much larger, though the mixings of retained and truncated PNOs are still very small.

As the external field displacements increase to 2 × *F*_*z*_ = ±0.044 au, we can observe significantly more mixing of the PNOs for both *ij* = 39 and 143, including between the retained and truncated PNOs. This indicates more substantial changes in the character of the PNOs as the electric field increases relative to the zero-field PNOs. In the case of *ij* = 39 (Figure 14) for *F*_*z*_ = 0.044 au, we can also observe almost 50/50 mixing between PNOs 25 and 26, which straddle the truncation cutoff for *T*_PNOcutoff_ = 10^–7^. Thus, the space spanned by the *F*_*z*_ = 0 au PNOs and the *F*_*z*_ = 0.044 au PNOs are clearly not the same in this case. Finally, for the largest field displacements considered here *F*_*z*_ = ±0.066 au, the differences relative to the *F*_*z*_ = 0 au PNOs become even more pronounced, especially for *ij* = 143 (Figure 15) and *F*_*z*_ = 0.066 au In that case, while the overlap matrix is still diagonally dominant overall, many of the diagonal matrix elements are near-zero, indicating both mixing of the PNOs and swapping in occupation-number ordering. We note that the LPNO-CCSD energies are invariant to rotations within the active PNO space and that any discontinuities result from rotations between the active and inactive PNO domains (i.e, the off-diagonal blocks in Figures 14 and 15).

These dramatic changes in the space spanned by a subset of the PNOs for even weak fields relative to those obtained at *F*_*z*_ = 0, are inherent to the PNO-based methods because the eigenvalues (occupation numbers) of the virtual-MO pair-densities are naturally near-zero. This leads to extremely large condition numbers (ratios of the maximum and minimum eigenvalues), indicating the potential for numerical stability of linear system and/or eigenvalue equations. (For pair-densities *ij* = 39 and 143, for example, the condition numbers are 3.4 × 10^8^ and 6.0 × 10^6^, respectively.) Thus, simply fixing the dimensions of the PNO space for each pair of occupied orbitals cannot overcome such variations, and the energy and derivative-property discontinuities reported here will thus persist.

## 5 Conclusions

We have examined the occurrence and impact of discontinuities in the correlation energy obtained via the LPNO-CC method arising due to variations in the dimensions of the pairwise correlation domains with changes in the strength of the external electric field. Using several polar molecules as test cases (water, fluoroethylene, hypofluorous acid and *cis*-1,3-butadiene), we find that, although the absolute magnitudes of the energy discontinuities are small (approximately 1 μ*E*_*h*_), they neverthelesss result in large errors in higher-order electric-field-dependent response properties, such as polarizabilities and hyperpolarizabilities.

While the smallest test case, H_2_O with a cc-pVDZ basis set, contains discontinuties that may be smaller than the localization errors for the electric dipole moment and polarizability, the computed hyperpolarizability underestimates the canonical-MO CCSD by ca. 50%. As the system size and number of electrons increase, more discontinuities occur and can be multiple orders of magnitude larger (and yield incorrect signs), requiring very tight and impractical PNO cutoffs to reduce the errors. Diffuse basis sets, which are necessary for obtaining reliable results for acceptable comparison to experiment, tend to exacerbate the problem. In addition, energy discontinuities arise over a wide range of field strengths, including small electric-field displacements commonly used for the computation of properties using numerical derivatives. Indeed, for very weak fields for which the finite-difference approach yields numerically stable canonical-MO CCSD properties, the LPNO-CCSD method can yield hyperpolarizabilities in error by several orders of magnitude.

We have attempted to correct this behavior by freezing the dimensionality of each pair-domain at their zero-field values. However, we do not find this approach to be reliable because the external fields induce significant mixing of the natural orbitals relative to their *F*_*z*_ = 0 counterparts. This exquisite sensitivity of the PNO eigenvalue problem arises due to the high condition numbers of the pairwise densities, which naturally exhibit numerous near-zero eigenvalues. As a result, large discontinuities and associated errors persist, especially for larger test cases. Another possible direction of exploration might be to determine subsets of field-dependent PNOs with minimal principal angles^44^ with the zero-field PNOs, though this is beyond the scope of the current work. The use of analytic first-order properties (dipole moments), such as that developed by Neese and co-workers,^35^ in the numerical differentiation rather than correlation energies could reduce this problem somewhat. However, the discontinuities would still be present and would still plague, e.g., second hyperpolarizabilities, such as those computed by Naim et al.^29^

It is worth noting that such discontinuities would occur for PNO++ and similar methods, though we have not explicitly investigated them in this work. Field-dependent discontinuities could also appear for PAO-based local-correlation methods because the field would influence the construction of the virtual orbital space through changes in the underlying SCF MOs. However, the sensitivity of the PAO-CC orbital domains to such changes is not obvious because the construction of such domains is based on a distance-dependent criterion rather than natural-orbital occupation numers. In addition, all of these methods are also subject to the appearance of discontinuities due to changes in external magnetic fields and/or nuclear displacements, where the latter affect the exploration of potential eneergy hypersurfaces, as discussed earlier by Russ and Crawford.^45^

## Supplementary Material



## Notes

Structures and plots of energies, dipole moments, polarizabilities, and hyperpolarizabilities for H_2_O, HOF, C_2_FH_3_, and C_4_H_6_ for varying choices of *T*_PNOcutoff_ are provided, along with all computed correlation energies, total energies, and average pair domains for all electric-field strengths (PDF)

## References

[1] Meyer W. (1973). PNO–CI
Studies of electron correlation effects. I. Configuration expansion
by means of nonorthogonal orbitals, and application to the ground
state and ionized states of methane. J. Chem.
Phys..

[2] Edmiston C., Krauss M. (1966). Pseudonatural orbitals
as a basis for the superposition
of configurations. I. He_2_
^+^. J. Chem. Phys..

[3] Edmiston C., Krauss M. (1968). Pseudonatural orbitals
as a basis for the superposition
of configurations. II. Energy surface for linear H_3_. J. Chem. Phys..

[4] Werner H.-J., Meyer W. (1976). PNO-CI and PNO-CEPA
studies of electron correlation effects. Mol.
Phys..

[5] Ahlrichs R., Keil F., Lischka H., Kutzelnigg W., Staemmler V. (1975). PNO–CI (pair natural-orbital
configuration interaction)
and CEPA–PNO (coupled electron pair approximation with pair
natural orbitals) calculations of molecular systems. III. The molecules
MgH_2_, AlH_3_, SiH_4_, PH_3_ (planar
and pyramidal), H_2_S, HCl, and the Ar atom. J. Chem. Phys..

[6] Neese F., Hansen A., Liakos D. G. (2009). Efficient
and accurate approximations
to the local coupled cluster singles doubles method using a truncated
pair natural orbital basis. J. Chem. Phys..

[7] Neese F., Wennmohs F., Hansen A. (2009). Efficient and accurate local approximations
to coupled-electron pair approaches: An attempt to revive the pair
natural orbital method. J. Chem. Phys..

[8] Hättig C., Tew D. P., Helmich B. (2012). Local explicitly correlated second-
and third-order Møller-Plesset perturbation theory with pair
natural orbitals. J. Chem. Phys..

[9] Krause C., Werner H.-J. (2012). Comparison of explicitly correlated local coupled-cluster
methods with various choices of virtual orbitals. Phys. Chem. Chem. Phys..

[10] Ma Q., Schwilk M., Köppl C., Werner H.-J. (2017). Scalable Electron
Correlation Methods. 4. Parallel Explicitly Correlated Local Coupled
Cluster with Pair Natural Orbitals (PNO-LCCSD-F12). J. Chem. Theory Comput..

[11] Frank M. S., Hättig C. (2018). A pair natural orbital based implementation of CCSD
excitation energies within the framework of linear response theory. J. Chem. Phys..

[12] Werner H.-J., Knizia G., Krause C., Schwilk M., Dornbach M. (2015). Scalable Electron
Correlation Methods I.: PNO-LMP2 with Linear Scaling in the Molecular
Size and Near-Inverse-Linear Scaling in the Number of Processors. J. Chem. Theory Comput..

[13] Werner H.-J. (2016). Communication:
Multipole approximations of distant pair energies in local correlation
methods with pair natural orbitals. J. Chem.
Phys..

[14] Ma Q., Schwilk M., Köppl C., Werner H.-J. (2018). Correction to Scalable
Electron Correlation Methods. 4. Parallel Explicitly Correlated Local
Coupled Cluster with Pair Natural Orbitals (PNO-LCCSD-F12). J. Chem. Theory Comput..

[15] Krause C., Werner H.-J. (2019). Scalable Electron
Correlation Methods. 6. Local Spin-Restricted
Open-Shell Second-Order Møller–Plesset Perturbation Theory
Using Pair Natural Orbitals: PNO-RMP2. J. Chem.
Theory Comput..

[16] Kats D., Werner H.-J. (2019). Multi-state local
complete active space second-order
perturbation theory using pair natural orbitals (PNO-MS-CASPT2). J. Chem. Phys..

[17] Ma Q., Werner H.-J. (2019). Accurate Intermolecular
Interaction Energies Using
Explicitly Correlated Local Coupled Cluster Methods [PNO-LCCSD­(T)-F12]. J. Chem. Theory Comput..

[18] Ma Q., Werner H.-J. (2020). Scalable
Electron Correlation Methods. 7. Local Open-Shell
Coupled-Cluster Methods Using Pair Natural Orbitals: PNO-RCCSD and
PNO-UCCSD. J. Chem. Theory Comput..

[19] Gauss, J. In Encyclopedia of Computational Chemistry; Schleyer, P. , Allinger, N. L. , Clark, T. , Gasteiger, J. , Kollman, P. A. , Schaefer, H. F., III , Schreiner, P. R. , Eds.; John Wiley and Sons: Chichester, 1998; pp 615–636.

[20] Bartlett R. J. (2010). The coupled-cluster
revolution. Mol. Phys..

[21] Crawford, T. D. ; Schaefer, H. F. In Reviews in Computational Chemistry; Lipkowitz, K. B. , Boyd, D. B. , Eds.; VCH Publishers: New York, 2000; Vol. 14; Chapter 2, pp 33–136.

[22] Dutta A. K., Neese F., Izsák R. (2016). Towards a
pair natural orbital coupled
cluster method for excited states. J. Chem.
Phys..

[23] Liakos D. G., Guo Y., Neese F. (2020). Comprehensive Benchmark Results for the Domain Based
Local Pair Natural Orbital Coupled Cluster Method (DLPNO-CCSD­(T))
for Closed- and Open-Shell Systems. J. Chem.
Phys. A.

[24] Riplinger C., Neese F. (2013). An efficient and near
linear scaling pair natural orbital based local
coupled cluster method. J. Chem. Phys..

[25] Riplinger C., Pinski P., Becker U., Valeev E. F., Neese F. (2016). Sparse maps—A
systematic infrastructure for reduced-scaling electronic structure
methods. II. Linear scaling domain based pair natural orbital coupled
cluster theory. J. Chem. Phys..

[26] Guo Y., Riplinger C., Becker U., Liakos D. G., Minenkov Y., Cavallo L., Neese F. (2018). Communication: An improved linear
scaling perturbative triples correction for the domain based local
pair-natural orbital based singles and doubles coupled cluster method
[DLPNO-CCSD­(T)]. J. Chem. Phys..

[27] Bhattacharjee S., Isegawa M., Garcia-Ratés M., Neese F., Pantazis D. A. (2022). Ionization
Energies and Redox Potentials of Hydrated Transition Metal Ions: Evaluation
of Domain-Based Local Pair Natural Orbital Coupled Cluster Approaches. J. Chem. Theory Comput..

[28] Altun A., Riplinger C., Neese F., Bistoni G. (2023). Exploring the Accuracy
Limits of PNO-Based Local Coupled-Cluster Calculations for Transition-Metal
Complexes. J. Chem. Theory Comput..

[29] Naim C., Besalú-Sala P., Zaleśny R., Luis J. M., Castet F., Matito E. (2023). Are Accelerated and
Enhanced Wave Function Methods
Accurate to Compute Static Linear and Nonlinear Optical Properties?. J. Chem. Theory Comput..

[30] Nagy P. R., Samu G., Kállay M. (2018). Optimization of the Linear-Scaling
Local Natural Orbital CCSD­(T) Method: Improved Algorithm and Benchmark
Applications. J. Chem. Theory Comput..

[31] Pipek J., Mezey P. G. (1989). A fast intrinsic localization procedure
applicable
for *ab initio* and semiempirical linear combination
of atomic orbital wave functions. J. Chem. Phys..

[32] Boys S. F. (1960). Construction
of Some Molecular Orbitals to Be Approximately Invariant for Changes
from One Molecule to Another. Rev. Mod. Phys..

[33] D’Cunha R., Crawford T. D. (2021). PNO++: Perturbed
Pair Natural Orbitals for Coupled
Cluster Linear Response Theory. J. Chem. Theory
Comput..

[34] D’Cunha R., Crawford T. D. (2023). Applications of
a perturbation-aware local correlation
method to coupled cluster linear response properties. Mol. Phys..

[35] Datta D., Kossmann S., Neese F. (2016). Analytic energy derivatives for the
calculation of the first-order molecular properties using the domain-based
local pair-natural orbital coupled-cluster theory. J. Chem. Phys..

[36] Dunning T. H. (1989). Gaussian
basis sets for use in correlated molecular calculations. I. The atoms
boron through neon. J. Chem. Phys..

[37] Kendall R. A., Dunning T. H., Harrison R. J. (1992). Electron effinities of the first-row
atoms revisited. Systematic basis sets and wave functions. J. Chem. Phys..

[38] Woon D. E., Dunning T. H. (1994). Gaussian basis sets
for use in correlated molecular
calculations. IV. Calculation of static electrical response properties. J. Chem. Phys..

[39] Crawford, T. D. ; Peyton, B. G. ; Wang, Z. ; Madriaga, J. M. http://github.com/CrawfordGroup/pycc (accessed on 20 September, 2025).

[40] Smith D. G., Burns L. A., Simmonett A. C., Parrish R. M., Schieber M. C., Galvelis R., Kraus P., Kruse H., Di Remigio R., Alenaizan A. (2020). PSI4 1.4: Open-source software for high-throughput
quantum chemistry. J. Chem. Phys..

[41] Shelton D. P., Rice J. E. (1994). Measurements and Calculations of the Hyperpolarkabilities
of Atoms and Small Molecules in the Gas Phase. Chem. Rev..

[42] Howard J. C., Sowndarya S. V., Ansari I. M., Mach T. J., Baranowska-Łączkowska A., Crawford T. D. (2018). On the Performance of Property-Optimized Basis Sets
for Optical Rotation With Coupled Cluster Theory. J. Phys. Chem. A.

[43] Shumberger B. M., Fink E. H., King R. A., Crawford T. D. (2023). On the use of property-oriented
basis sets for the simulation of vibrational chiroptical spectroscopies. Mol. Phys..

[44] Björck Å., Golub G. H. (1973). Numerical Methods for Computing Angles Between Linear
Subspaces. Math. Comput..

[45] Russ N. J., Crawford T. D. (2004). Potential energy surface discontinuities in local correlation
methods. J. Chem. Phys..

