# Supplementary material for: Occurrence and Impact of Electric-Field-Induced Discontinuities in Correlation Energies from Localized Pair-Natural-Orbital Methods
Source: J Phys Chem A. 2025 Oct 16;129(43):10014–30. doi: 10.1021/acs.jpca.5c05210 (PMC12581137; doi:10.1021/acs.jpca.5c05210)
Supplement: Supplementary file 1 [file jp5c05210_si_001.pdf]

Supporting Information: On the Occurrence and Impact of Electric-Field-Induced Discontinuities in Correlation Energies from Localized Pair-Natural-Orbital Methods

Jose P. Madriaga and T. Daniel Crawford*

Department of Chemistry, Virginia Tech, Blacksburg, VA 24061, USA

E-mail: crawdad@vt.edu

Contents

1 Molecular Geometries **S6**

1.1 H_2O S6

1.2 HOF S6

1.3 Fluoroethylene S6

1.4 Cis-Butadiene S6

2 Correlation Energies **S7**

2.1 Water/cc-pVDZ S7

2.2 PNO-Relaxed Water/cc-pVDZ S8

2.3 Water/aug-cc-pVDZ S9

2.4 Frozen Core Water/cc-pVDZ S12
2.5 Frozen Core Water/aug-cc-pVDZ S13
2.6 HOF/cc-pVDZ S14
2.7 HOF/aug-cc-pVDZ S15
2.8 Fluoroethylene/cc-pVDZ S16
2.9 Fluoroethylene/aug-cc-pVDZ S17
2.10 Butadiene/cc-pVDZ S18
2.11 Butadiene/aug-cc-pVDZ S19

3 Correlation Contribution to Electric Dipole Moments S20

3.1 Water/cc-pVDZ S20
3.2 PNO-Relaxed Water/cc-pVDZ S21
3.3 Water/aug-cc-pVDZ S22
3.4 Frozen Core Water/cc-pVDZ S25
3.5 Frozen Core Water/aug-cc-pVDZ S26
3.6 HOF/cc-pVDZ S27
3.7 HOF/aug-cc-pVDZ S28
3.8 Fluoroethylene/cc-pVDZ S29
3.9 Fluoroethylene/aug-cc-pVDZ S30
3.10 Butadiene/cc-pVDZ S31
3.11 Butadiene/aug-cc-pVDZ S32

4 Correlation Contribution to Electric Polarizabilities S33

4.1 Water/cc-pVDZ S33
4.2 PNO-Relaxed Water/cc-pVDZ S34
4.3 Water/aug-cc-pVDZ S35
4.4 Frozen Core Water/cc-pVDZ S38
4.5 Frozen Core Water/aug-cc-pVDZ S39

4.6 HOF/cc-pVDZ . . . S40
4.7 HOF/aug-cc-pVDZ . . . S41
4.8 Fluoroethylene/cc-pVDZ . . . S42
4.9 Fluoroethylene/aug-cc-pVDZ . . . S43
4.10 Butadiene/cc-pVDZ . . . S44
4.11 Butadiene/aug-cc-pVDZ . . . S45

5 Correlation Contribution to Electric Hyperpolarizabilities S46

5.1 Water/cc-pVDZ . . . S46
5.2 PNO-Relaxed Water/cc-pVDZ . . . S47
5.3 Water/aug-cc-pVDZ . . . S48
5.4 Frozen Core Water/cc-pVDZ . . . S51
5.5 Frozen Core Water/aug-cc-pVDZ . . . S52
5.6 HOF/cc-pVDZ . . . S53
5.7 HOF/aug-cc-pVDZ . . . S54
5.8 Fluoroethylene/cc-pVDZ . . . S55
5.9 Fluoroethylene/aug-cc-pVDZ . . . S56
5.10 Butadiene/cc-pVDZ . . . S57
5.11 Butadiene/aug-cc-pVDZ . . . S58

6 Total Energies S59

6.1 Water/cc-pVDZ . . . S59
6.2 PNO-Relaxed Water/cc-pVDZ . . . S60
6.3 Water/aug-cc-pVDZ . . . S61
6.4 Frozen Core Water/cc-pVDZ . . . S64
6.5 Frozen Core Water/aug-cc-pVDZ . . . S65
6.6 HOF/cc-pVDZ . . . S66
6.7 HOF/aug-cc-pVDZ . . . S67

6.8 Fluoroethylene/cc-pVDZ S68
6.9 Fluoroethylene/aug-cc-pVDZ S69
6.10 Butadiene/cc-pVDZ S70
6.11 Butadiene/aug-cc-pVDZ S71

7 Total Contribution to to Electric Dipole Moments S72

7.1 Water/cc-pVDZ S72
7.2 PNO-Relaxed Water/cc-pVDZ S73
7.3 Water/aug-cc-pVDZ S74
7.4 Frozen Core Water/cc-pVDZ S77
7.5 Frozen Core Water/aug-cc-pVDZ S78
7.6 HOF/cc-pVDZ S79
7.7 HOF/aug-cc-pVDZ S80
7.8 Fluoroethylene/cc-pVDZ S81
7.9 Fluoroethylene/aug-cc-pVDZ S82
7.10 Butadiene/cc-pVDZ S83
7.11 Butadiene/aug-cc-pVDZ S84

8 Total Contribution to to Electric Polarizability S85

8.1 Water/cc-pVDZ S85
8.2 PNO-Relaxed Water/cc-pVDZ S86
8.3 Water/aug-cc-pVDZ S87
8.4 Frozen Core Water/cc-pVDZ S90
8.5 Frozen Core Water/aug-cc-pVDZ S91
8.6 HOF/cc-pVDZ S92
8.7 HOF/aug-cc-pVDZ S93
8.8 Fluoroethylene/cc-pVDZ S94
8.9 Fluoroethylene/aug-cc-pVDZ S95

8.10 Butadiene/cc-pVDZ S96

8.11 Butadiene/aug-cc-pVDZ S97

9 Total Contribution to to Electric Hyperpolarizability S98

9.1 Water/cc-pVDZ S98

9.2 PNO-Relaxed Water/cc-pVDZ S99

9.3 Water/aug-cc-pVDZ S100

9.4 Frozen Core Water/cc-pVDZ S103

9.5 Frozen Core Water/aug-cc-pVDZ S104

9.6 HOF/cc-pVDZ S105

9.7 HOF/aug-cc-pVDZ S106

9.8 Fluoroethylene/cc-pVDZ S107

9.9 Fluoroethylene/aug-cc-pVDZ S108

9.10 Butadiene/cc-pVDZ S109

9.11 Butadiene/aug-cc-pVDZ S110

1 Molecular Geometries

Cartesian coordinates are given in a_0.

1.1 H_2O

```
O 0.000000000000 0.000000000000 0.143225816552
H 1.638036840407 0.000000000000 -1.136548822547
H -1.638036840407 0.000000000000 -1.136548822547
```

1.2 HOF

```
O 0.00000000 0.00000000 0.00000000
F 0.00000000 0.00000000 2.59991544
H -1.77281255 0.00000000 -0.30620555
```

1.3 Fluoroethylene

```
C   0.0000000 0.0000000 -0.8216246
C   2.2401815 0.0000000 0.2910518
F   -2.1602914 0.0000000 0.5080888
H   -0.3312595 0.0000000 -2.8332683
H   2.4250062 0.0000000 2.3204665
H   3.9077892 0.0000000 -0.8765703
```

1.4 Cis-Butadiene

```
C 0.2388349 1.3915282 1.0478834
C -0.2388349 -1.3915282 1.0478834
C -0.2388349 2.9219569 -0.9253082
C 0.2388349 -2.9219569 -0.9253082
H 1.0092234 2.1832800 2.7925107
H -1.0092234 -2.1832800 2.7925107
H 0.1695387 4.9380037 -0.8611123
H -1.0780245 2.2100895 -2.6668533
H -0.1695387 -4.9380037 -0.8611123
H 1.0780245 -2.2100895 -2.6668533
```

2 Correlation Energies

2.1 Water/cc-pVDZ

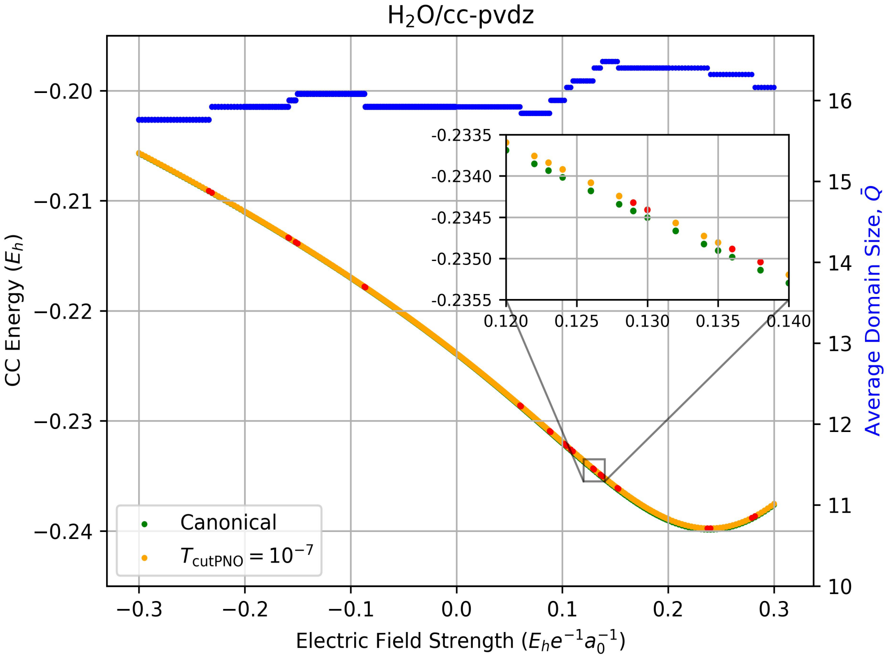

Figure S1: Correlation energies for water cc-pVDZ with a $T_{\mathrm{cutPNO}} = 10^{-7}$ as a function of external electric field strength.

2.2 PNO-Relaxed Water/cc-pVDZ

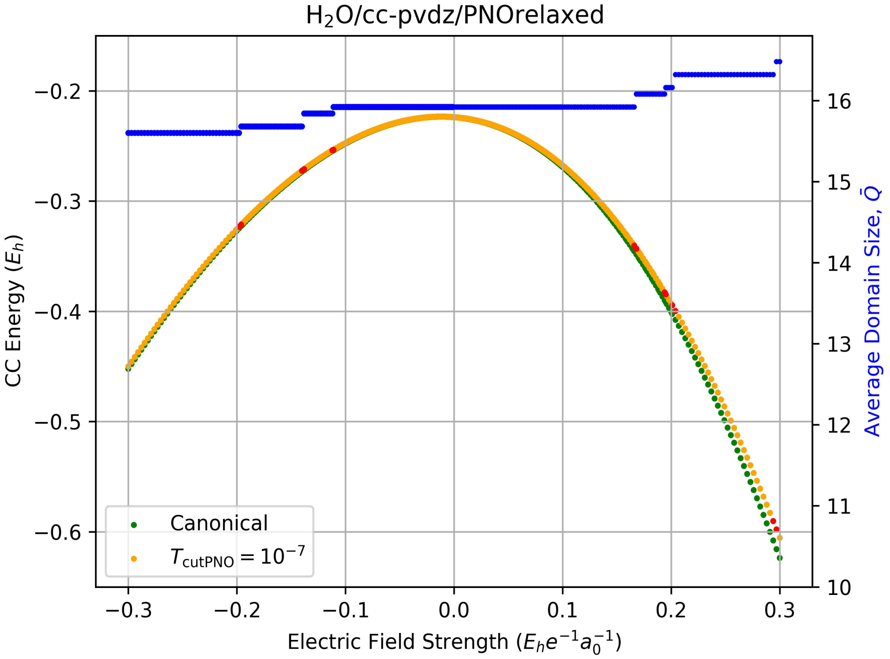

Figure S2: Correlation energies for PNO-relaxed water cc-pVDZ with a $T_{\mathrm{cutPNO}} = 10^{-7}$ as a function of external electric field strength.

2.3 Water/aug-cc-pVDZ

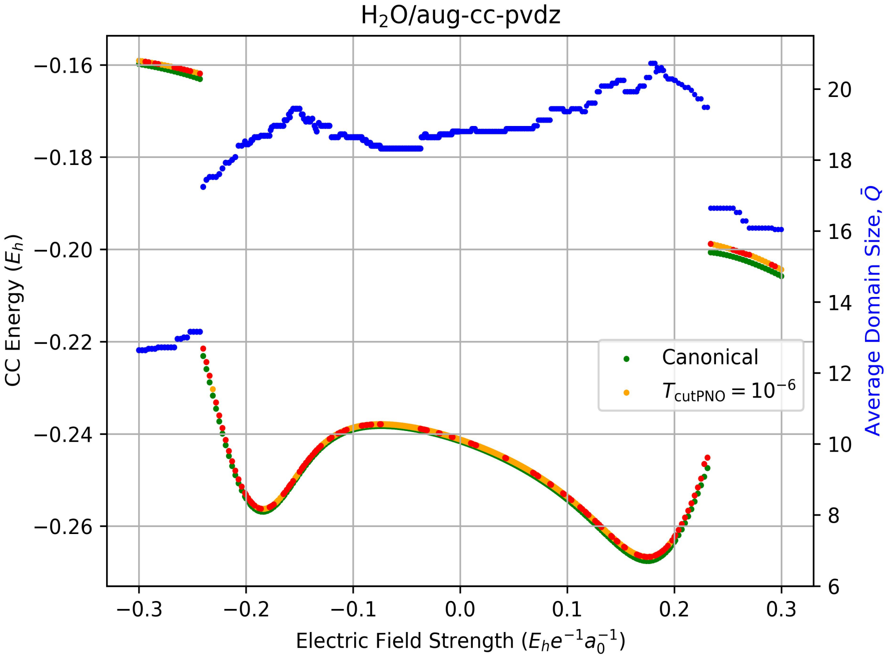

Figure S3: Correlation energies for water aug-cc-pVDZ with a $T_{\text{cutPNO}} = 10^{-6}$ as a function of external electric field strength.

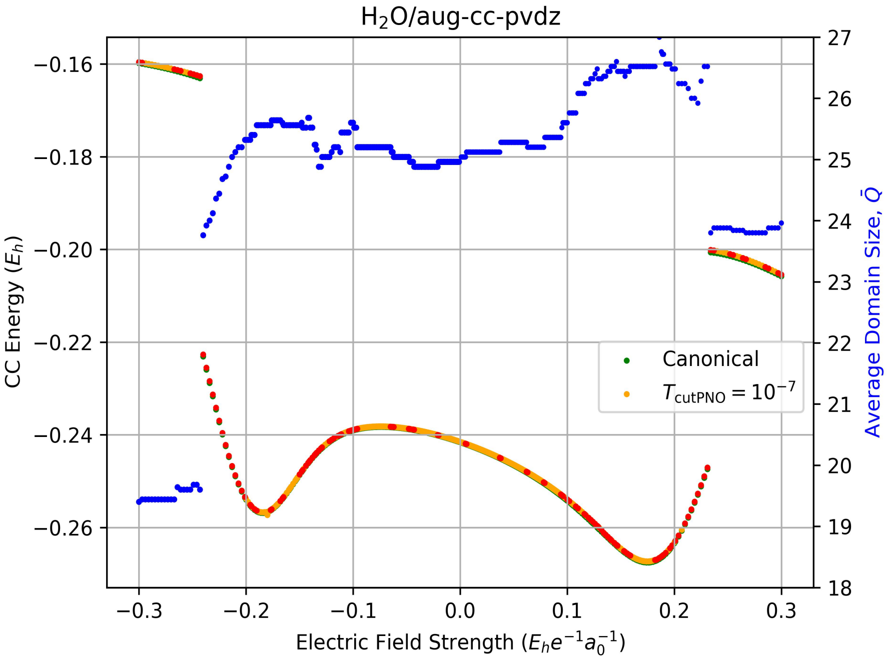

Figure S4: Correlation energies for water aug-cc-pVDZ with a $T_{\text{cutPNO}} = 10^{-7}$ as a function of external electric field strength.

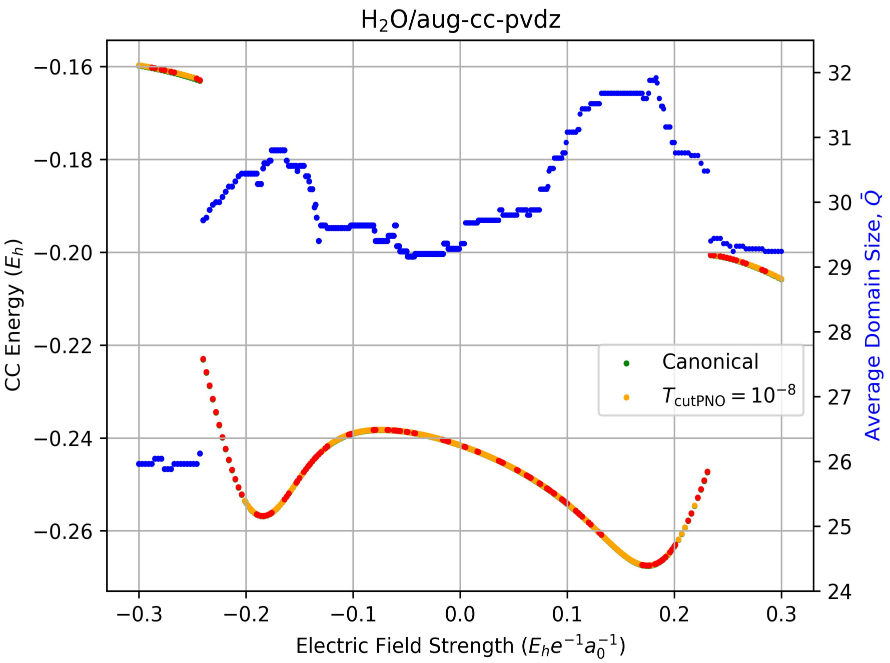

Figure S5: Correlation energies for water aug-cc-pVDZ with a $T_{\mathrm{cutPNO}} = 10^{-8}$ as a function of external electric field strength.

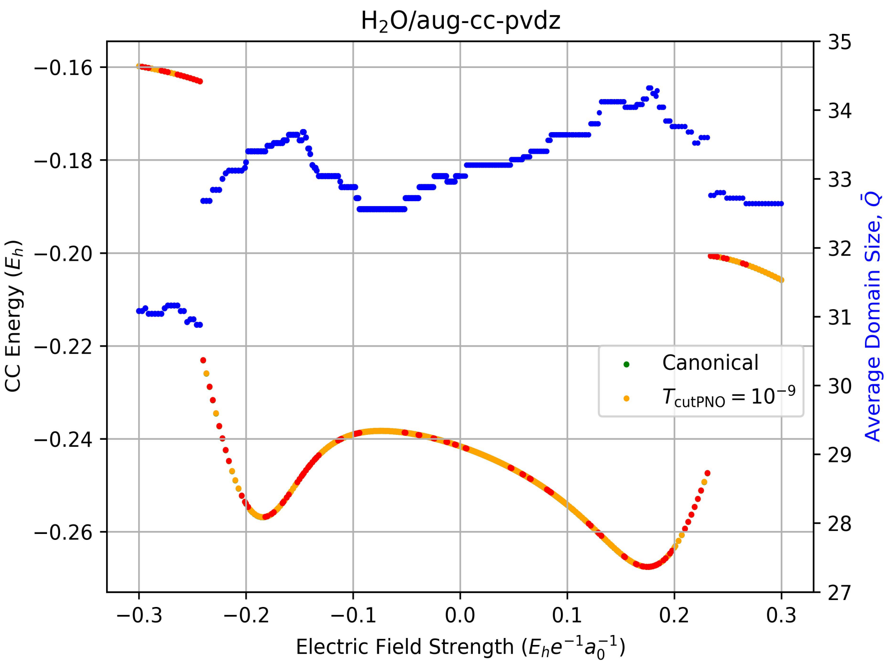

Figure S6: Correlation energies for water aug-cc-pVDZ with a $T_{\mathrm{cutPNO}} = 10^{-9}$ as a function of external electric field strength.

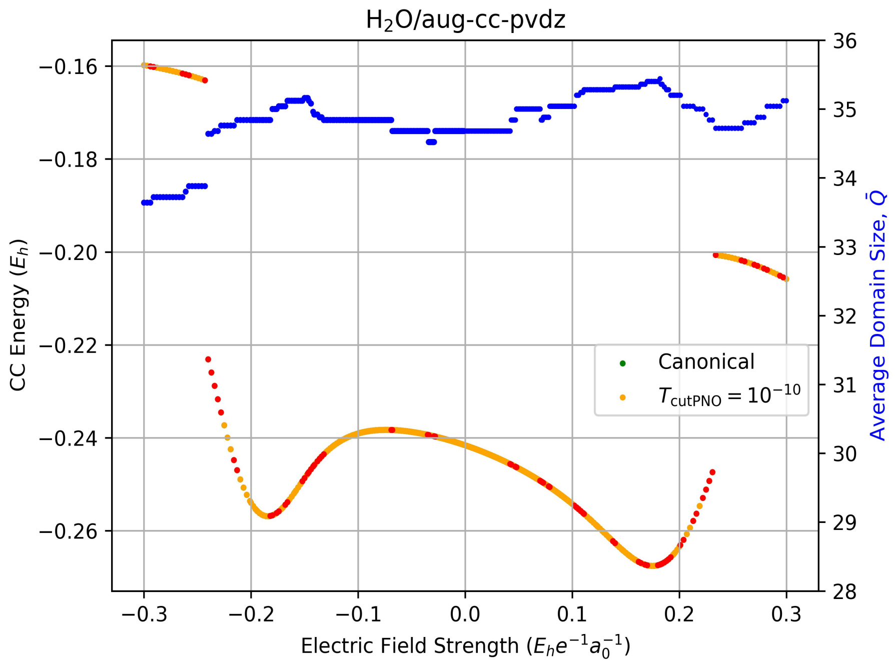

Figure S7: Correlation energies for water aug-cc-pVDZ with a $T_{\text{cutPNO}} = 10^{-10}$ as a function of external electric field strength.

2.4 Frozen Core Water/cc-pVDZ

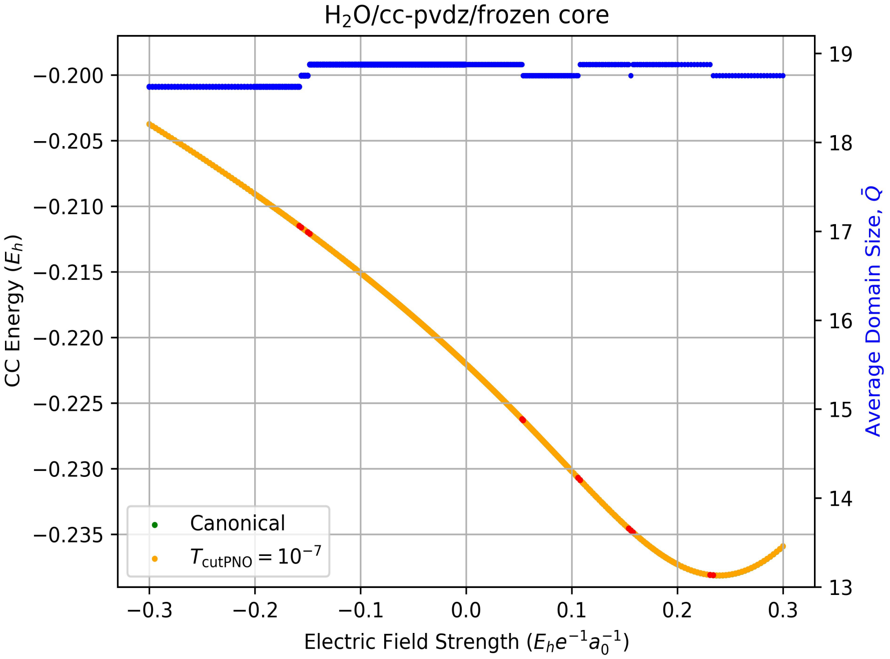

Figure S8: Correlation energies for frozen core water cc-pVDZ with a $T_{\text{cutPNO}} = 10^{-7}$ as a function of external electric field strength.

2.5 Frozen Core Water/aug-cc-pVDZ

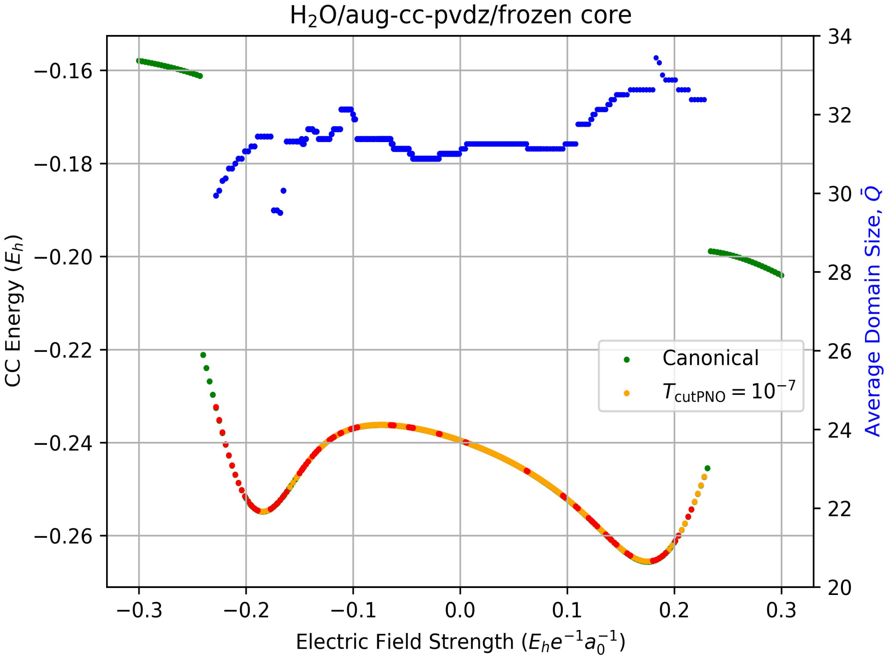

Figure S9: Correlation energies for frozen core water aug-cc-pVDZ with a $T_{\text{cutPNO}} = 10^{-7}$ as a function of external electric field strength.

2.6 HOF/cc-pVDZ

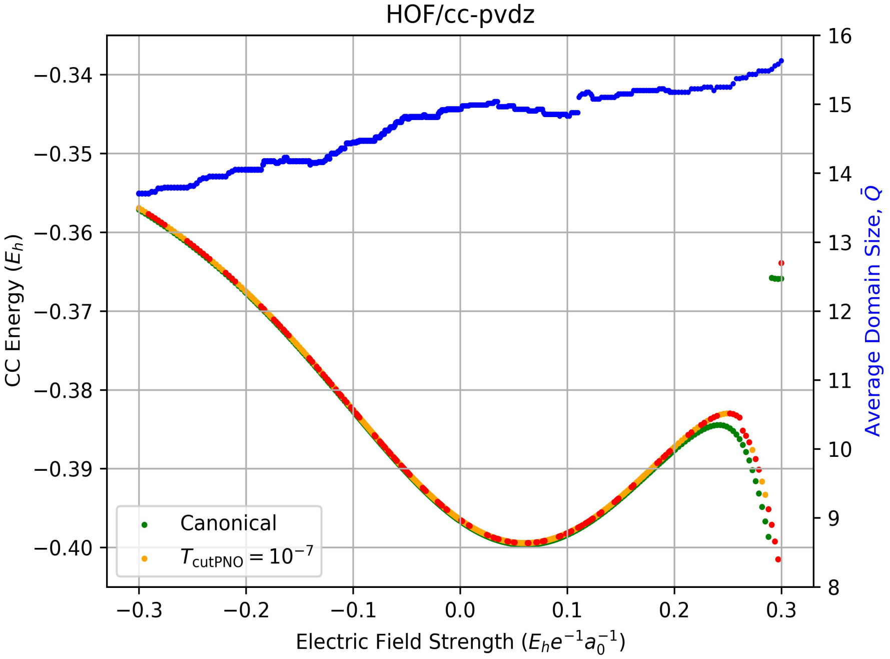

Figure S10: Correlation energies for HOF cc-pVDZ with a $T_{\text{cutPNO}} = 10^{-7}$ as a function of external electric field strength.

2.7 HOF/aug-cc-pVDZ

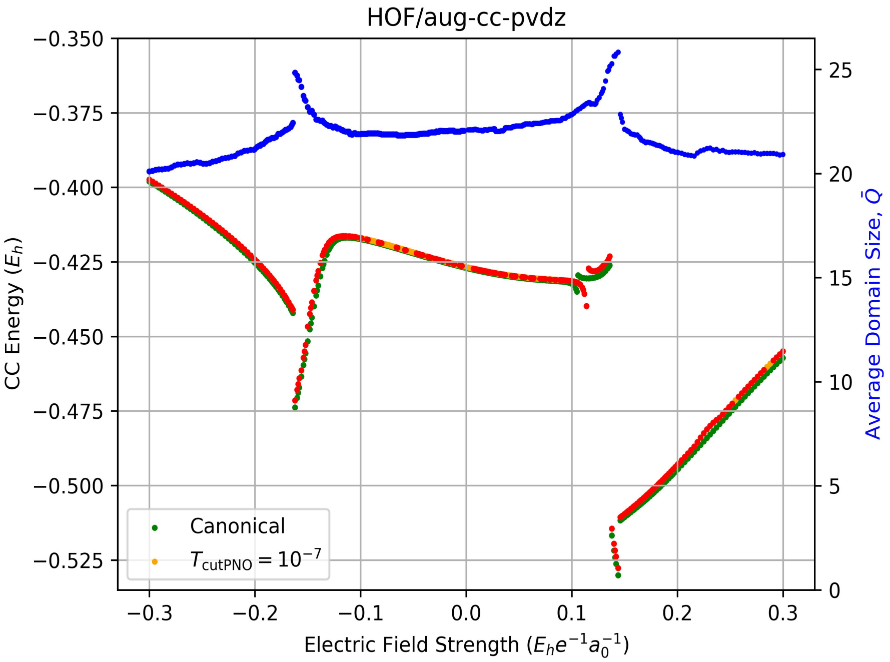

Figure S11: Correlation energies for HOF aug-cc-pVDZ with a $T_{\mathrm{cutPNO}} = 10^{-7}$ as a function of external electric field strength.

2.8 Fluoroethylene/cc-pVDZ

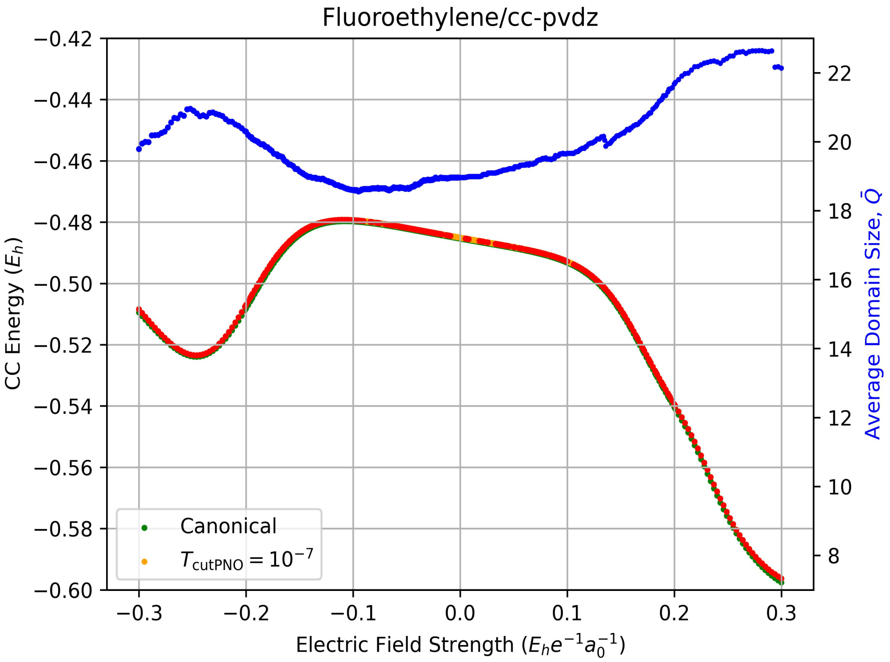

Figure S12: Correlation energies for fluoroethylene cc-pVDZ with a $T_{\text{cutPNO}} = 10^{-7}$ as a function of external electric field strength.

2.9 Fluoroethylene/aug-cc-pVDZ

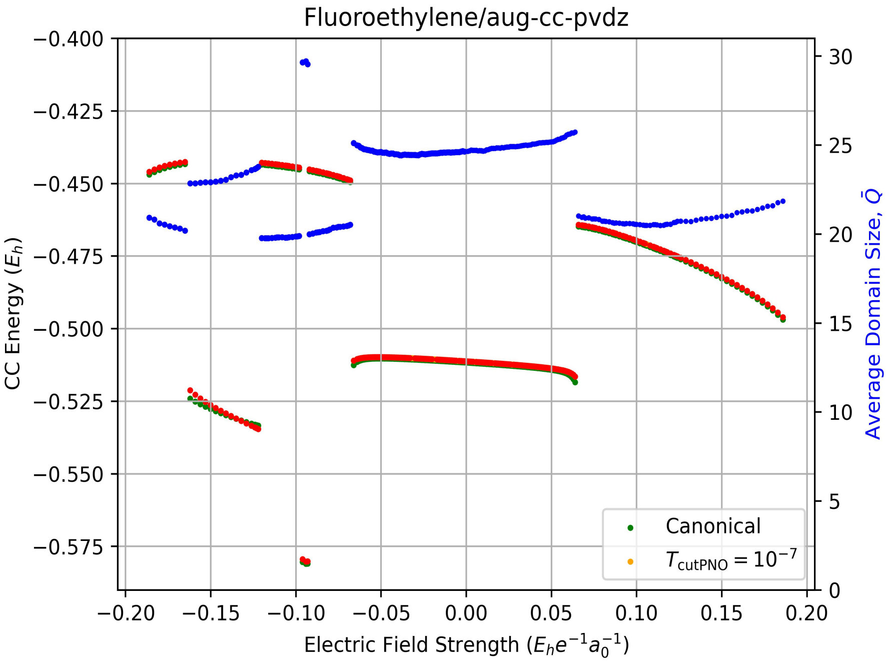

Figure S13: Correlation energies for fluoroethylene aug-cc-pVDZ with a $T_{\text{cutPNO}} = 10^{-7}$ as a function of external electric field strength.

2.10 Butadiene/cc-pVDZ

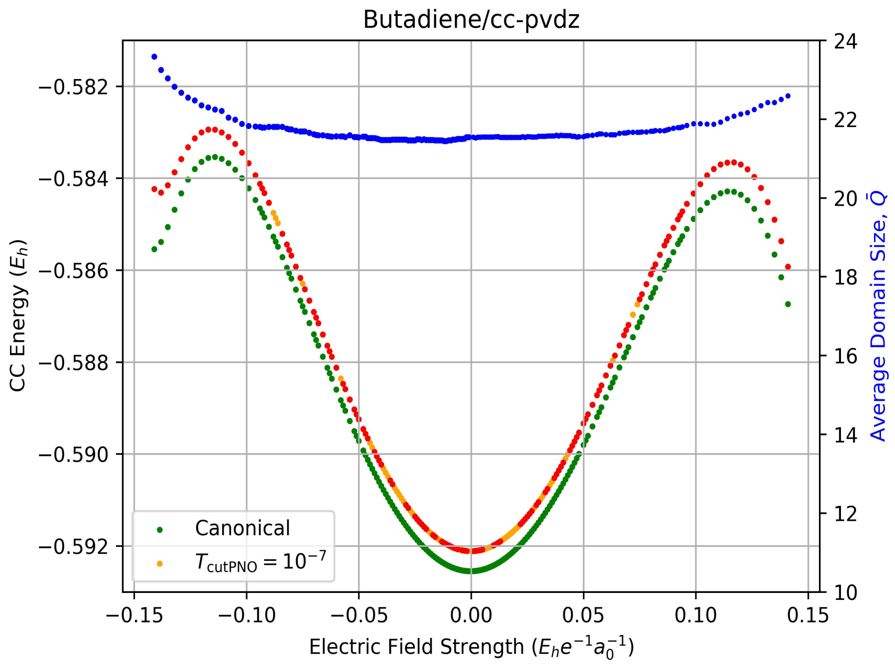

Figure S14: Correlation energies for butadiene cc-pVDZ with a $T_{\text{cutPNO}} = 10^{-7}$ as a function of external electric field strength.

2.11 Butadiene/aug-cc-pVDZ

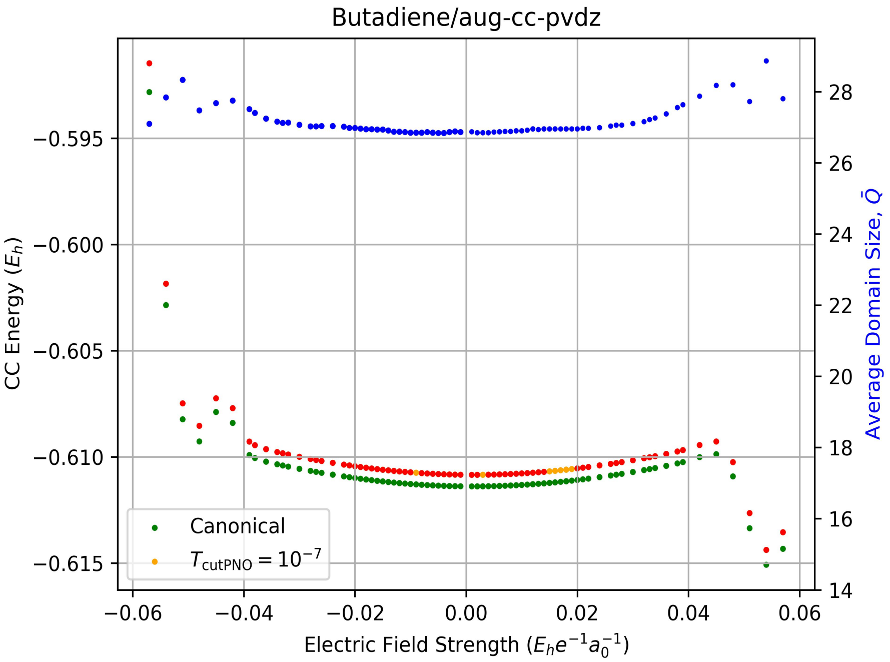

Figure S15: Correlation energies for butadiene aug-cc-pVDZ with a $T_{\text{cutPNO}} = 10^{-7}$ as a function of external electric field strength.

3 Correlation Contribution to Electric Dipole Moments

3.1 Water/cc-pVDZ

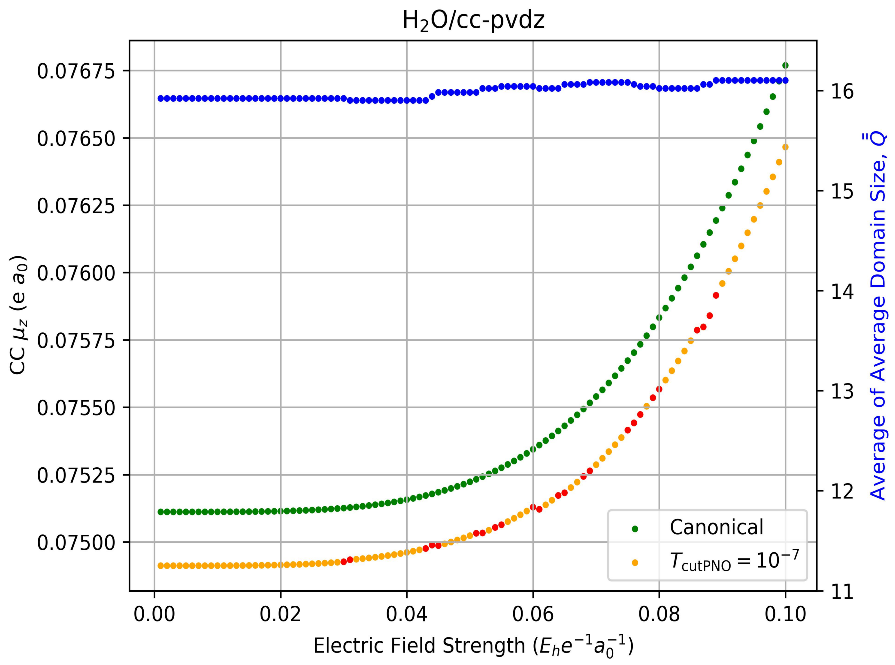

Figure S16: Correlation contribution to electric dipole moment for water cc-pVDZ with a $T_{\mathrm{cutPNO}} = 10^{-7}$ as a function of external electric field strength.

3.2 PNO-Relaxed Water/cc-pVDZ

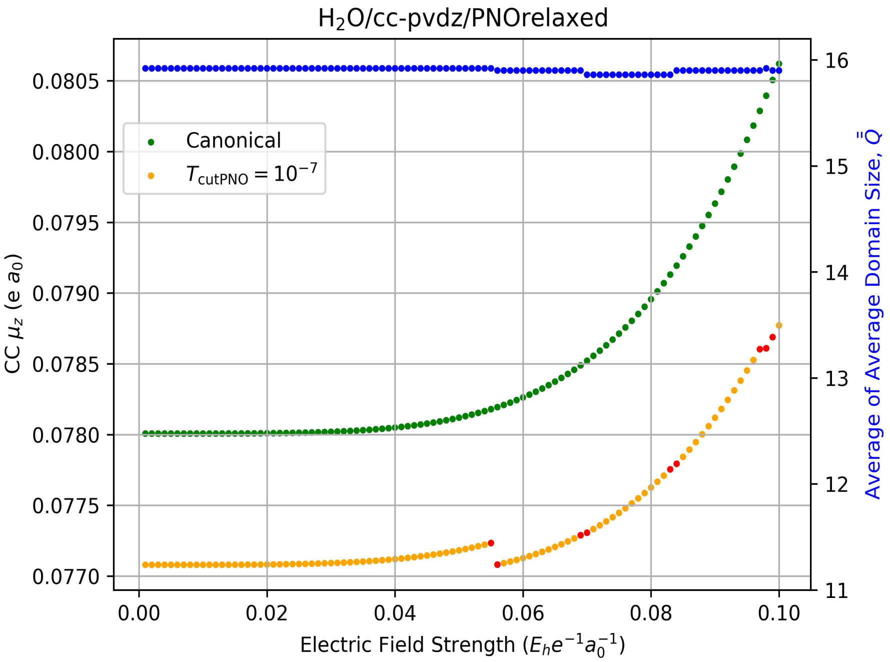

Figure S17: Correlation contribution to electric dipole moment for PNO-relaxed water cc-pVDZ with a $T_{\mathrm{cutPNO}} = 10^{-7}$ as a function of external electric field strength.

3.3 Water/aug-cc-pVDZ

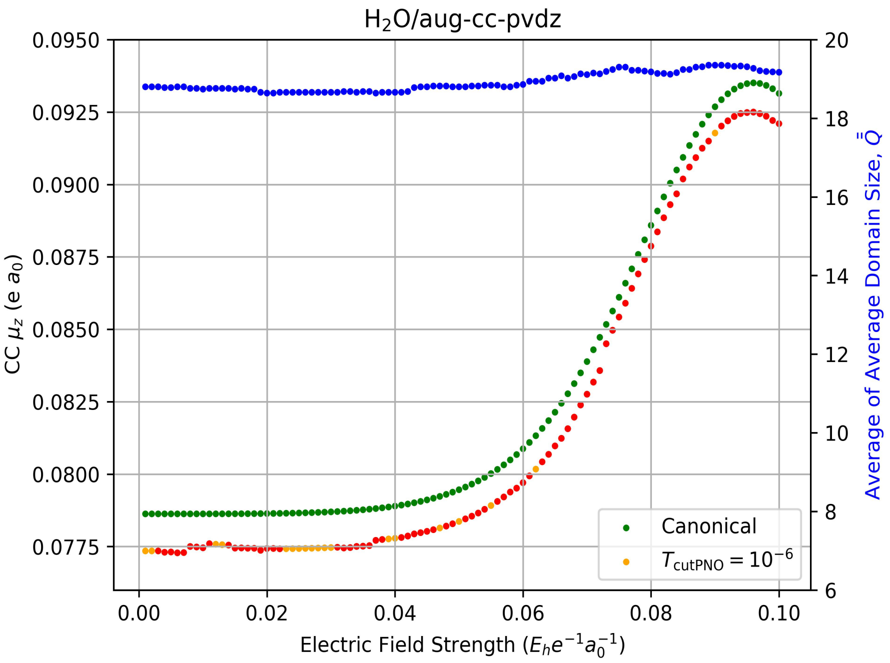

Figure S18: Correlation contribution to electric dipole moment for water aug-cc-pVDZ with a $T_{\mathrm{cutPNO}} = 10^{-6}$ as a function of external electric field strength.

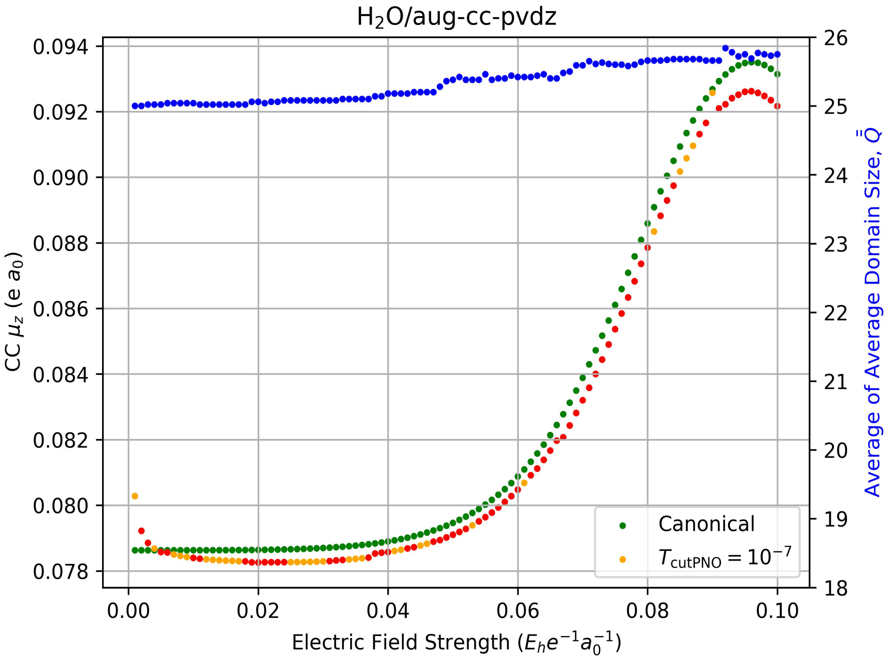

Figure S19: Correlation contribution to electric dipole moment for water aug-cc-pVDZ with a $T_{\mathrm{cutPNO}} = 10^{-7}$ as a function of external electric field strength.

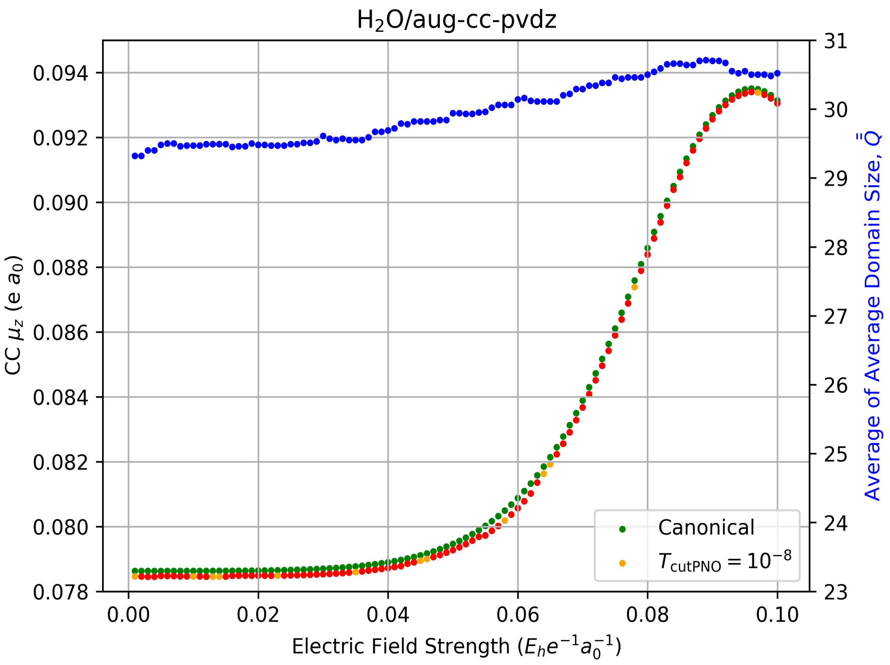

Figure S20: Correlation contribution to electric dipole moment for water aug-cc-pVDZ with a $T_{\mathrm{cutPNO}} = 10^{-8}$ as a function of external electric field strength.

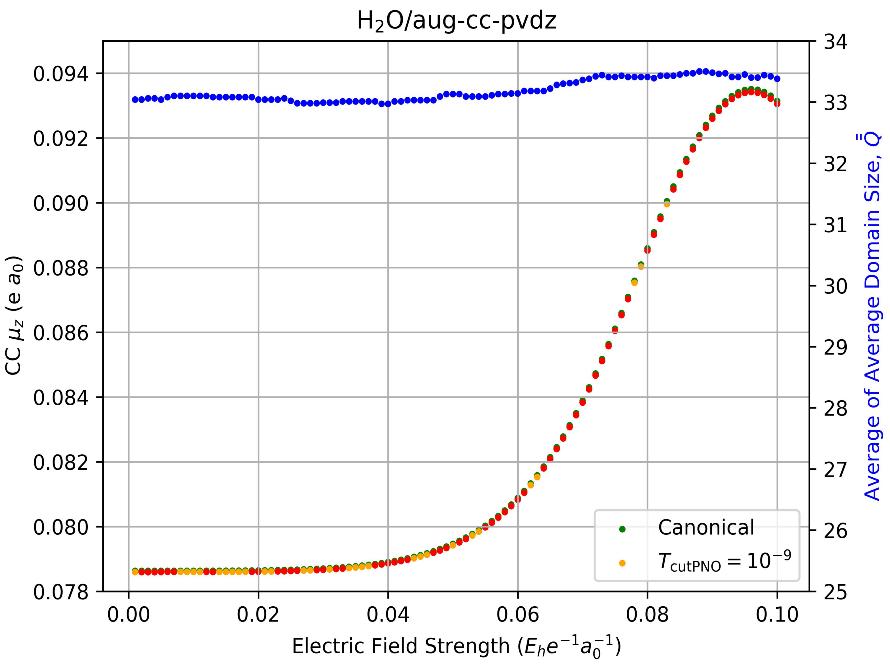

Figure S21: Correlation contribution to electric dipole moment for water aug-cc-pVDZ with a $T_{\mathrm{cutPNO}} = 10^{-9}$ as a function of external electric field strength.

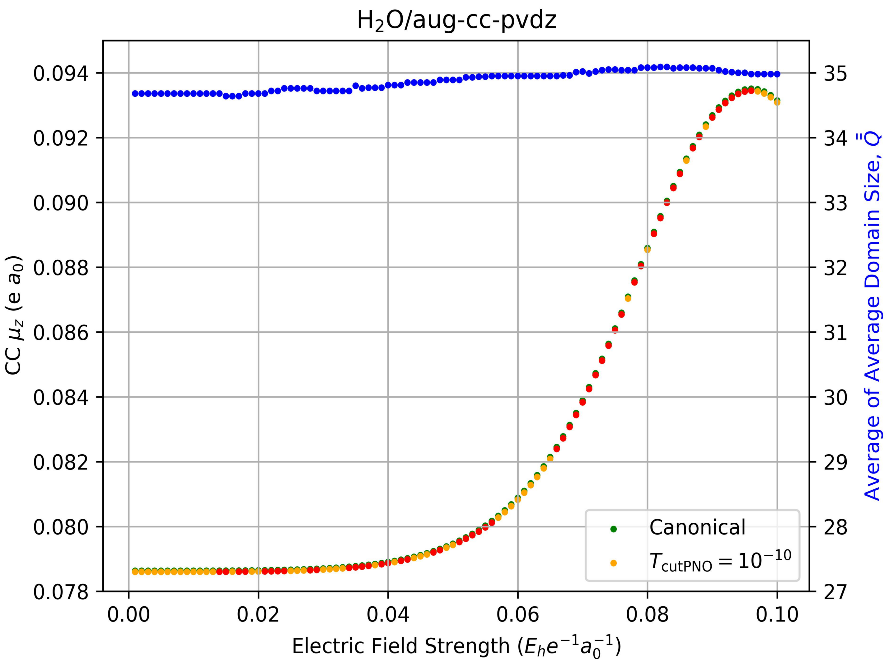

Figure S22: Correlation contribution to electric dipole moment for water aug-cc-pVDZ with a $T_{\text{cutPNO}} = 10^{-10}$ as a function of external electric field strength.

3.4 Frozen Core Water/cc-pVDZ

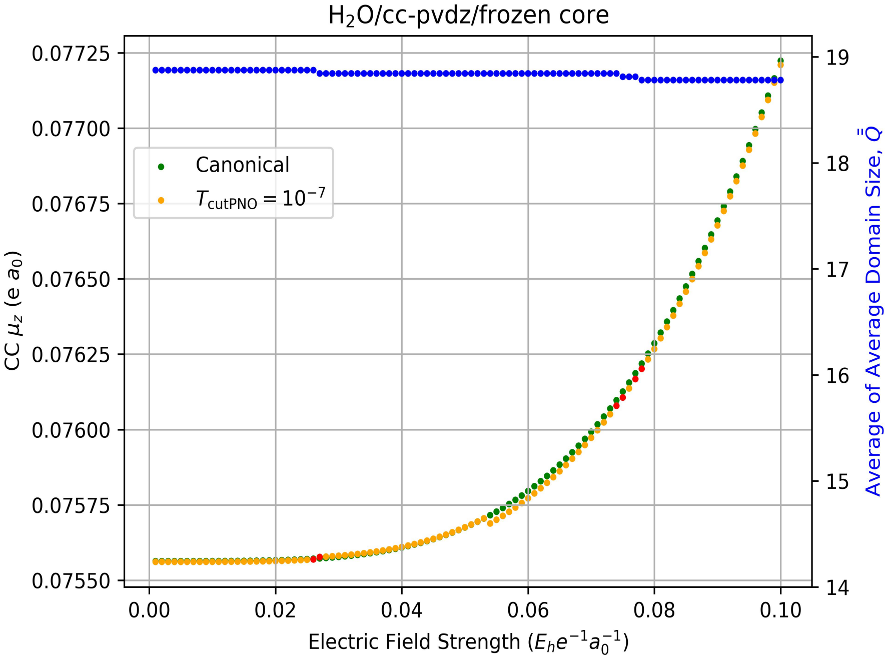

Figure S23: Correlation contribution to electric dipole moment for frozen core water cc-pVDZ with a $T_{\mathrm{cutPNO}} = 10^{-7}$ as a function of external electric field strength.

3.5 Frozen Core Water/aug-cc-pVDZ

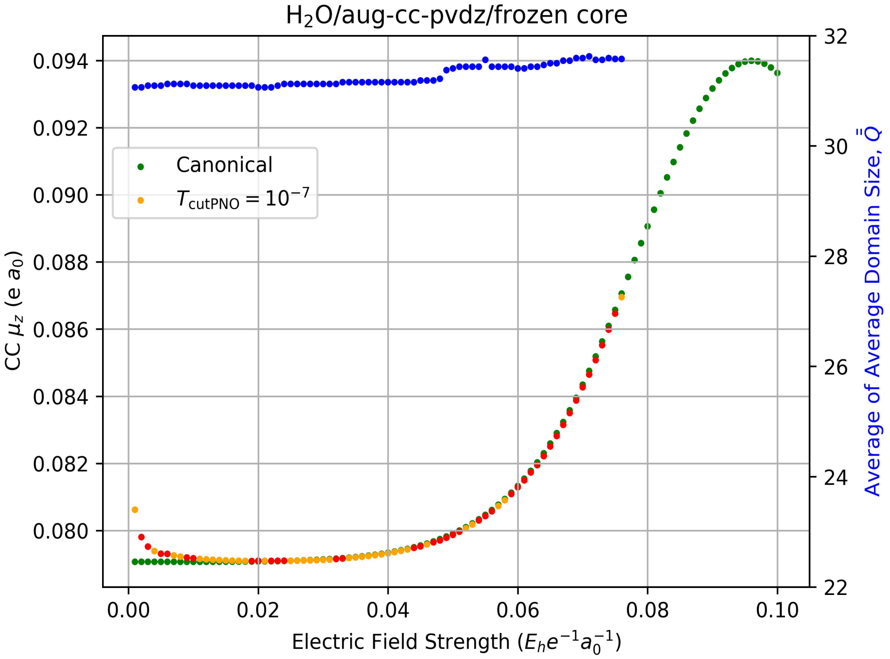

Figure S24: Correlation contribution to electric dipole moment for frozen core water aug-cc-pVDZ with a $T_{\mathrm{cutPNO}} = 10^{-7}$ as a function of external electric field strength.

3.6 HOF/cc-pVDZ

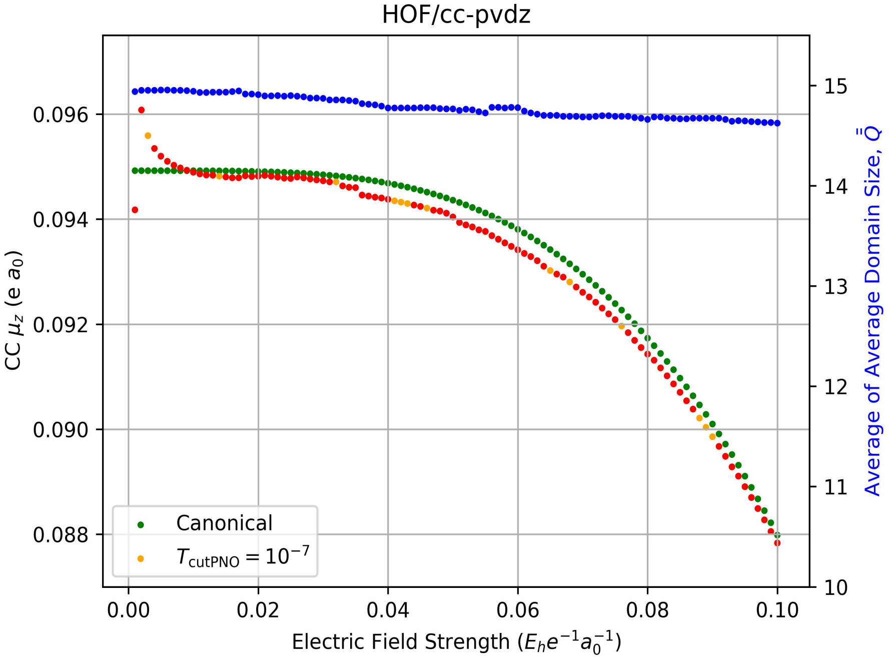

Figure S25: Correlation contribution to electric dipole moment for HOF cc-pVDZ with a $T_{\text{cutPNO}} = 10^{-7}$ as a function of external electric field strength.

3.7 HOF/aug-cc-pVDZ

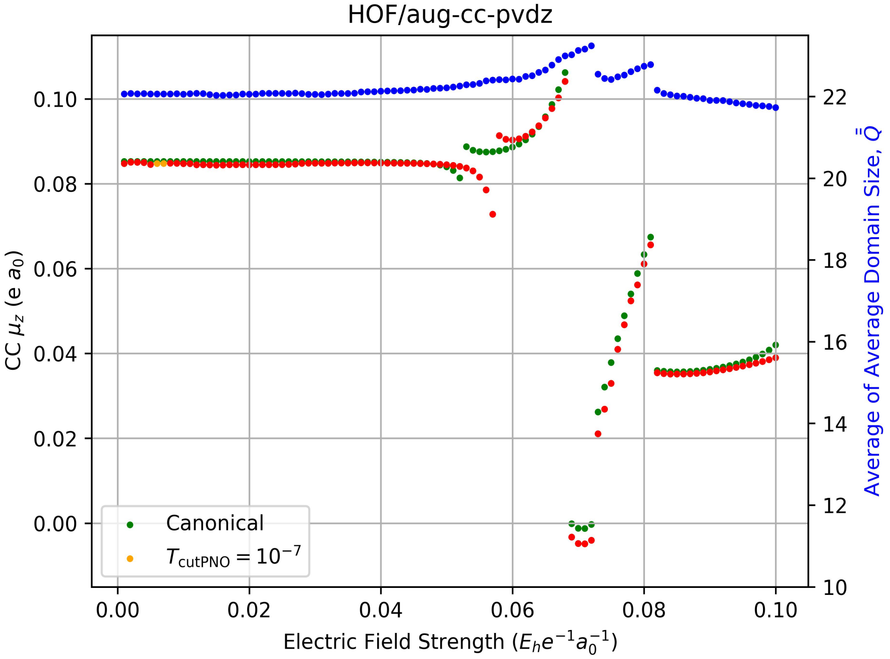

Figure S26: Correlation contribution to electric dipole moment for HOF aug-cc-pVDZ with a $T_{\text{cutPNO}} = 10^{-7}$ as a function of external electric field strength.

3.8 Fluoroethylene/cc-pVDZ

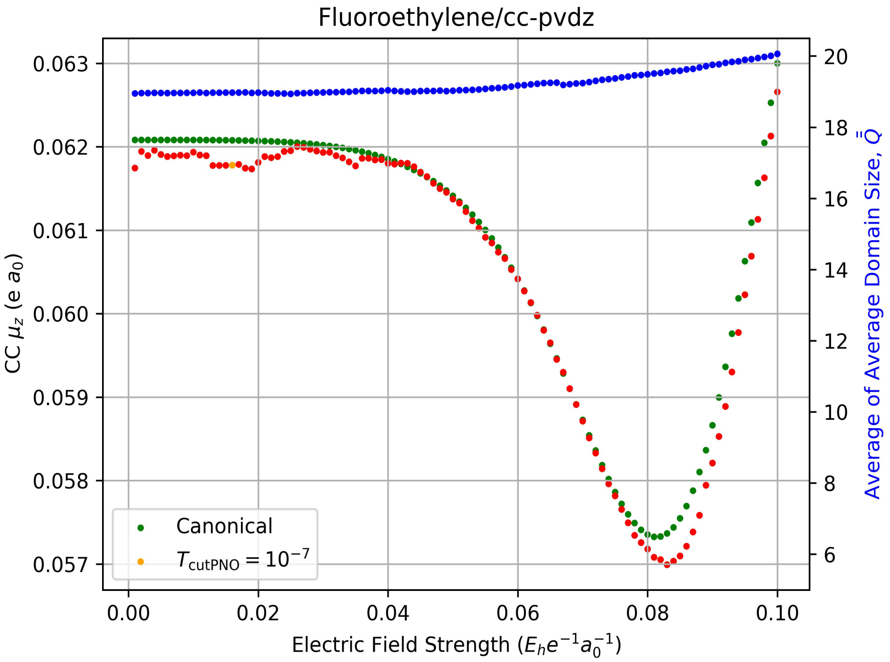

Figure S27: Correlation contribution to electric dipole moment for fluoroethylene cc-pVDZ with a $T_{\mathrm{cutPNO}} = 10^{-7}$ as a function of external electric field strength.

3.9 Fluoroethylene/aug-cc-pVDZ

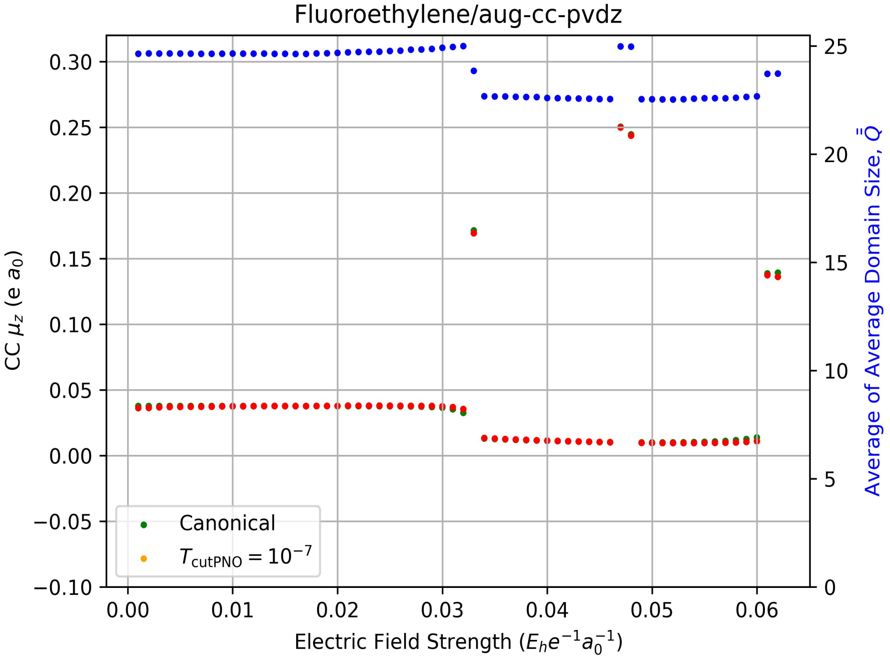

Figure S28: Correlation contribution to electric dipole moment for fluoroethylene aug-cc-pVDZ with a $T_{\mathrm{cutPNO}} = 10^{-7}$ as a function of external electric field strength.

3.10 Butadiene/cc-pVDZ

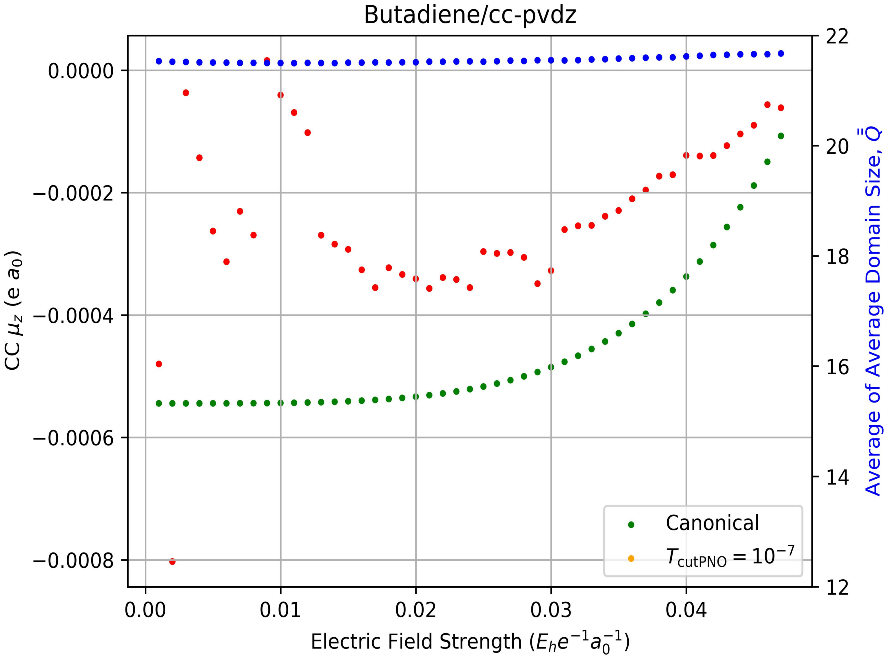

Figure S29: Correlation contribution to electric dipole moment for butadiene cc-pVDZ with a $T_{\mathrm{cutPNO}} = 10^{-7}$ as a function of external electric field strength.

3.11 Butadiene/aug-cc-pVDZ

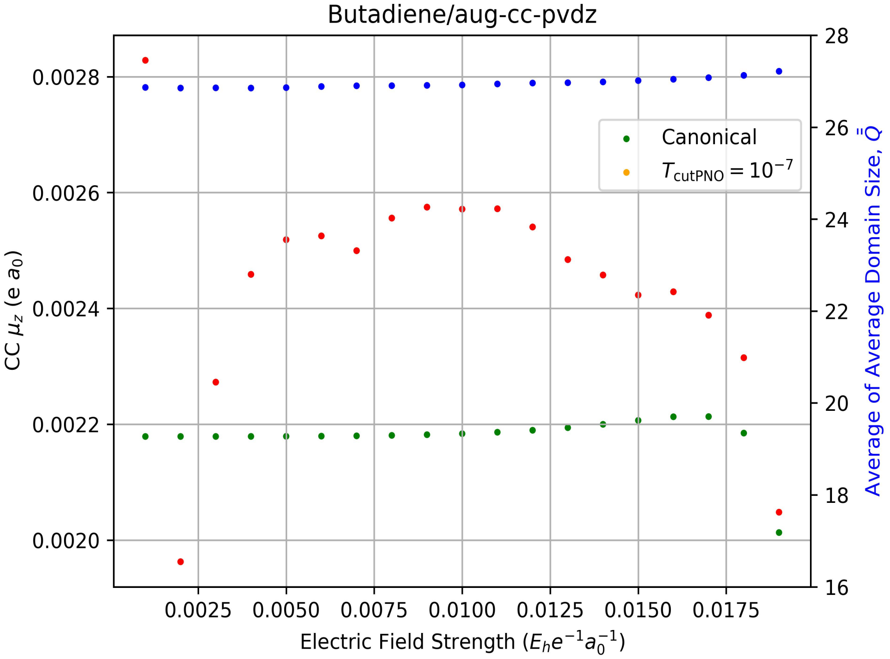

Figure S30: Correlation contribution to electric dipole moment for butadiene aug-cc-pVDZ with a $T_{\mathrm{cutPNO}} = 10^{-7}$ as a function of external electric field strength.

4 Correlation Contribution to Electric Polarizabilities

4.1 Water/cc-pVDZ

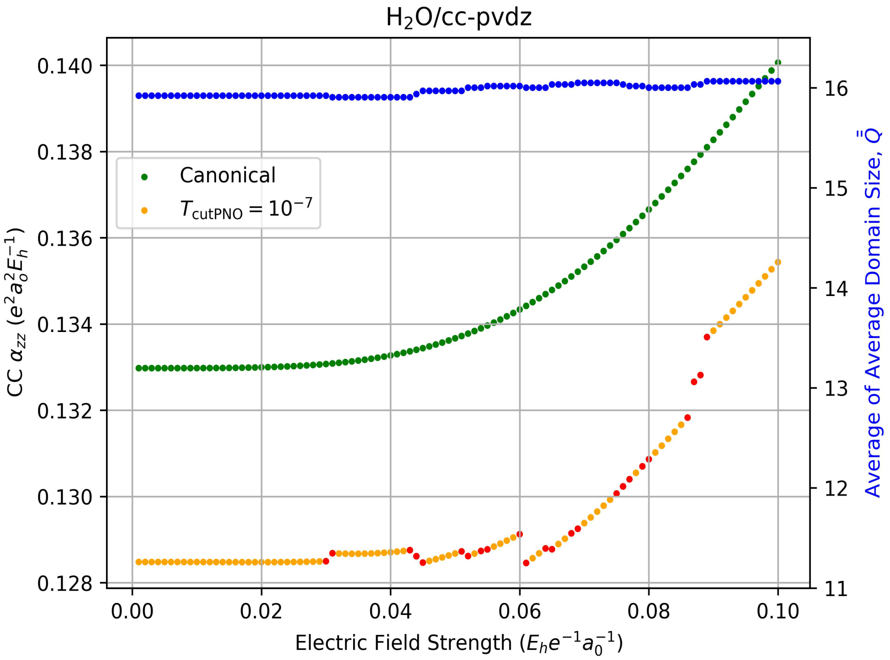

Figure S31: Correlation contribution to electric polarizability for water cc-pVDZ with a $T_{\text{cutPNO}} = 10^{-7}$ as a function of external electric field strength.

4.2 PNO-Relaxed Water/cc-pVDZ

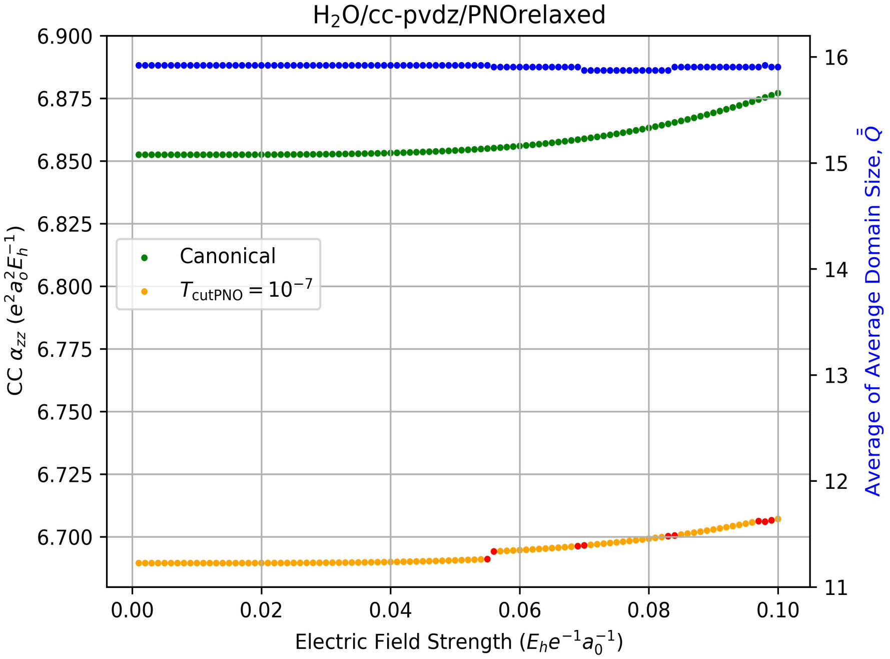

Figure S32: Correlation contribution to electric polarizability for water cc-pVDZ with a $T_{\mathrm{cutPNO}} = 10^{-7}$ as a function of external electric field strength.

4.3 Water/aug-cc-pVDZ

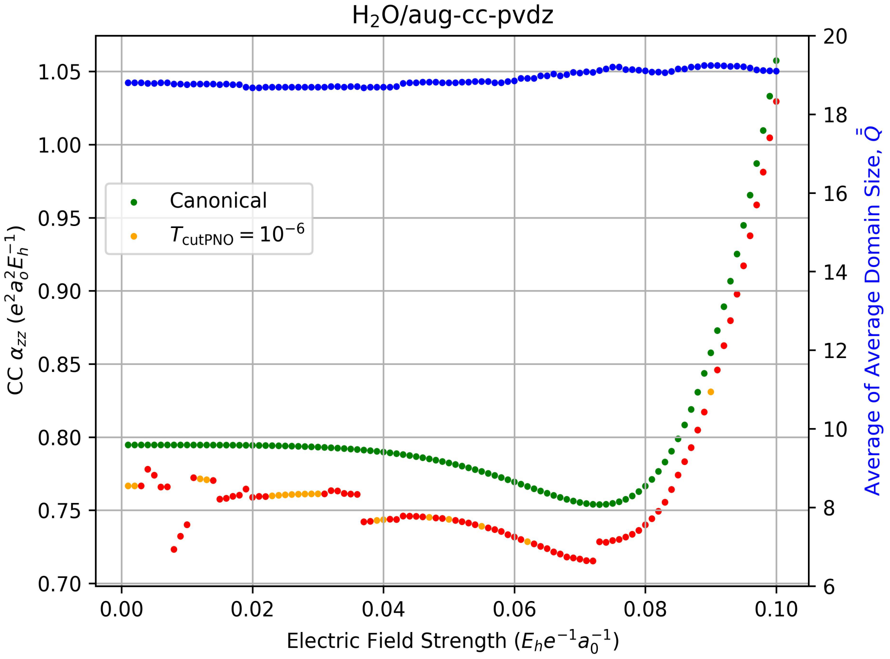

Figure S33: Correlation contribution to electric polarizability for water aug-cc-pVDZ with a $T_{\text{cutPNO}} = 10^{-6}$ as a function of external electric field strength.

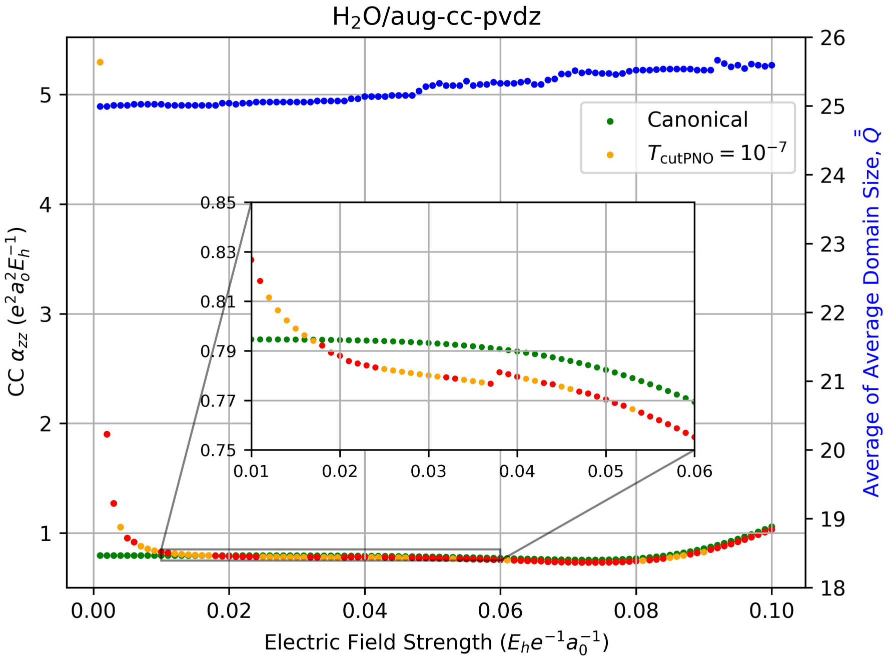

Figure S34: Correlation contribution to electric polarizability for water aug-cc-pVDZ with a $T_{\text{cutPNO}} = 10^{-7}$ as a function of external electric field strength.

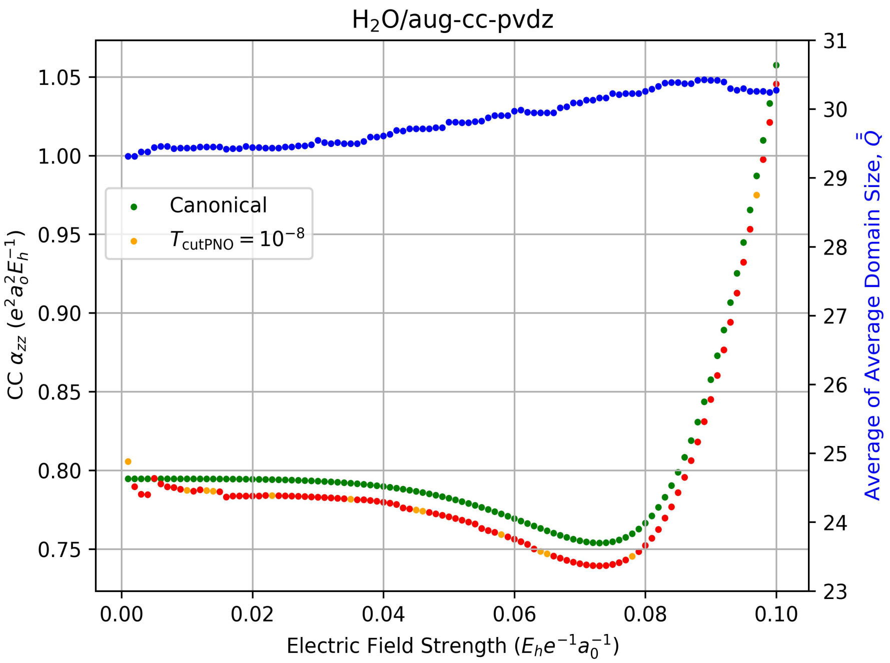

Figure S35: Correlation contribution to electric polarizability for water aug-cc-pVDZ with a $T_{\text{cutPNO}} = 10^{-8}$ as a function of external electric field strength.

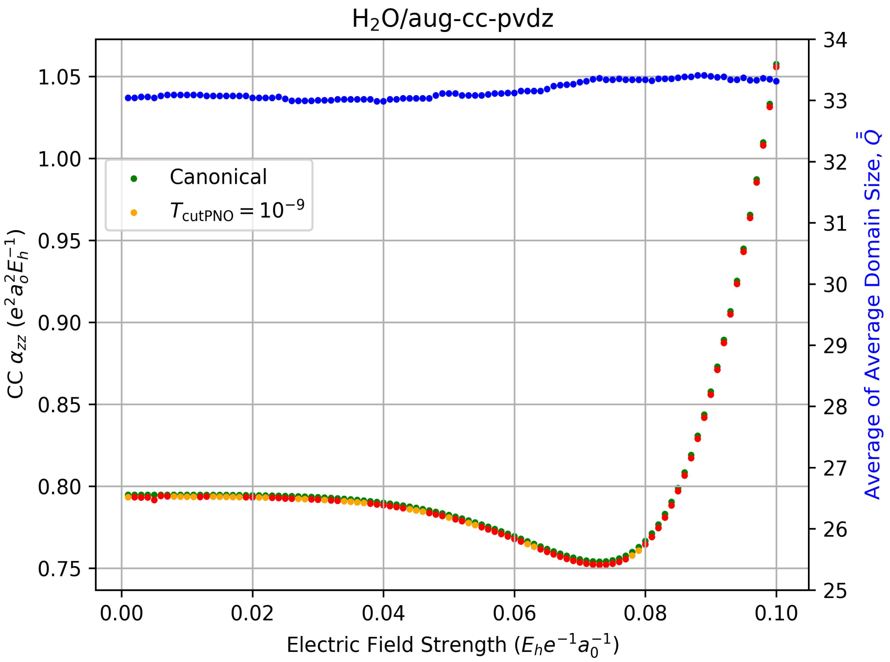

Figure S36: Correlation contribution to electric polarizability for water aug-cc-pVDZ with a $T_{\text{cutPNO}} = 10^{-9}$ as a function of external electric field strength.

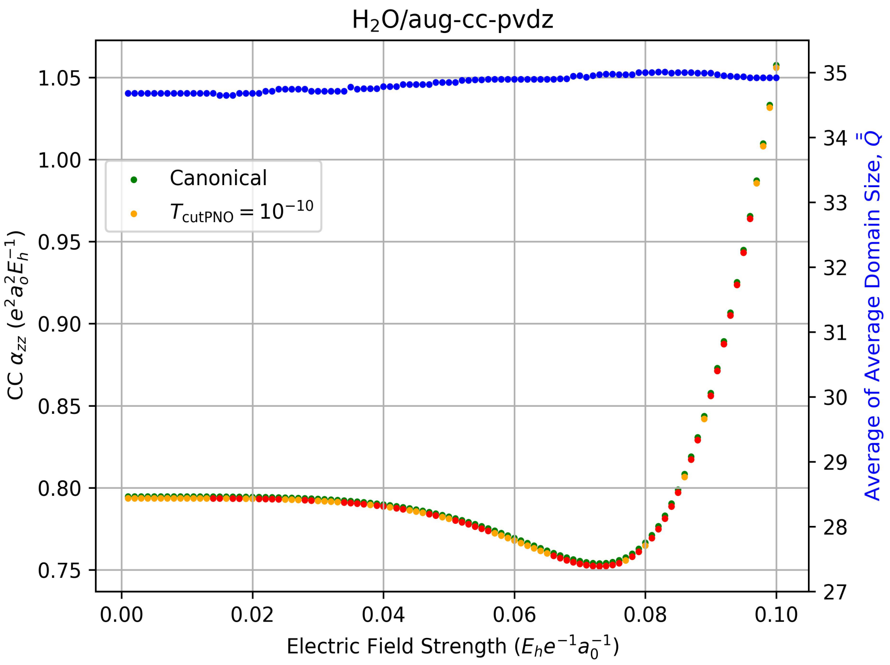

Figure S37: Correlation contribution to electric polarizability for water aug-cc-pVDZ with a $T_{\mathrm{cutPNO}} = 10^{-10}$ as a function of external electric field strength.

4.4 Frozen Core Water/cc-pVDZ

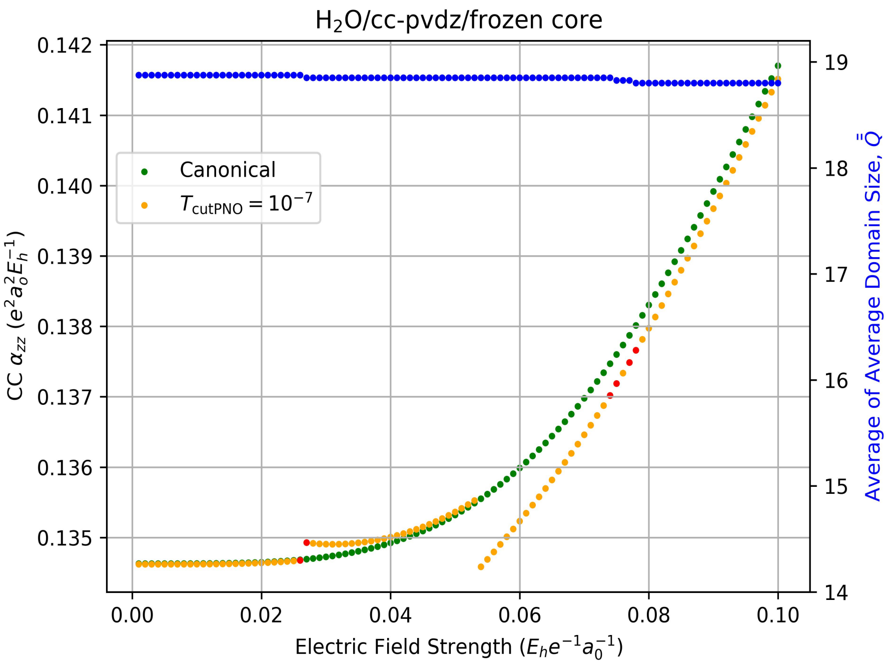

Figure S38: Correlation contribution to electric polarizability for frozen core water cc-pVDZ with a $T_{\text{cutPNO}} = 10^{-7}$ as a function of external electric field strength.

4.5 Frozen Core Water/aug-cc-pVDZ

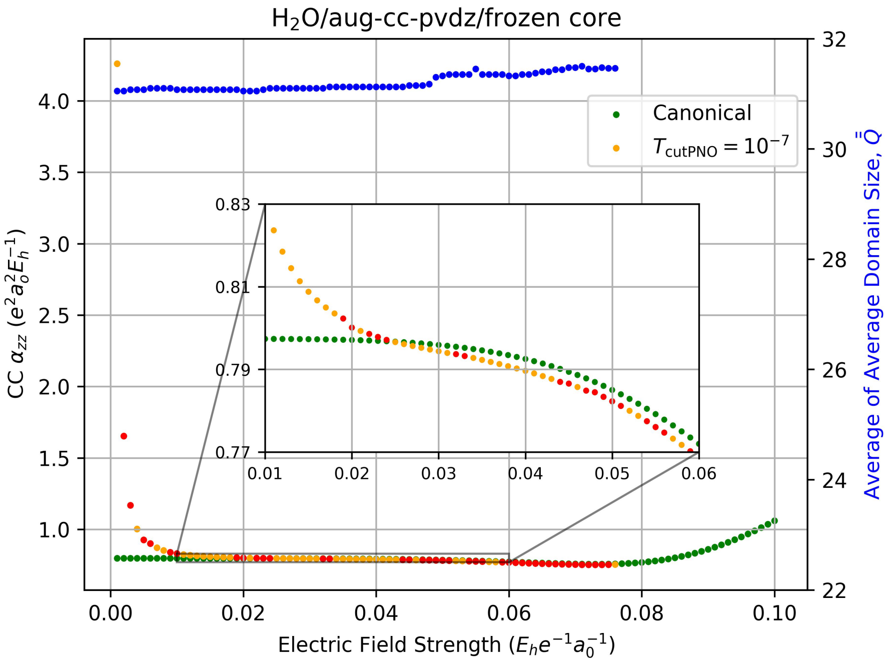

Figure S39: Correlation contribution to electric polarizability for frozen core water aug-cc-pVDZ with a $T_{\text{cutPNO}} = 10^{-7}$ as a function of external electric field strength.

4.6 HOF/cc-pVDZ

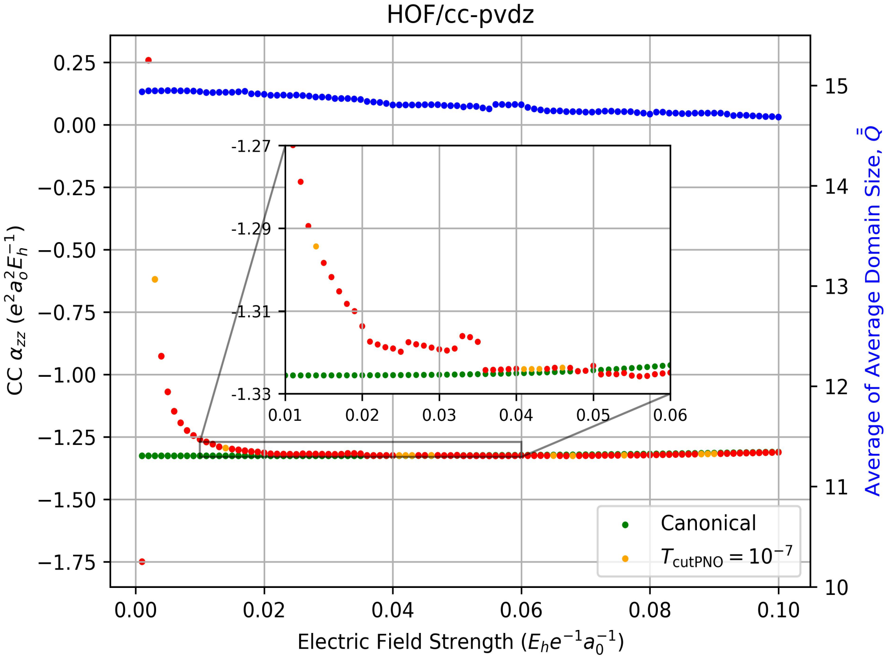

Figure S40: Correlation contribution to electric polarizability for HOF cc-pVDZ with a $T_{\mathrm{cutPNO}} = 10^{-7}$ as a function of external electric field strength.

4.7 HOF/aug-cc-pVDZ

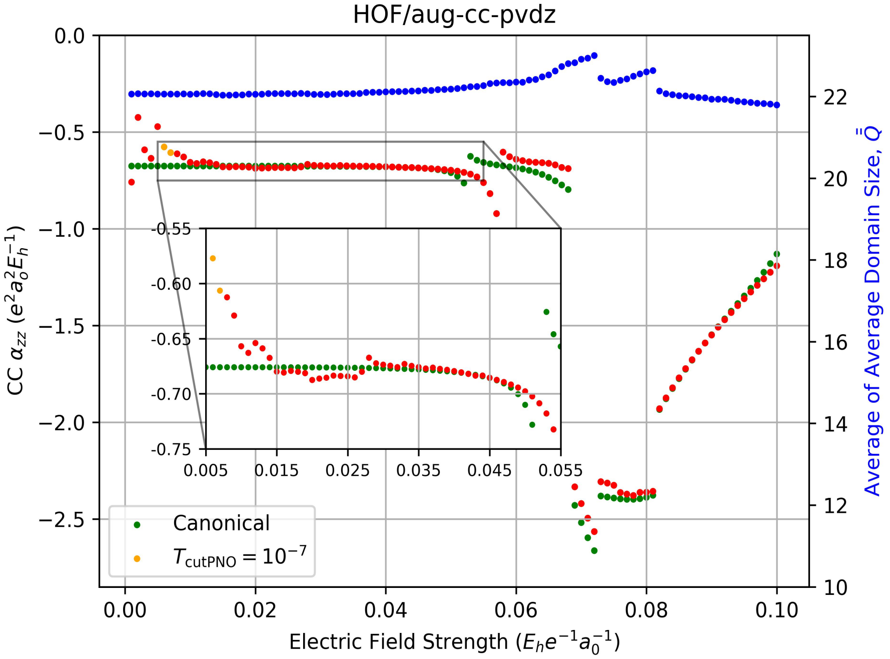

Figure S41: Correlation contribution to electric polarizability for HOF aug-cc-pVDZ with a $T_{\text{cutPNO}} = 10^{-7}$ as a function of external electric field strength.

4.8 Fluoroethylene/cc-pVDZ

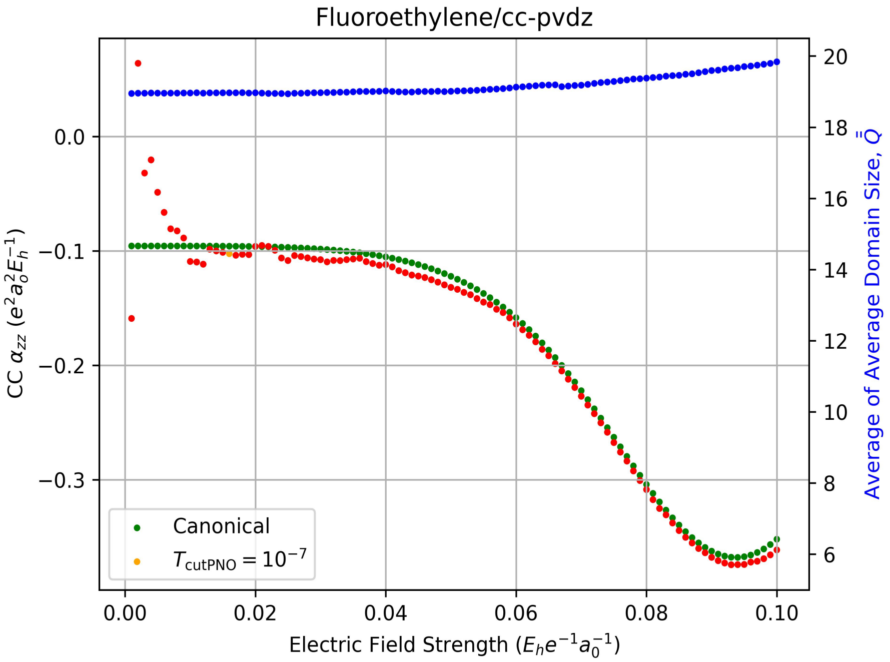

Figure S42: Correlation contribution to electric polarizability for fluoroethylene cc-pVDZ with a $T_{\mathrm{cutPNO}} = 10^{-7}$ as a function of external electric field strength.

4.9 Fluoroethylene/aug-cc-pVDZ

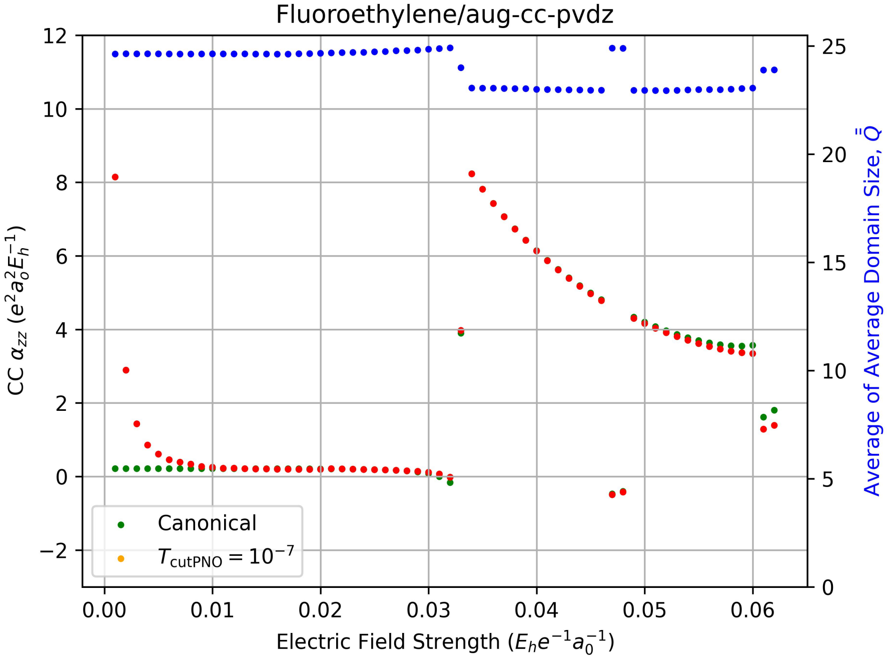

Figure S43: Correlation contribution to electric polarizability for fluoroethylene aug-cc-pVDZ with a $T_{\text{cutPNO}} = 10^{-7}$ as a function of external electric field strength.

4.10 Butadiene/cc-pVDZ

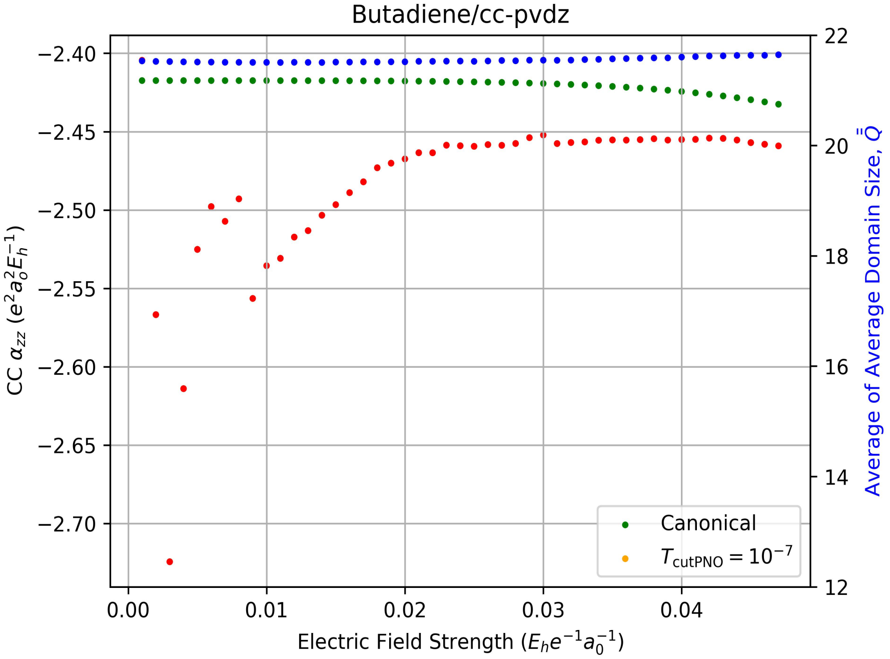

Figure S44: Correlation contribution to electric polarizability for butadiene cc-pVDZ with a $T_{\text{cutPNO}} = 10^{-7}$ as a function of external electric field strength.

4.11 Butadiene/aug-cc-pVDZ

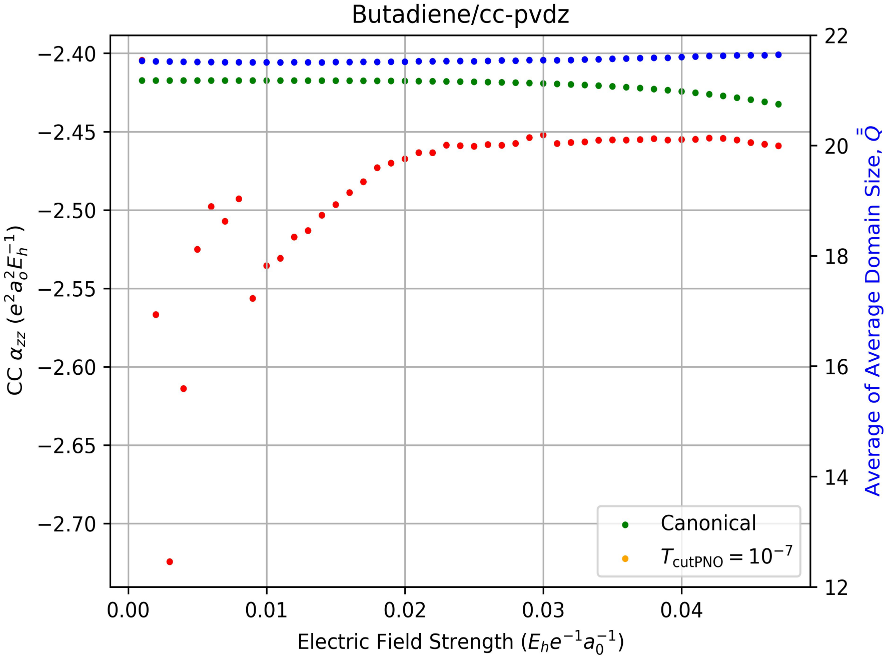

Figure S45: Correlation contribution to electric polarizability for butadiene cc-pVDZ with a $T_{\mathrm{cutPNO}} = 10^{-7}$ as a function of external electric field strength.

5 Correlation Contribution to Electric Hyperpolarizabilities

5.1 Water/cc-pVDZ

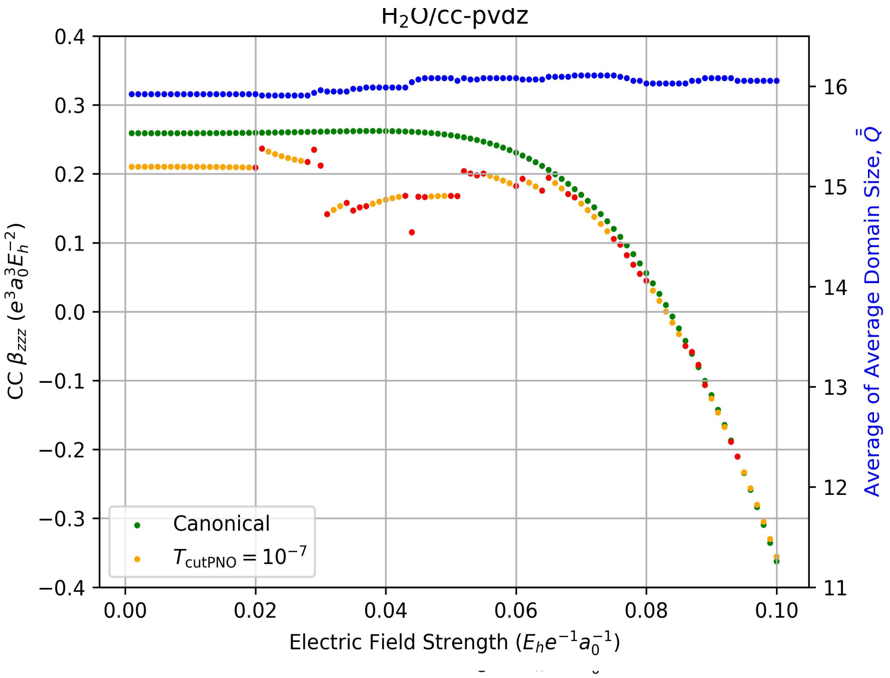

Figure S46: Correlation contribution to electric hyperpolarizability for water cc-pVDZ with a $T_{\text{cutPNO}} = 10^{-7}$ as a function of external electric field strength.

5.2 PNO-Relaxed Water/cc-pVDZ

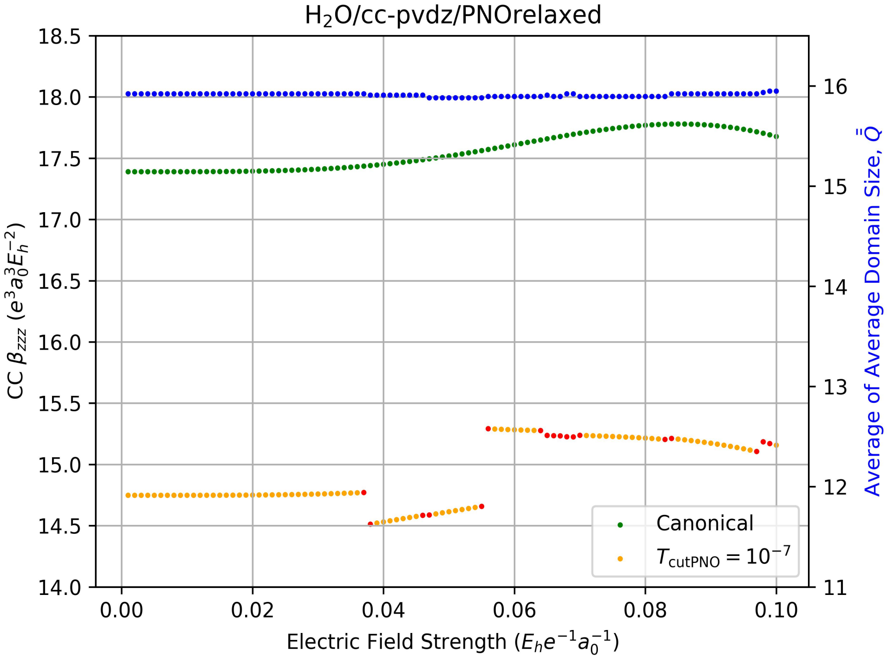

Figure S47: Correlation contribution to electric hyperpolarizability for PNO-relaxed water cc-pVDZ with a $T_{\mathrm{cutPNO}} = 10^{-7}$ as a function of external electric field strength.

5.3 Water/aug-cc-pVDZ

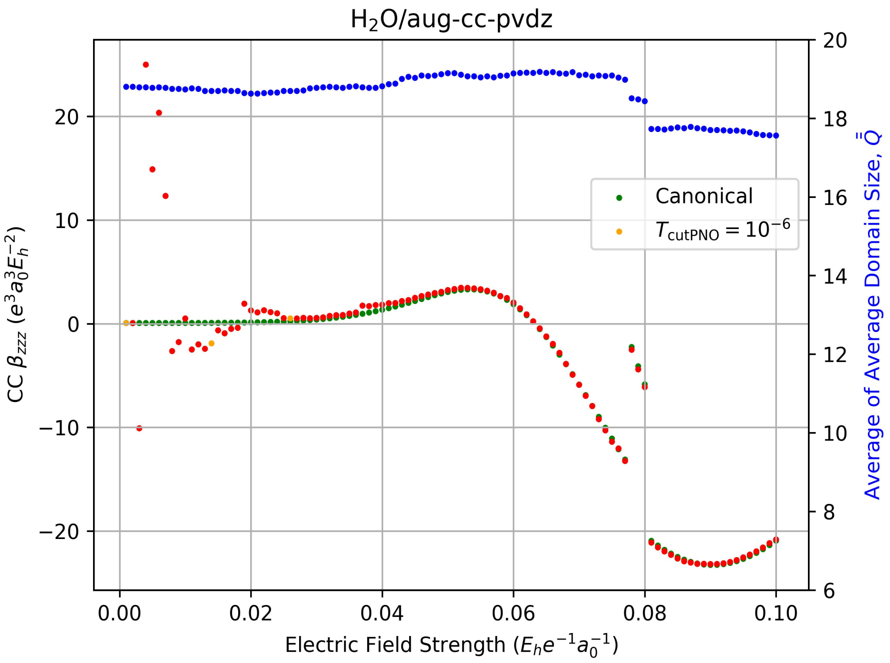

Figure S48: Correlation contribution to electric hyperpolarizability for water aug-cc-pVDZ with a $T_{\text{cutPNO}} = 10^{-6}$ as a function of external electric field strength.

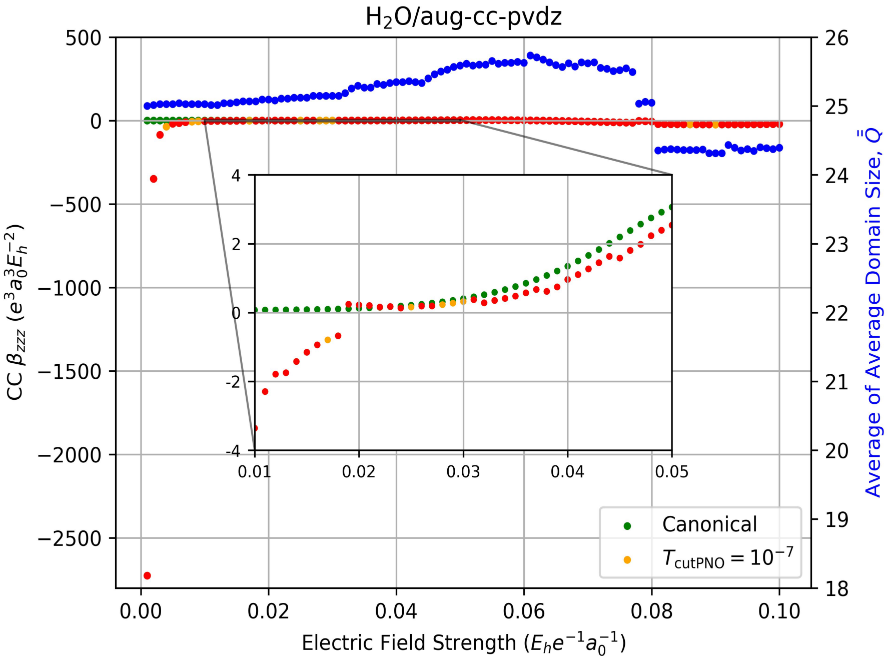

Figure S49: Correlation contribution to electric hyperpolarizability for water aug-cc-pVDZ with a $T_{\text{cutPNO}} = 10^{-7}$ as a function of external electric field strength.

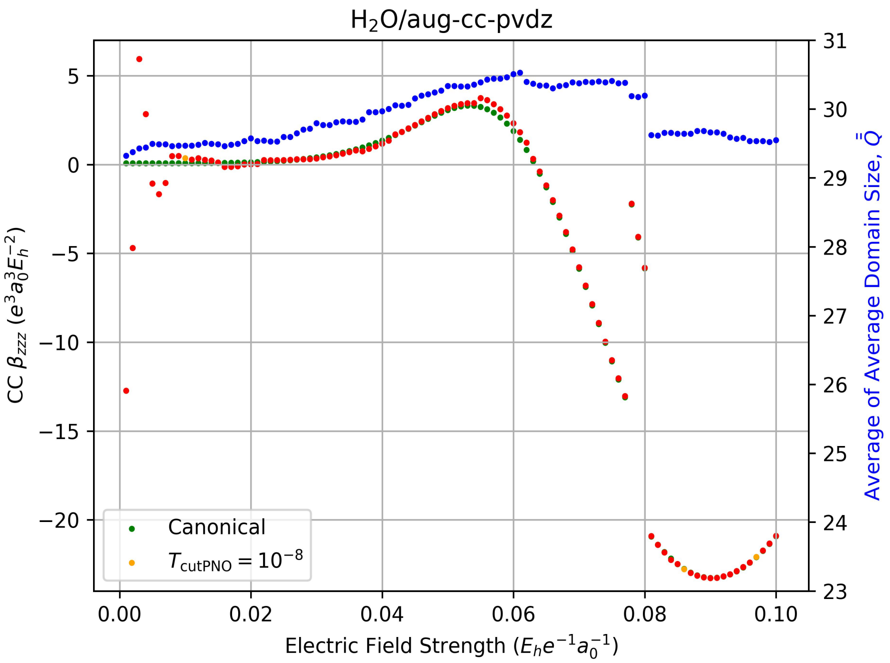

Figure S50: Correlation contribution to electric hyperpolarizability for water aug-cc-pVDZ with a $T_{\mathrm{cutPNO}} = 10^{-8}$ as a function of external electric field strength.

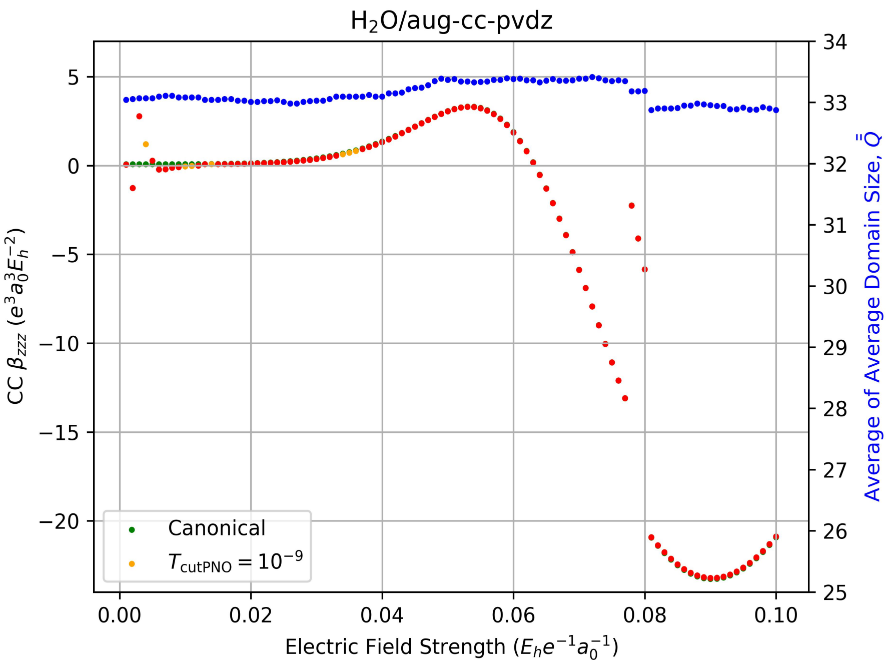

Figure S51: Correlation contribution to electric hyperpolarizability for water aug-cc-pVDZ with a $T_{\mathrm{cutPNO}} = 10^{-9}$ as a function of external electric field strength.

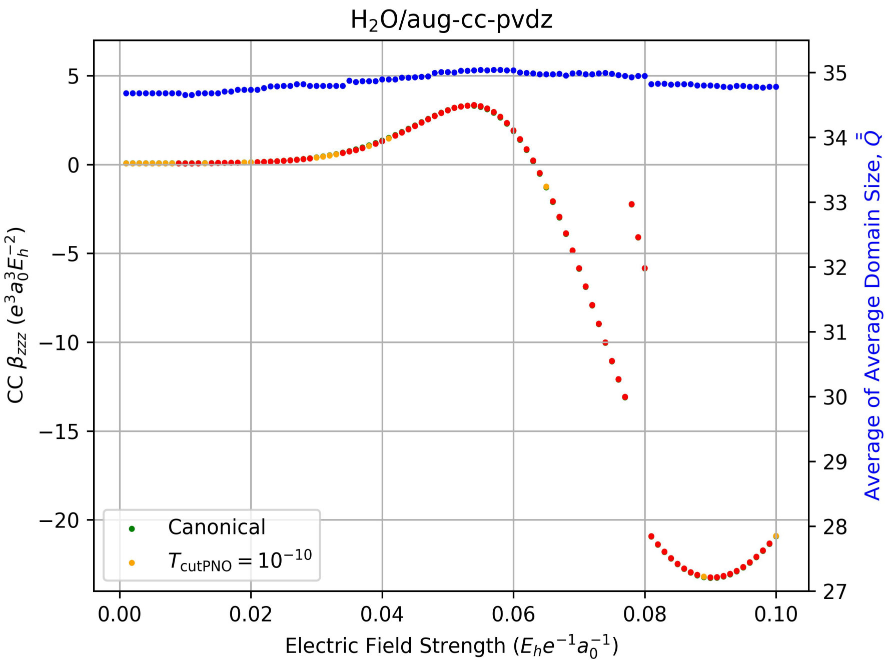

Figure S52: Correlation contribution to electric hyperpolarizability for water aug-cc-pVDZ with a $T_{\text{cutPNO}} = 10^{-10}$ as a function of external electric field strength.

5.4 Frozen Core Water/cc-pVDZ

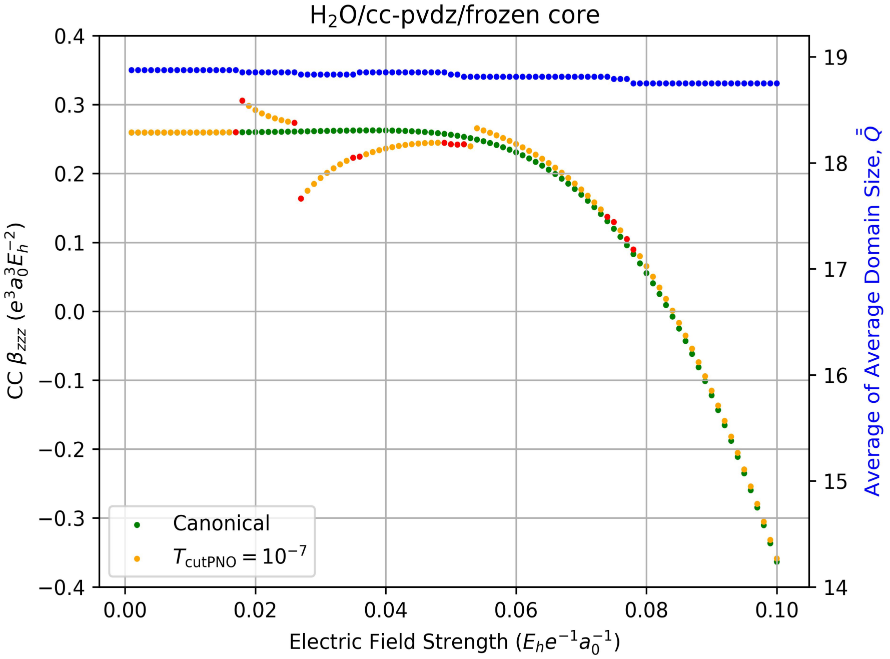

Figure S53: Correlation contribution to electric hyperpolarizability for frozen core water cc-pVDZ with a $T_{\text{cutPNO}} = 10^{-7}$ as a function of external electric field strength.

5.5 Frozen Core Water/aug-cc-pVDZ

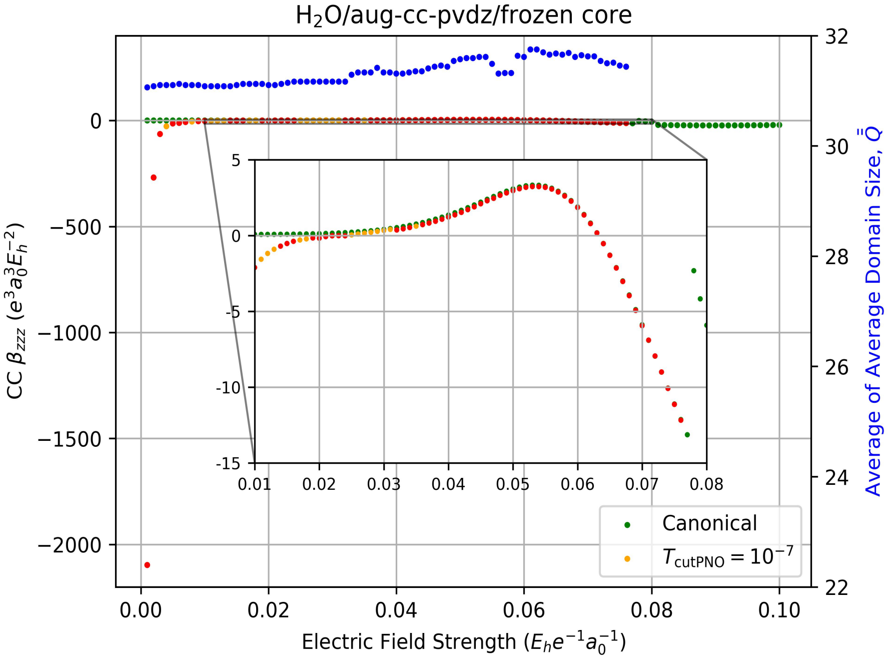

Figure S54: Correlation contribution to electric hyperpolarizability for frozen core water aug-cc-pVDZ with a $T_{\mathrm{cutPNO}} = 10^{-7}$ as a function of external electric field strength.

5.6 HOF/cc-pVDZ

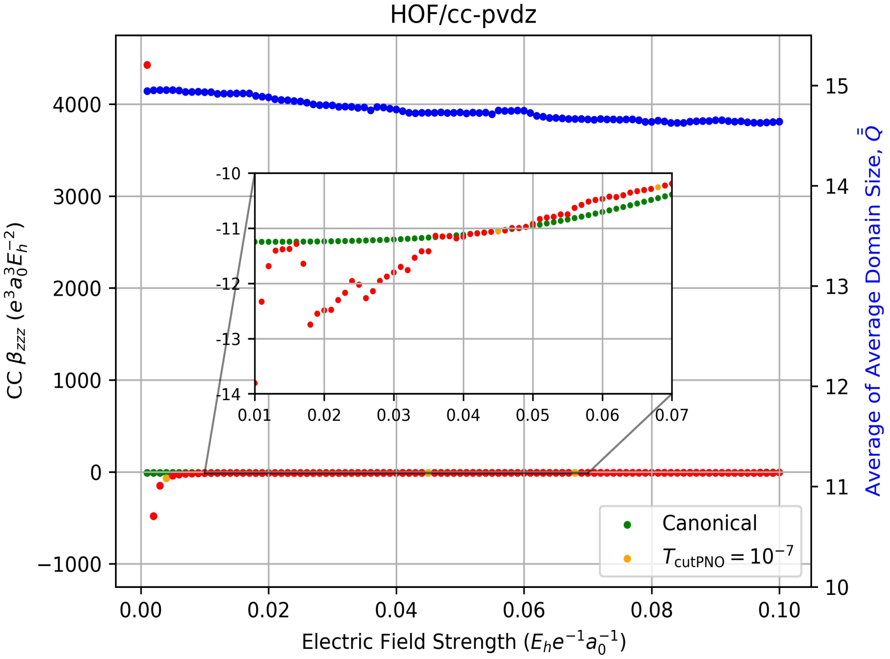

Figure S55: Correlation contribution to electric hyperpolarizability for HOF cc-pVDZ with a $T_{\text{cutPNO}} = 10^{-7}$ as a function of external electric field strength.

5.7 HOF/aug-cc-pVDZ

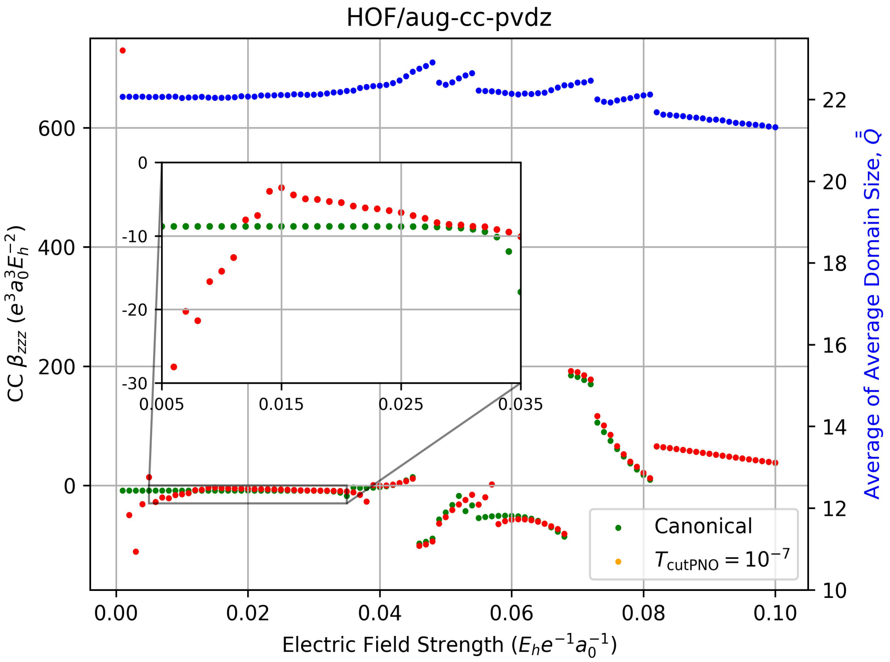

Figure S56: Correlation contribution to electric hyperpolarizability for HOF aug-cc-pVDZ with a $T_{\mathrm{cutPNO}} = 10^{-7}$ as a function of external electric field strength.

5.8 Fluoroethylene/cc-pVDZ

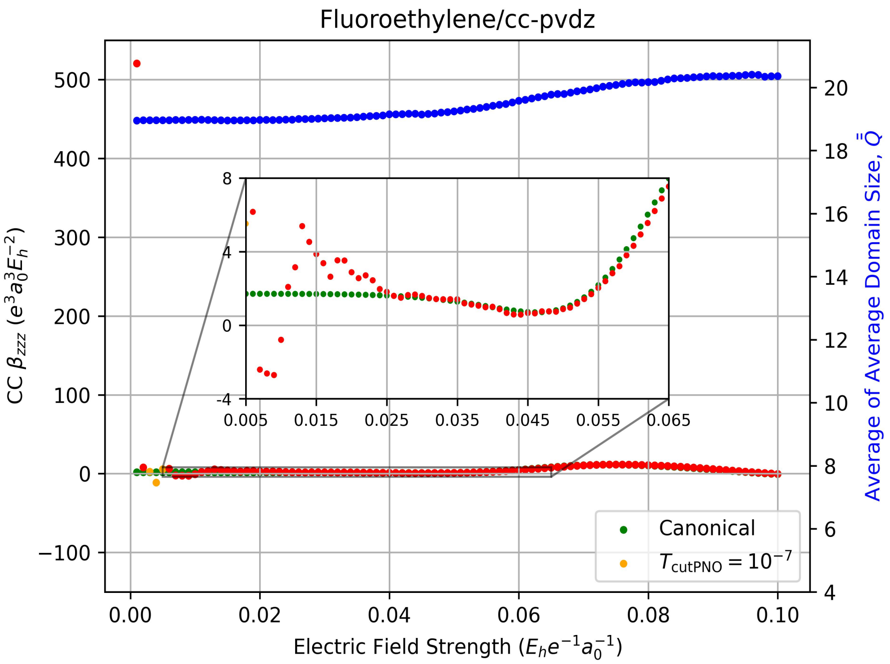

Figure S57: Correlation contribution to electric hyperpolarizability for fluoroethylene cc-pVDZ with a $T_{\text{cutPNO}} = 10^{-7}$ as a function of external electric field strength.

5.9 Fluoroethylene/aug-cc-pVDZ

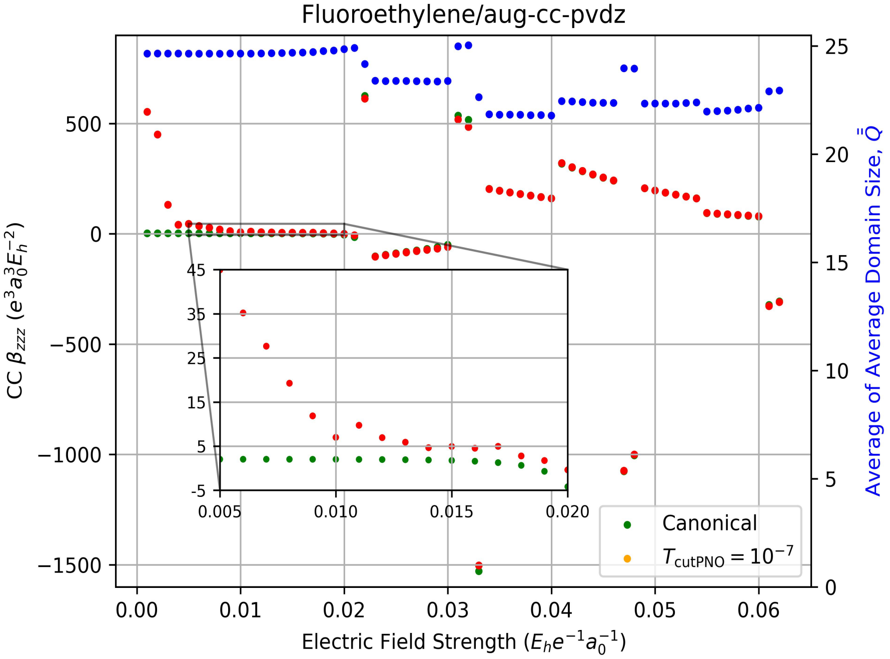

Figure S58: Correlation contribution to electric hyperpolarizability for fluoroethylene aug-cc-pVDZ with a $T_{\text{cutPNO}} = 10^{-7}$ as a function of external electric field strength.

5.10 Butadiene/cc-pVDZ

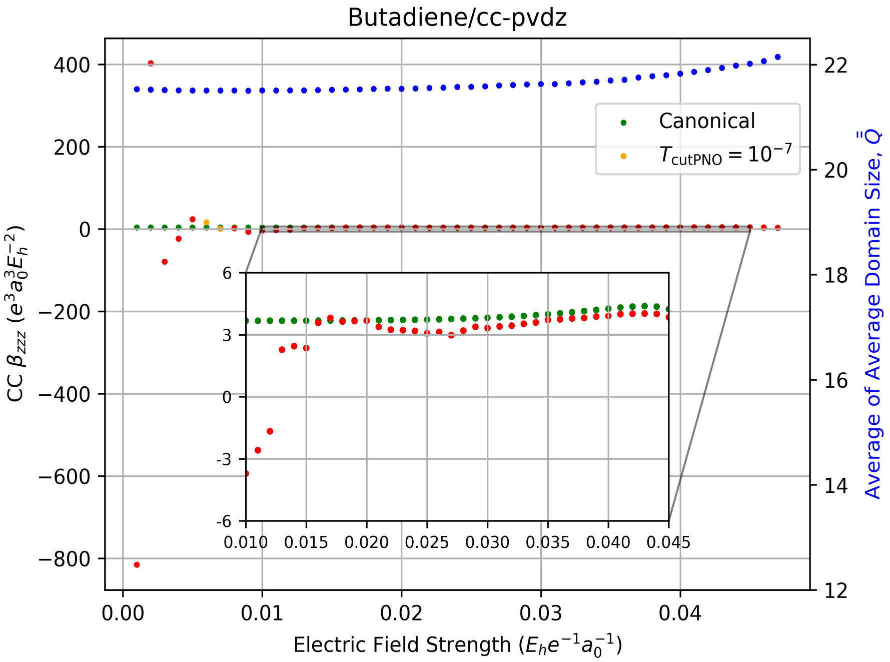

Figure S59: Correlation contribution to electric hyperpolarizability for butadiene cc-pVDZ with a $T_{\text{cutPNO}} = 10^{-7}$ as a function of external electric field strength.

5.11 Butadiene/aug-cc-pVDZ

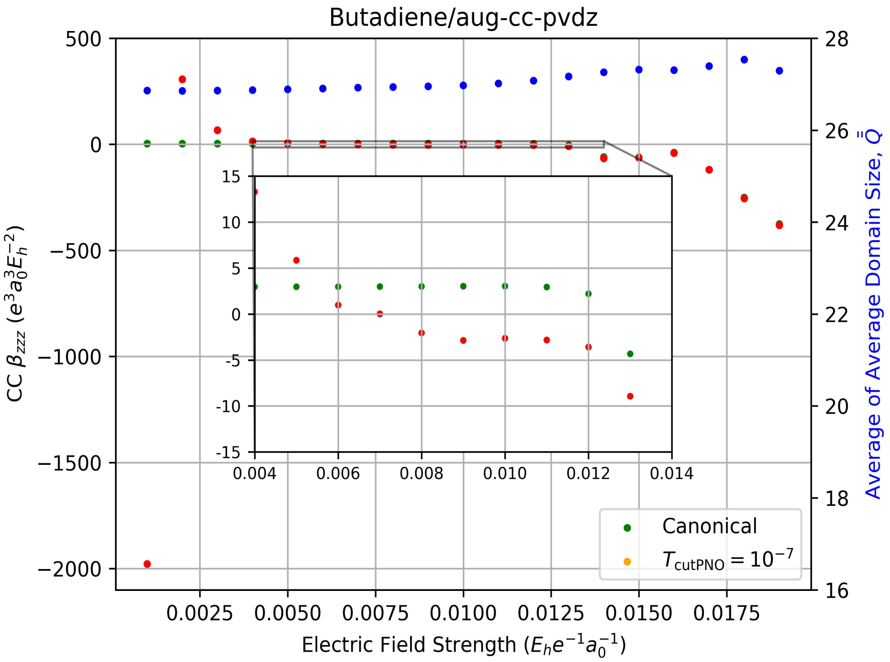

Figure S60: Correlation contribution to electric hyperpolarizability for butadiene aug-cc-pVDZ with a $T_{\text{cutPNO}} = 10^{-7}$ as a function of external electric field strength.

6 Total Energies

6.1 Water/cc-pVDZ

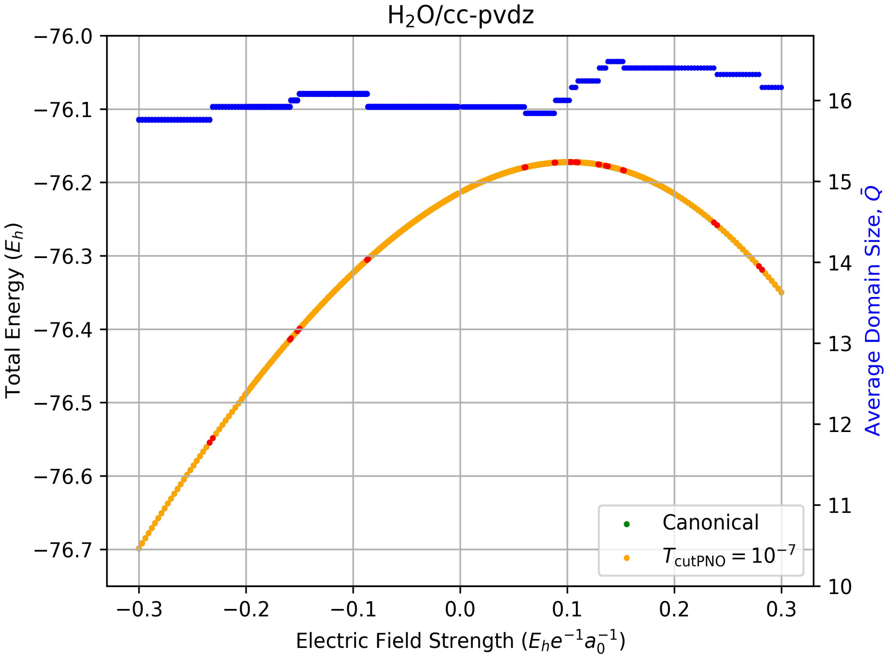

Figure S61: Total energies for water cc-pVDZ with a $T_{\mathrm{cutPNO}} = 10^{-7}$ as a function of external electric field strength.

6.2 PNO-Relaxed Water/cc-pVDZ

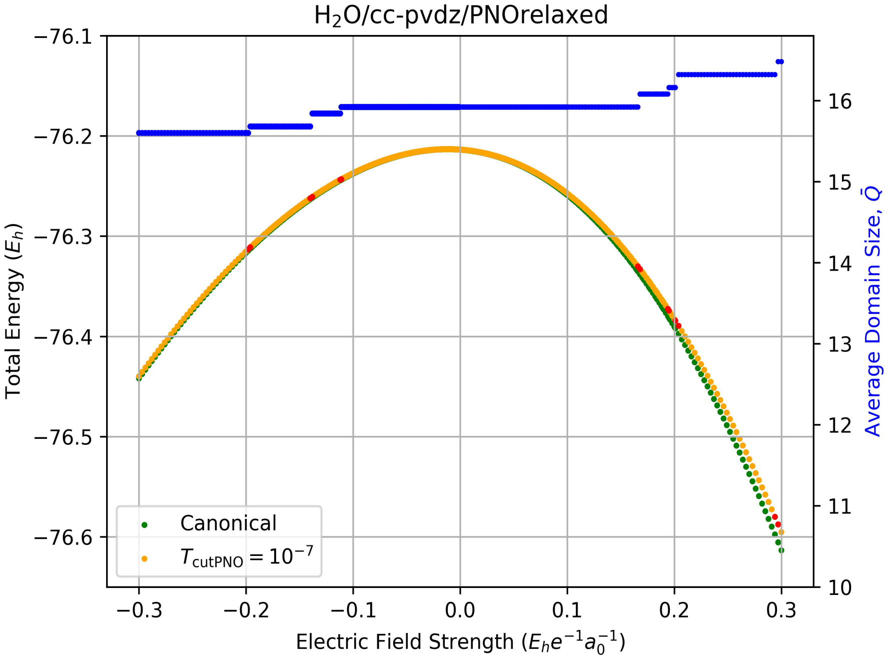

Figure S62: Total energies for PNO-relaxed water cc-pVDZ with a $T_{\mathrm{cutPNO}} = 10^{-7}$ as a function of external electric field strength.

6.3 Water/aug-cc-pVDZ

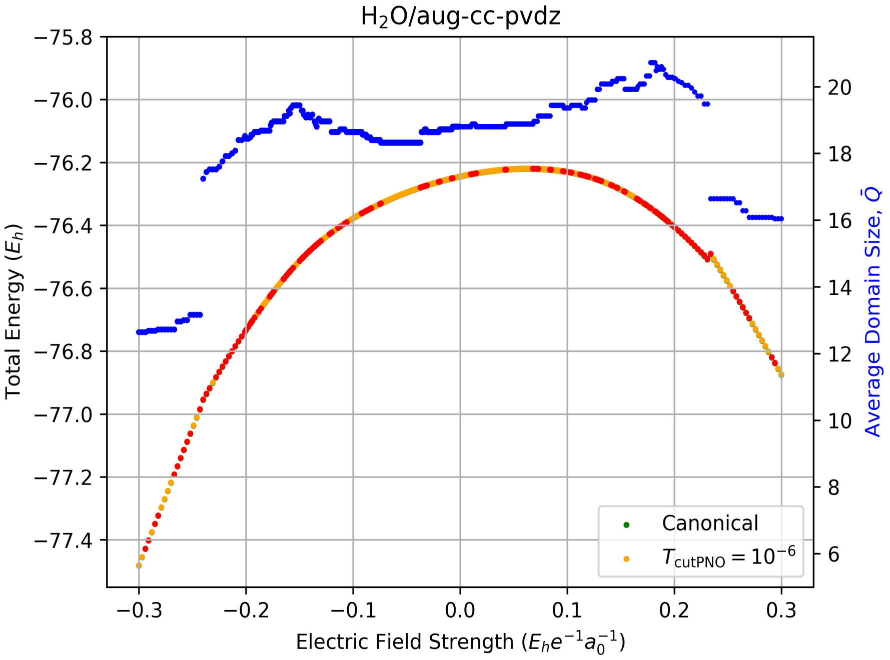

Figure S63: Total energies for water aug-cc-pVDZ with a $T_{\text{cutPNO}} = 10^{-6}$ as a function of external electric field strength.

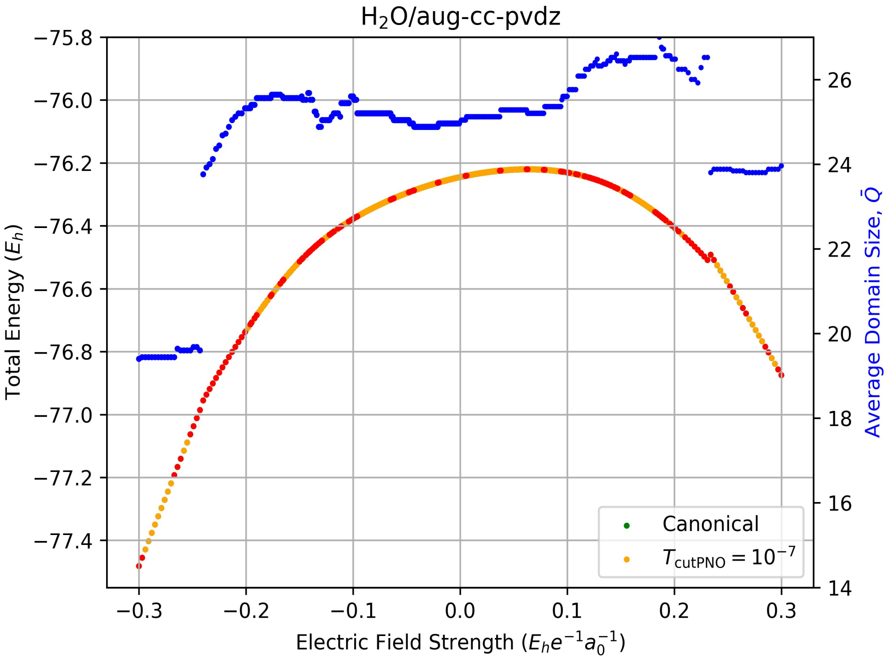

Figure S64: Total energies for water aug-cc-pVDZ with a $T_{\text{cutPNO}} = 10^{-7}$ as a function of external electric field strength.

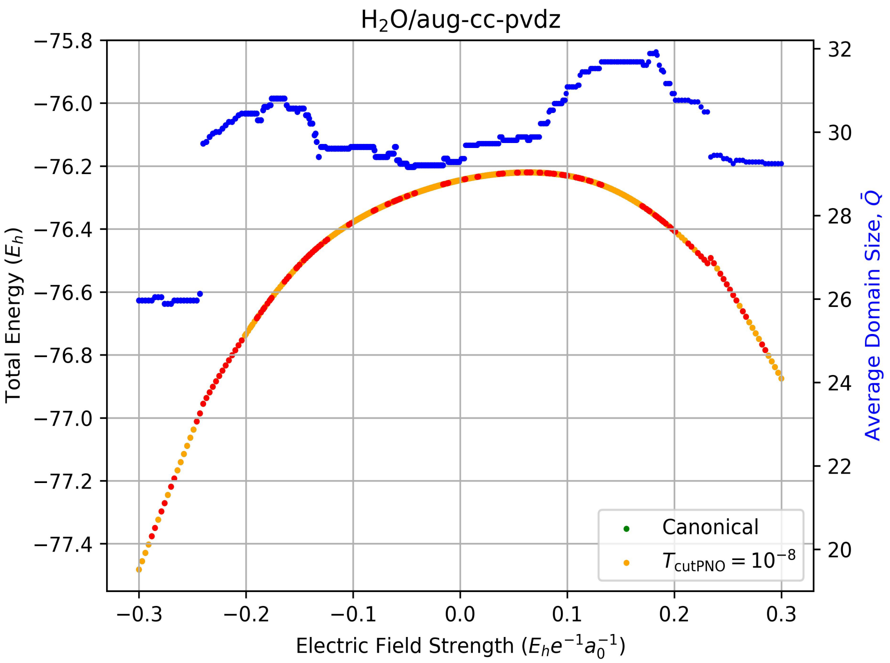

Figure S65: Total energies for water aug-cc-pVDZ with a $T_{\text{cutPNO}} = 10^{-8}$ as a function of external electric field strength.

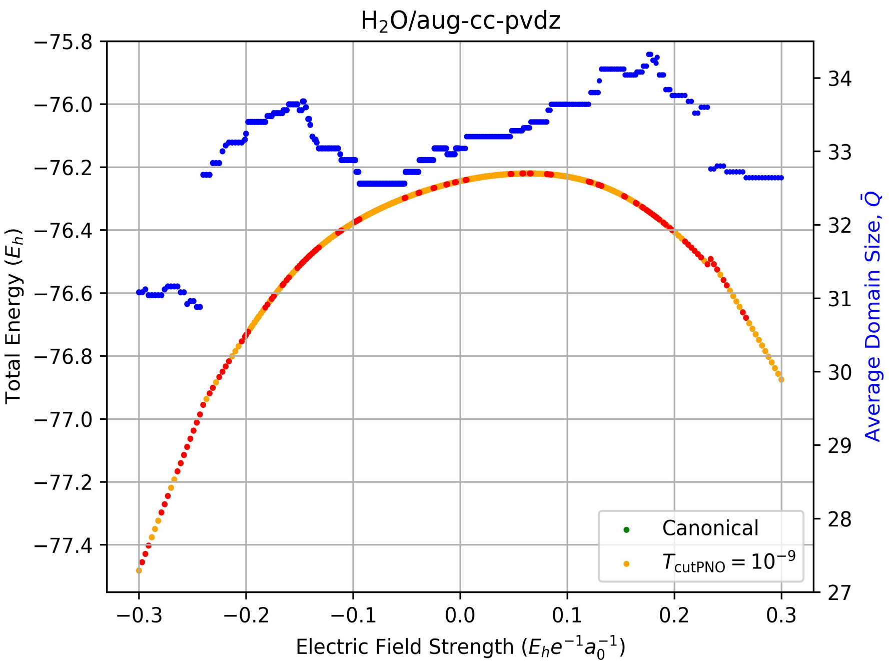

Figure S66: Total energies for water aug-cc-pVDZ with a $T_{\text{cutPNO}} = 10^{-9}$ as a function of external electric field strength.

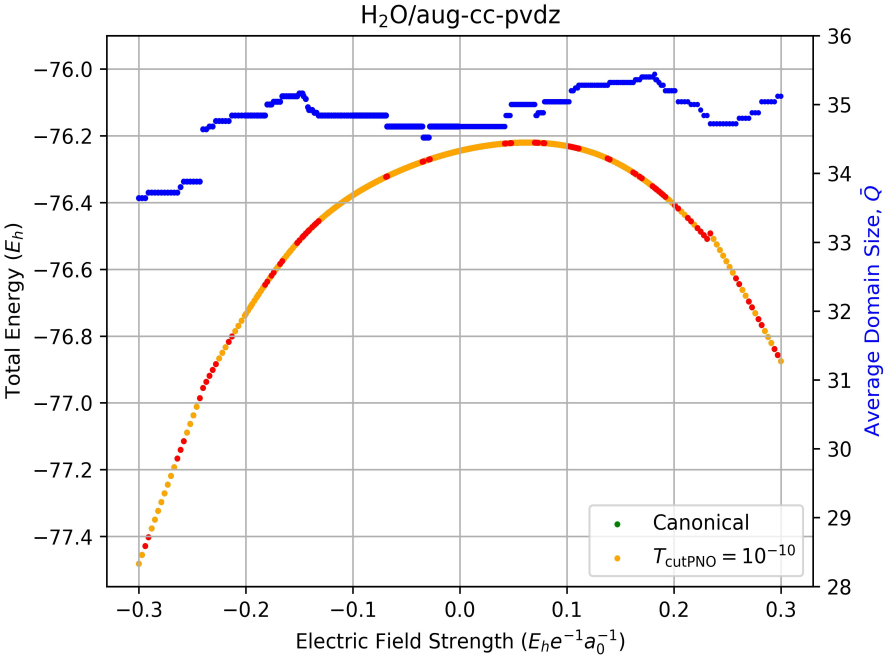

Figure S67: Total energies for water aug-cc-pVDZ with a $T_{\text{cutPNO}} = 10^{-10}$ as a function of external electric field strength.

6.4 Frozen Core Water/cc-pVDZ

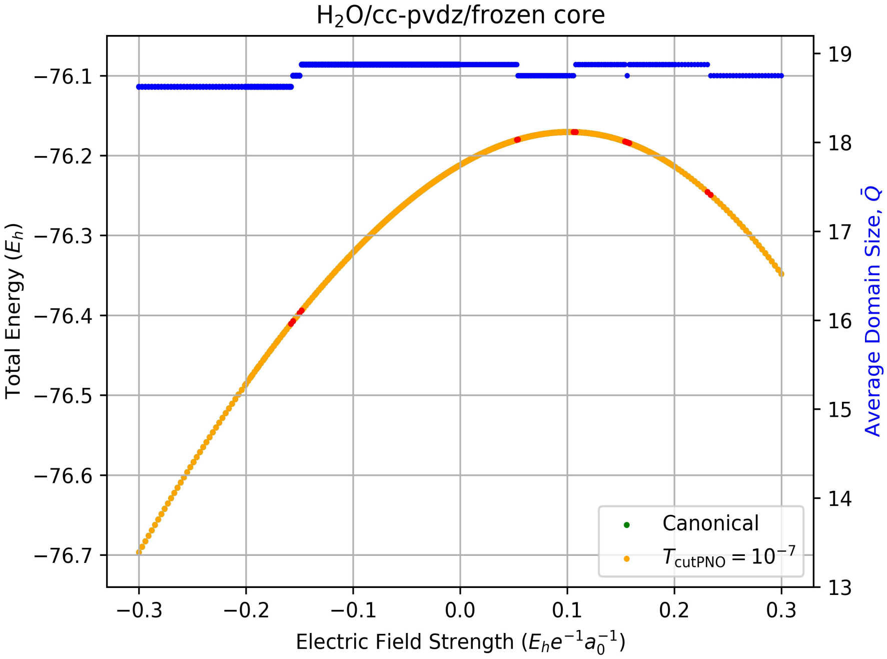

Figure S68: Total energies for frozen core water cc-pVDZ with a $T_{\mathrm{cutPNO}} = 10^{-7}$ as a function of external electric field strength.

6.5 Frozen Core Water/aug-cc-pVDZ

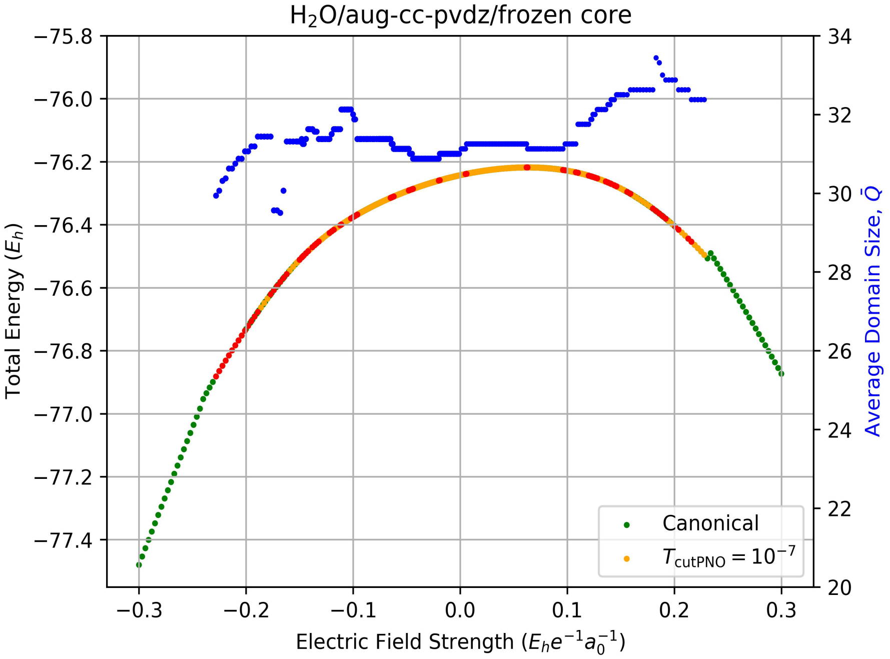

Figure S69: Total energies for frozen core water aug-cc-pVDZ with a $T_{\mathrm{cutPNO}} = 10^{-7}$ as a function of external electric field strength.

6.6 HOF/cc-pVDZ

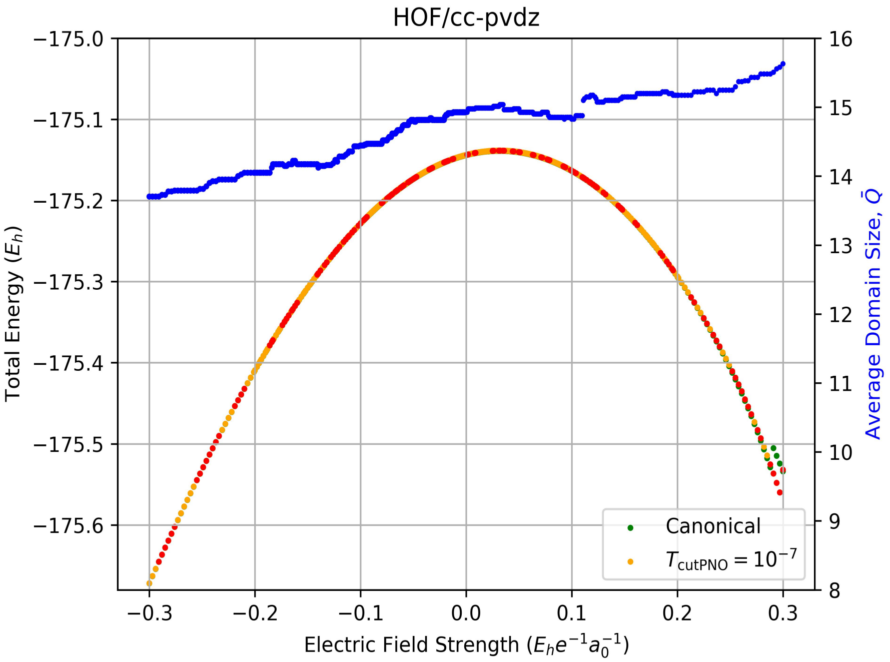

Figure S70: Total energies for HOF cc-pVDZ with a $T_{\mathrm{cutPNO}} = 10^{-7}$ as a function of external electric field strength.

6.7 HOF/aug-cc-pVDZ

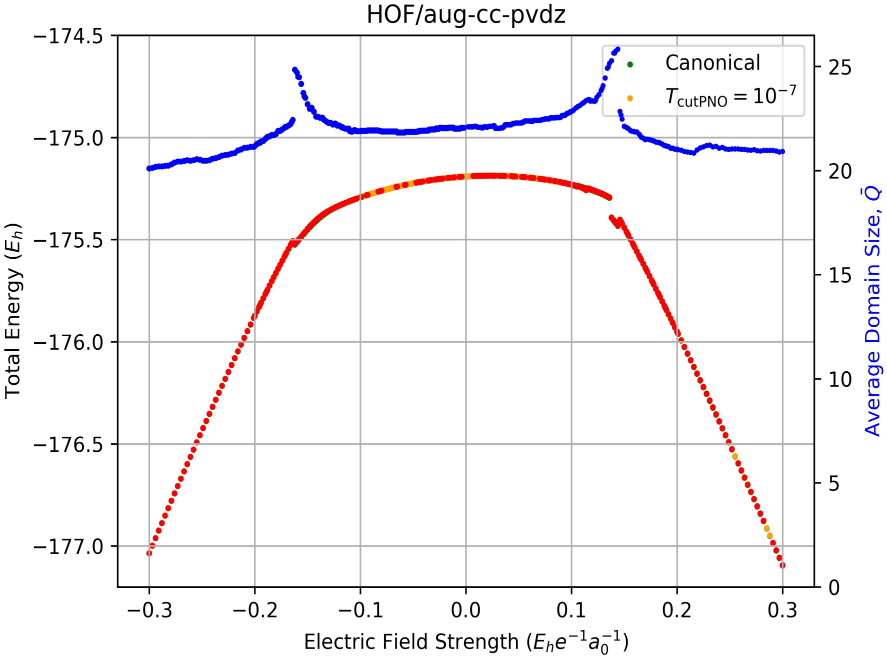

Figure S71: Total energies for HOF aug-cc-pVDZ with a $T_{\text{cutPNO}} = 10^{-7}$ as a function of external electric field strength.

6.8 Fluoroethylene/cc-pVDZ

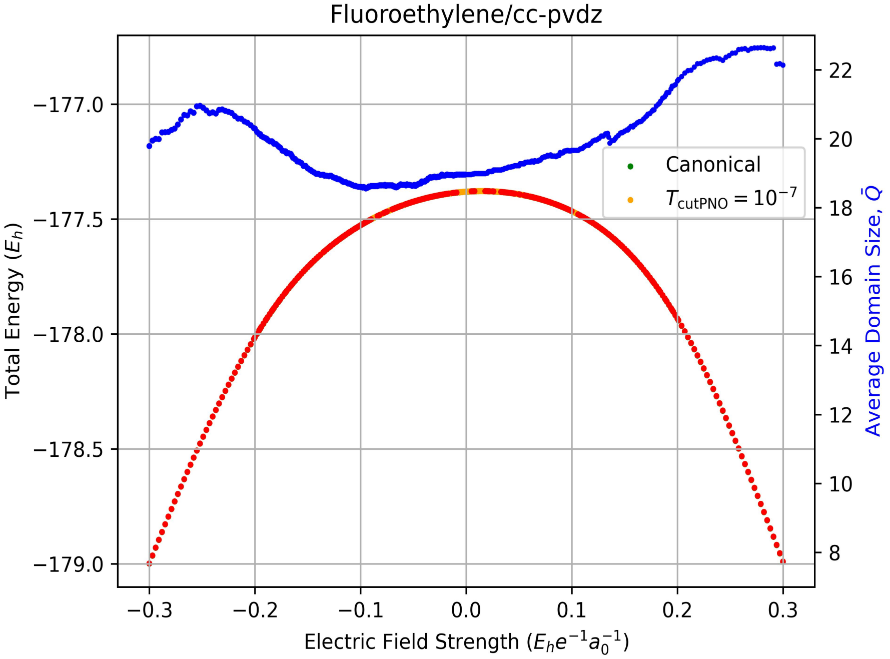

Figure S72: Total energies for fluoroethylene cc-pVDZ with a $T_{\mathrm{cutPNO}} = 10^{-7}$ as a function of external electric field strength.

6.9 Fluoroethylene/aug-cc-pVDZ

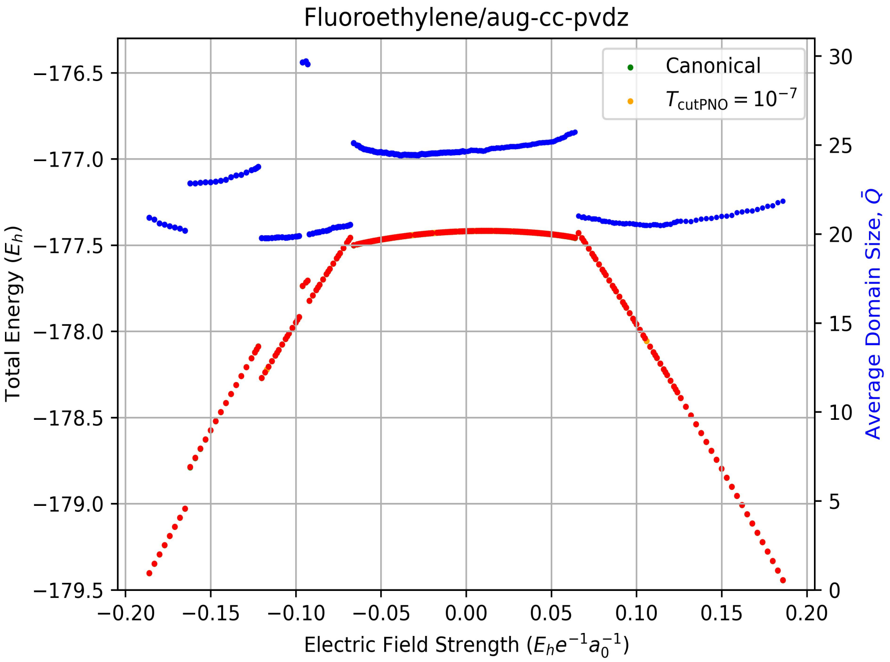

Figure S73: Total energies for fluoroethylene aug-cc-pVDZ with a $T_{\text{cutPNO}} = 10^{-7}$ as a function of external electric field strength.

6.10 Butadiene/cc-pVDZ

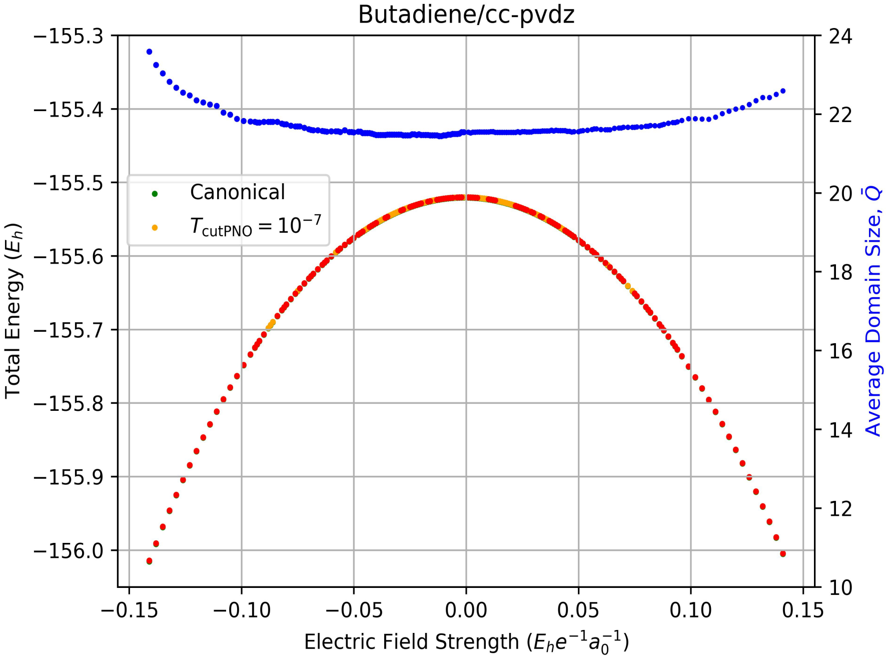

Figure S74: Total energies for butadiene cc-pVDZ with a $T_{\text{cutPNO}} = 10^{-7}$ as a function of external electric field strength.

6.11 Butadiene/aug-cc-pVDZ

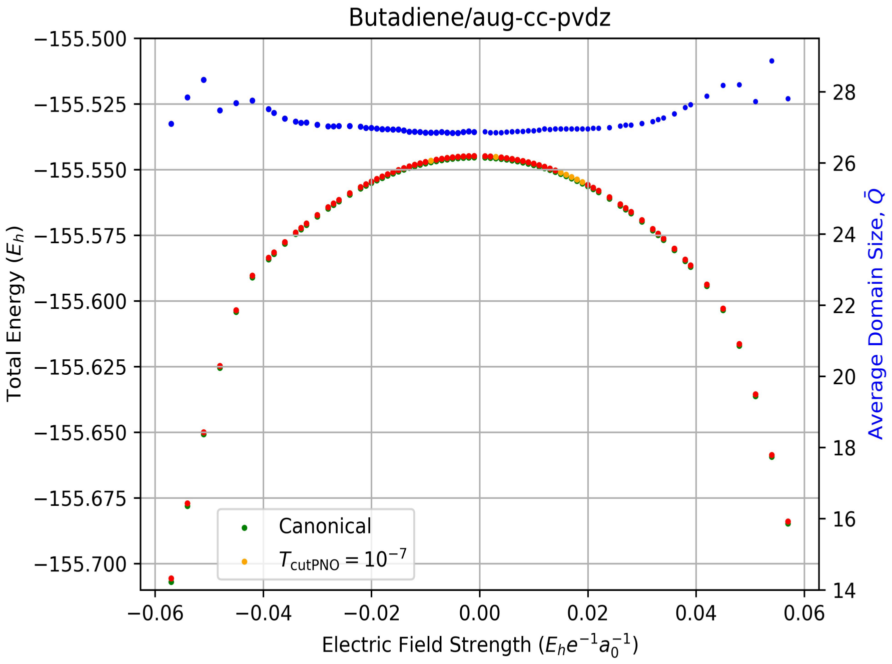

Figure S75: Total energies for butadiene aug-cc-pVDZ with a $T_{\mathrm{cutPNO}} = 10^{-7}$ as a function of external electric field strength.

7 Total Contribution to to Electric Dipole Moments

7.1 Water/cc-pVDZ

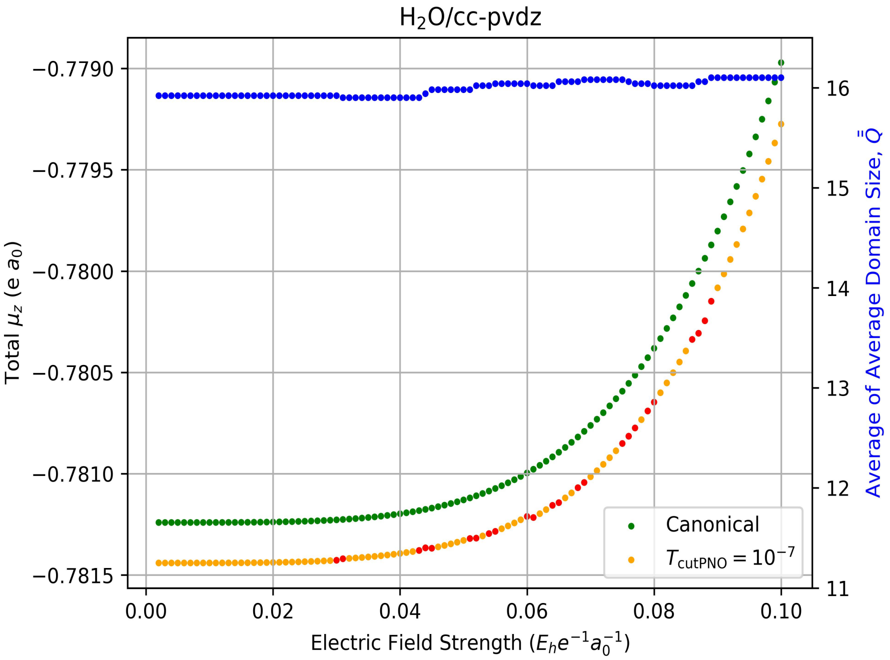

Figure S76: Total contribution to electric dipole moment for water cc-pVDZ with a T_{cutPNO} $= 10^{-7}$ as a function of external electric field strength.

7.2 PNO-Relaxed Water/cc-pVDZ

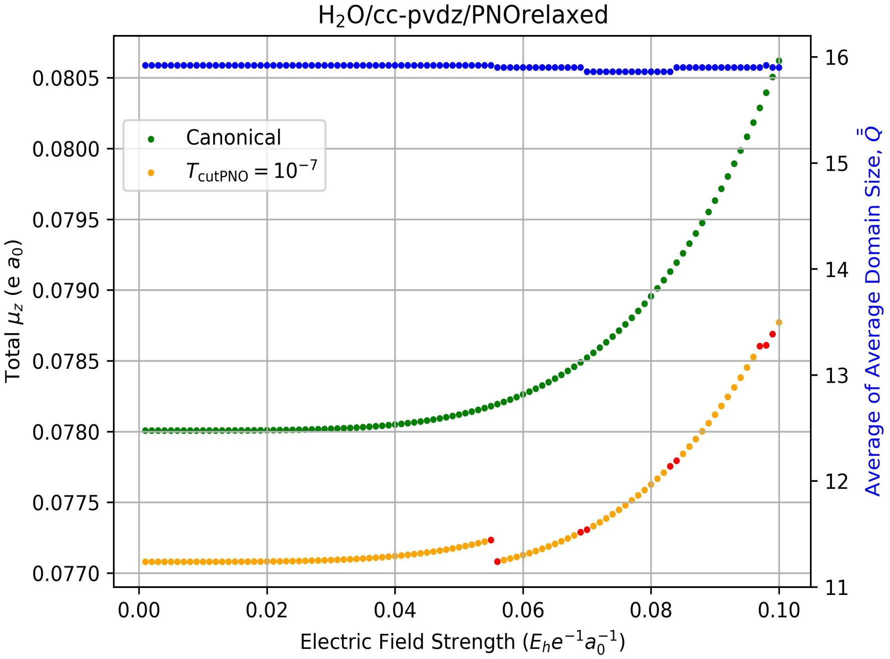

Figure S77: Total contribution to electric dipole moment for PNO-relaxed water cc-pVDZ with a $T_{\text{cutPNO}} = 10^{-7}$ as a function of external electric field strength.

7.3 Water/aug-cc-pVDZ

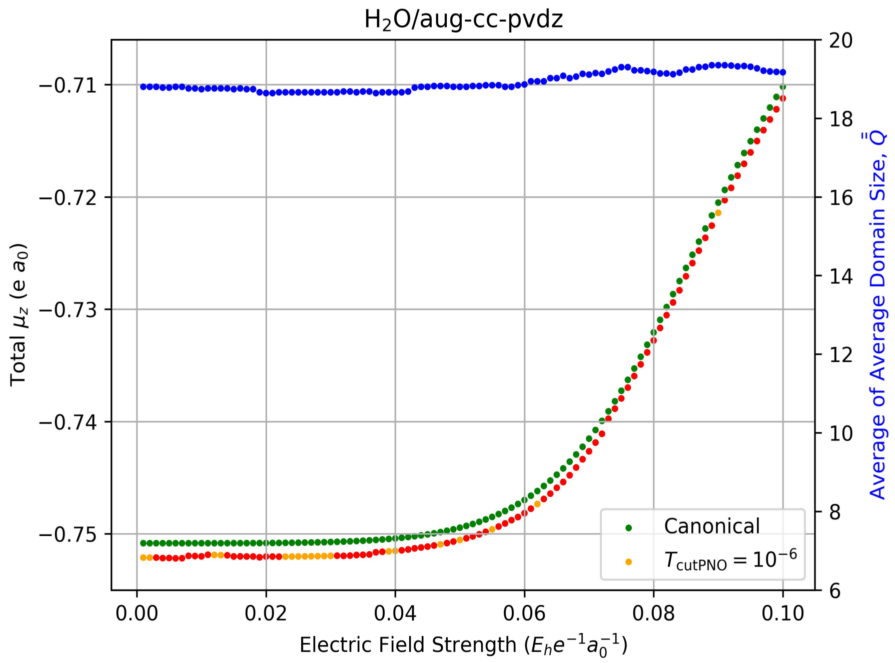

Figure S78: Total contribution to electric dipole moment for water aug-cc-pVDZ with a $T_{\mathrm{cutPNO}} = 10^{-6}$ as a function of external electric field strength.

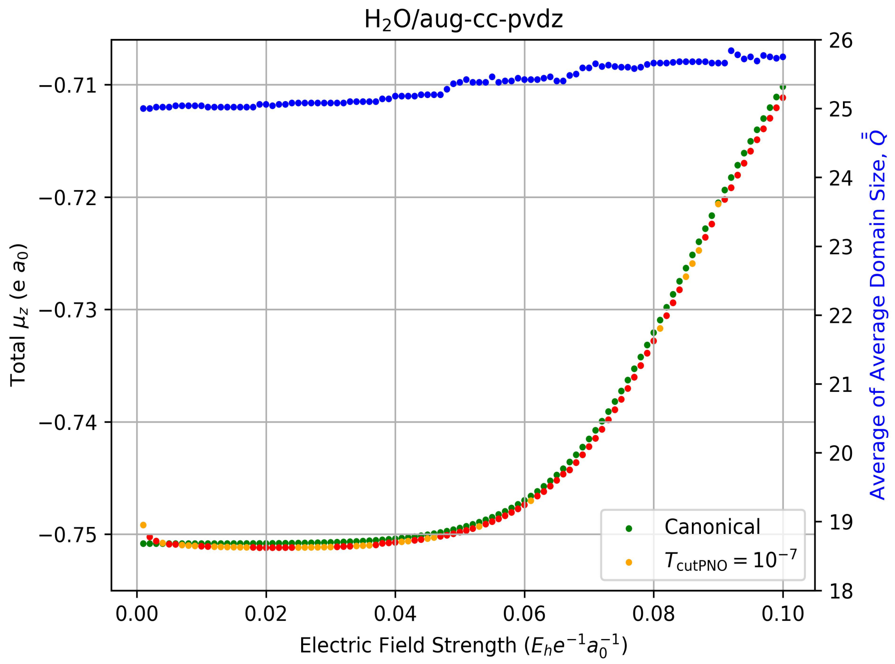

Figure S79: Total contribution to electric dipole moment for water aug-cc-pVDZ with a $T_{\mathrm{cutPNO}} = 10^{-7}$ as a function of external electric field strength.

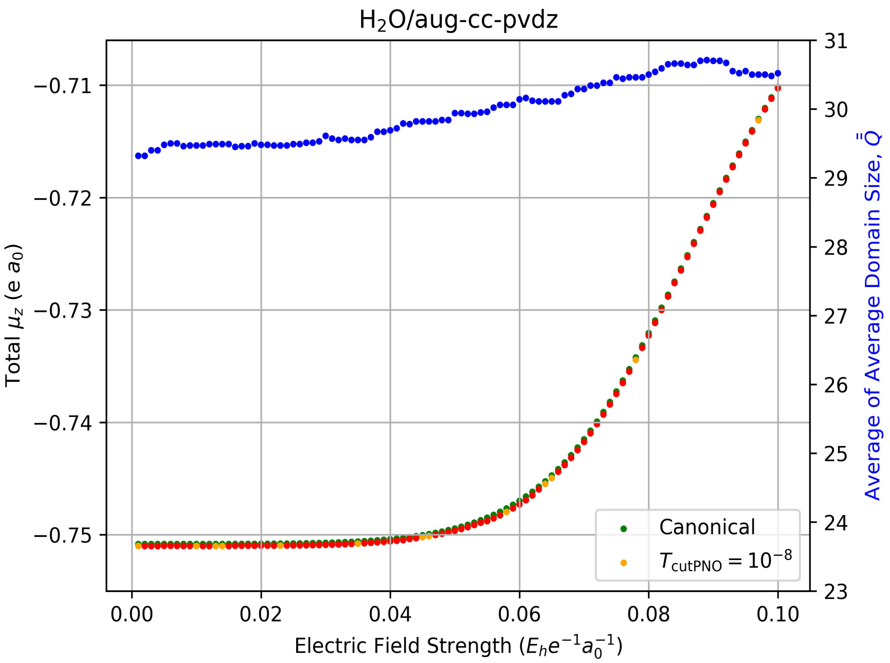

Figure S80: Total contribution to electric dipole moment for water aug-cc-pVDZ with a $T_{\text{cutPNO}} = 10^{-8}$ as a function of external electric field strength.

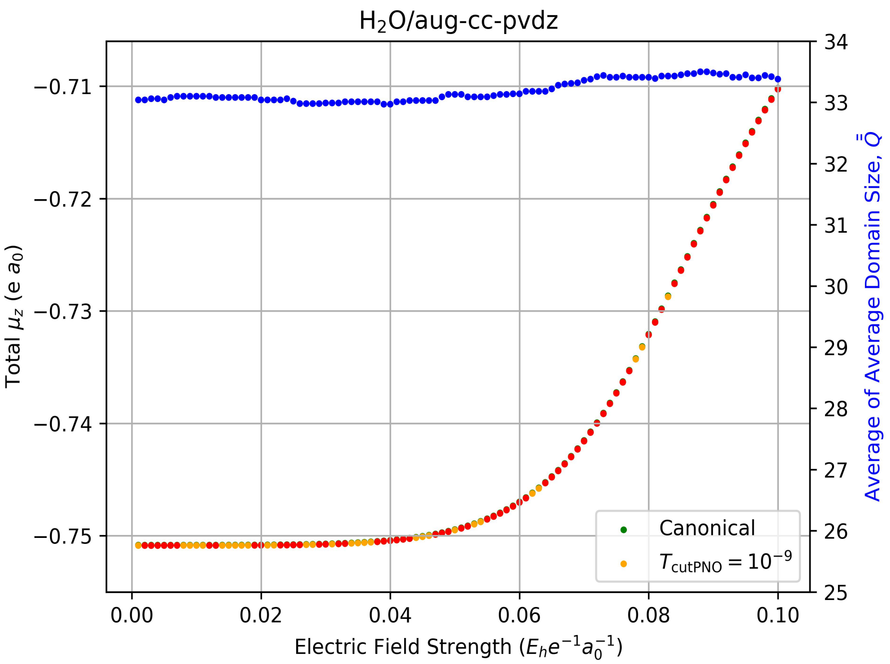

Figure S81: Total contribution to electric dipole moment for water aug-cc-pVDZ with a $T_{\text{cutPNO}} = 10^{-9}$ as a function of external electric field strength.

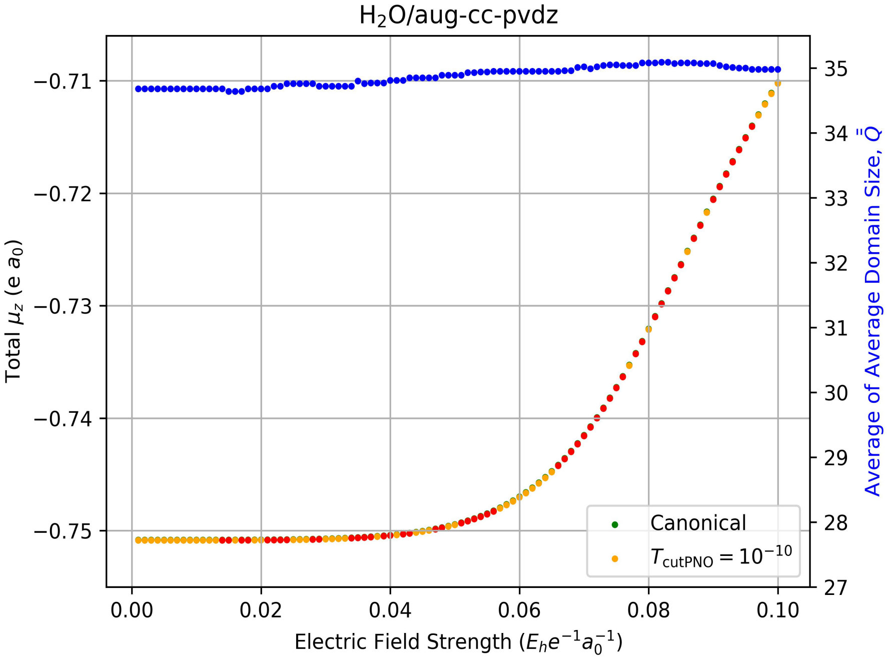

Figure S82: Total contribution to electric dipole moment for water aug-cc-pVDZ with a $T_{\mathrm{cutPNO}} = 10^{-10}$ as a function of external electric field strength.

7.4 Frozen Core Water/cc-pVDZ

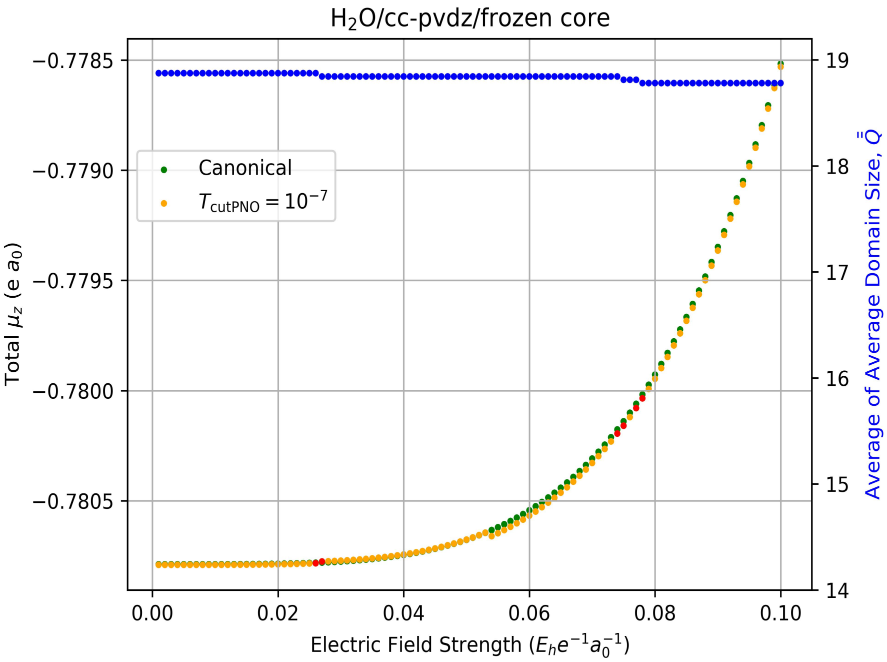

Figure S83: Total contribution to electric dipole moment for frozen core water cc-pVDZ with a $T_{\mathrm{cutPNO}} = 10^{-7}$ as a function of external electric field strength.

7.5 Frozen Core Water/aug-cc-pVDZ

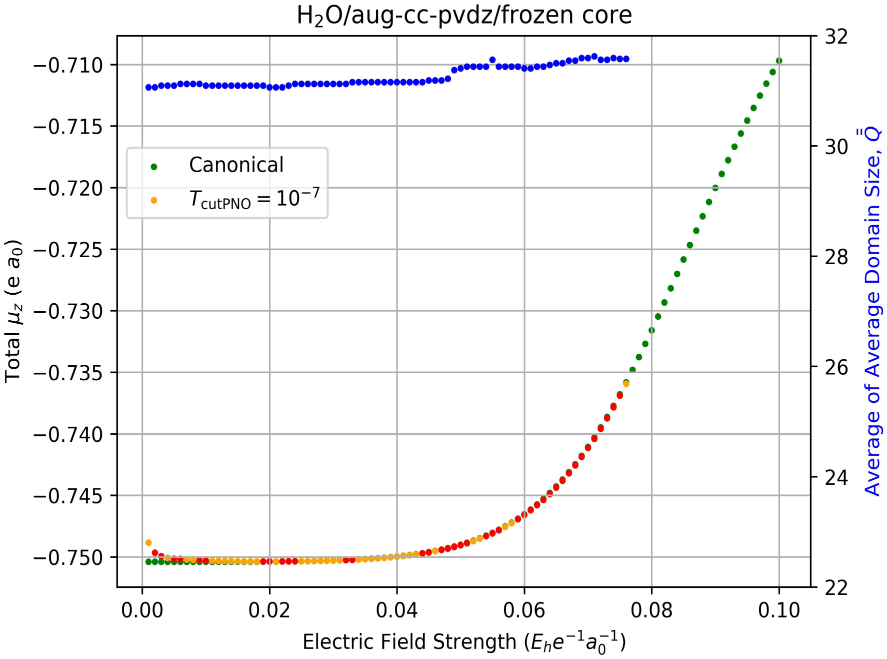

Figure S84: Total contribution to electric dipole moment for frozen core water aug-cc-pVDZ with a $T_{\mathrm{cutPNO}} = 10^{-7}$ as a function of external electric field strength.

7.6 HOF/cc-pVDZ

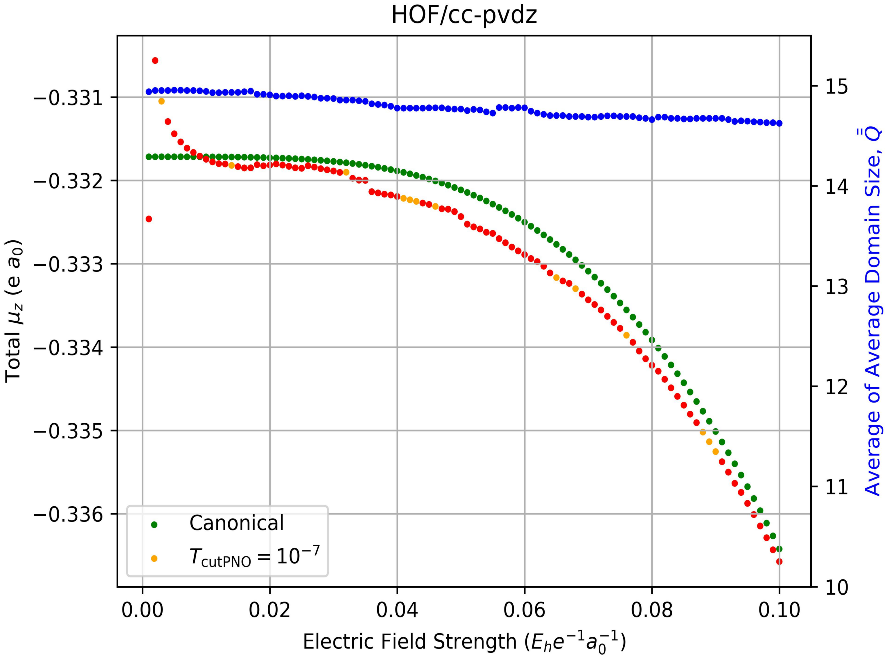

Figure S85: Total contribution to electric dipole moment for HOF cc-pVDZ with a T_{cutPNO} $= 10^{-7}$ as a function of external electric field strength.

7.7 HOF/aug-cc-pVDZ

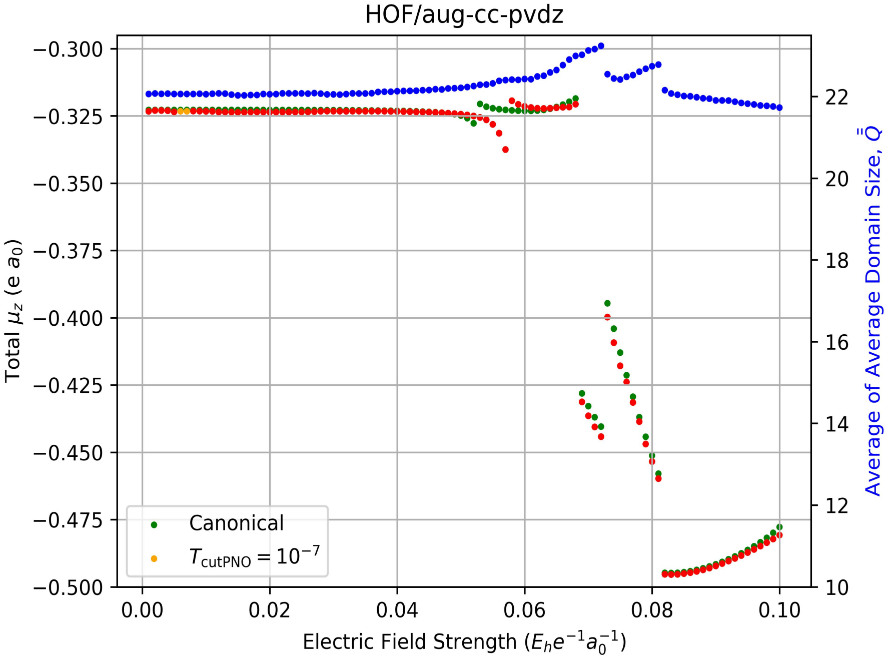

Figure S86: Total contribution to electric dipole moment for HOF aug-cc-pVDZ with a $T_{\text{cutPNO}} = 10^{-7}$ as a function of external electric field strength.

7.8 Fluoroethylene/cc-pVDZ

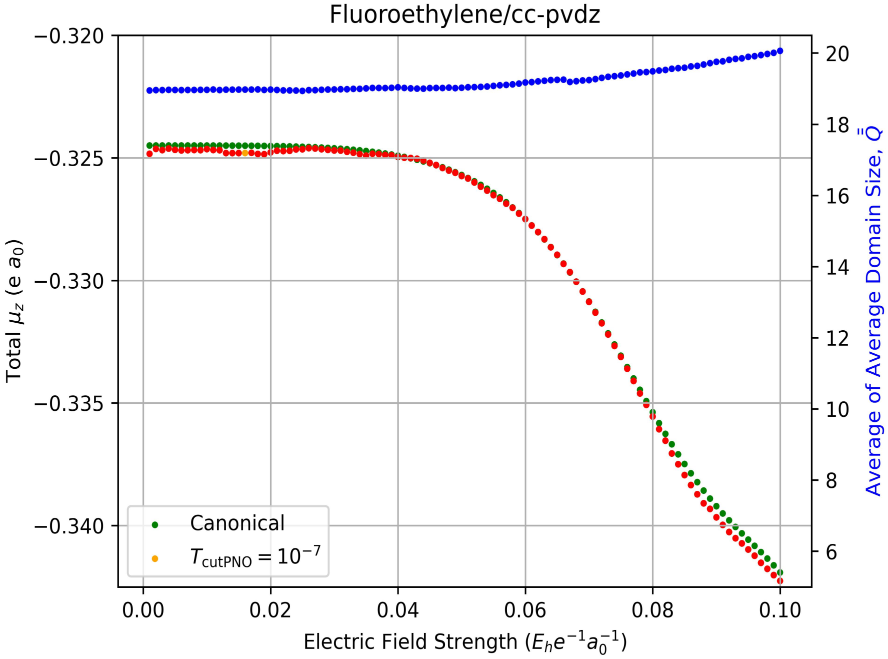

Figure S87: Total contribution to electric dipole moment for fluoroethylene cc-pVDZ with a $T_{\mathrm{cutPNO}} = 10^{-7}$ as a function of external electric field strength.

7.9 Fluoroethylene/aug-cc-pVDZ

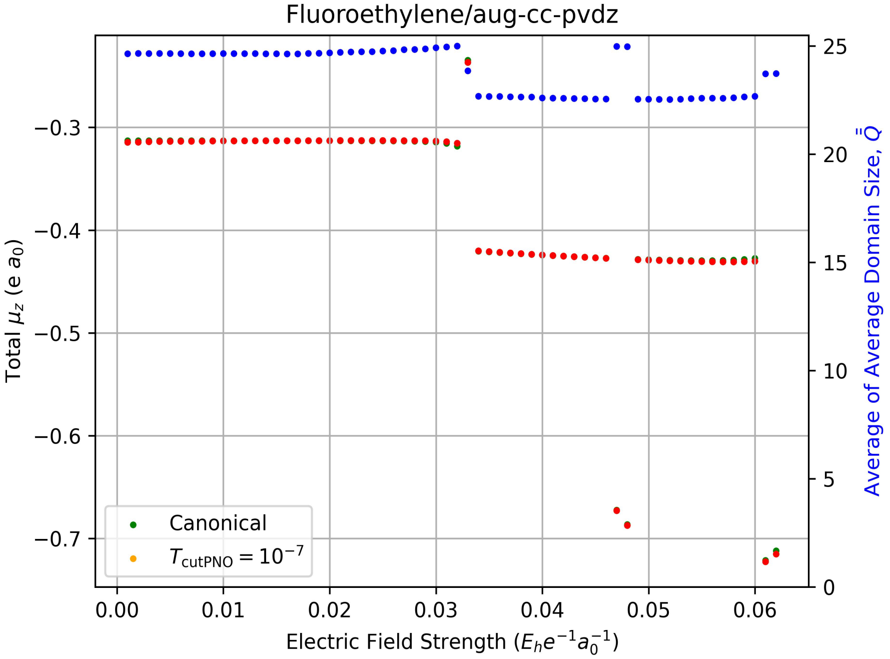

Figure S88: Total contribution to electric dipole moment for fluoroethylene aug-cc-pVDZ with a $T_{\text{cutPNO}} = 10^{-7}$ as a function of external electric field strength.

7.10 Butadiene/cc-pVDZ

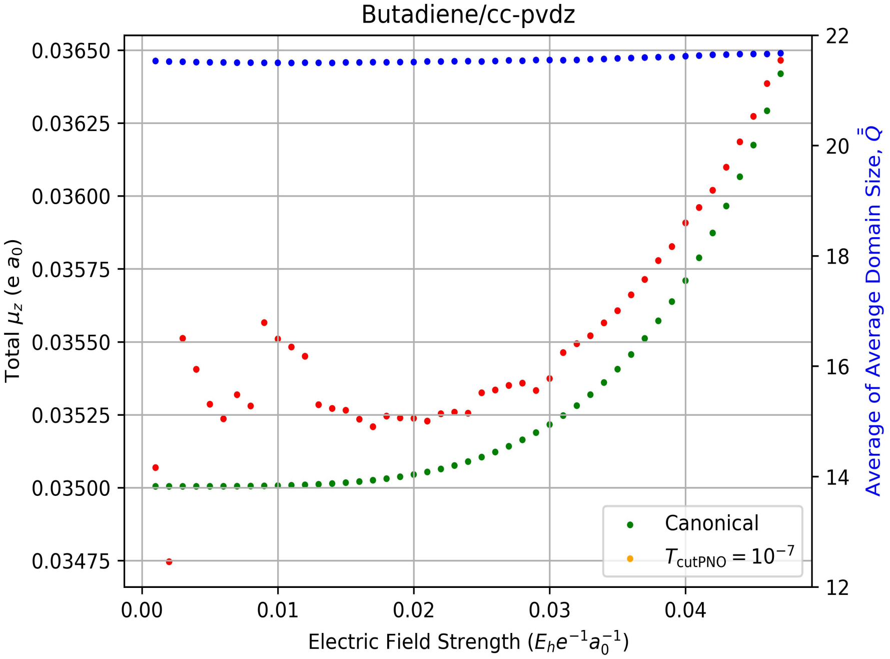

Figure S89: Total contribution to electric dipole moment for butadiene cc-pVDZ with a $T_{\text{cutPNO}} = 10^{-7}$ as a function of external electric field strength.

7.11 Butadiene/aug-cc-pVDZ

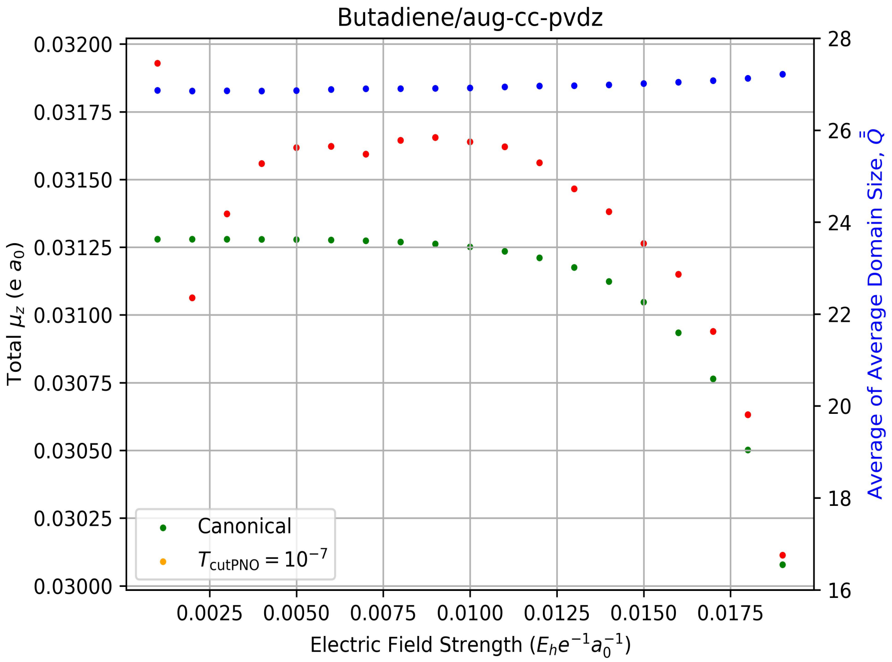

Figure S90: Total contribution to electric dipole moment for butadiene aug-cc-pVDZ with a $T_{\text{cutPNO}} = 10^{-7}$ as a function of external electric field strength.

8 Total Contribution to to Electric Polarizability

8.1 Water/cc-pVDZ

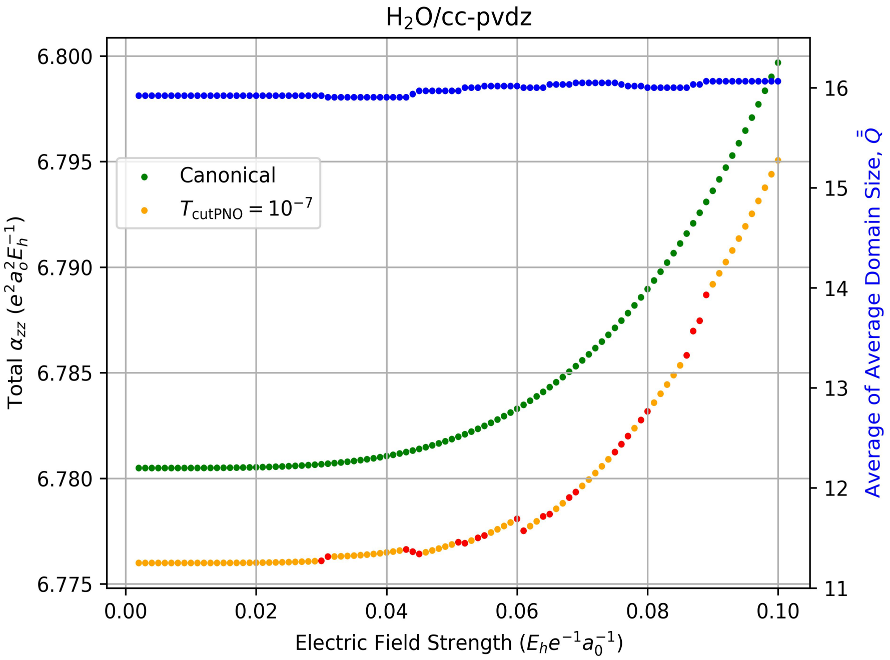

Figure S91: Total contribution to electric polarizability for water cc-pVDZ with a $T_{\text{cutPNO}} = 10^{-7}$ as a function of external electric field strength.

8.2 PNO-Relaxed Water/cc-pVDZ

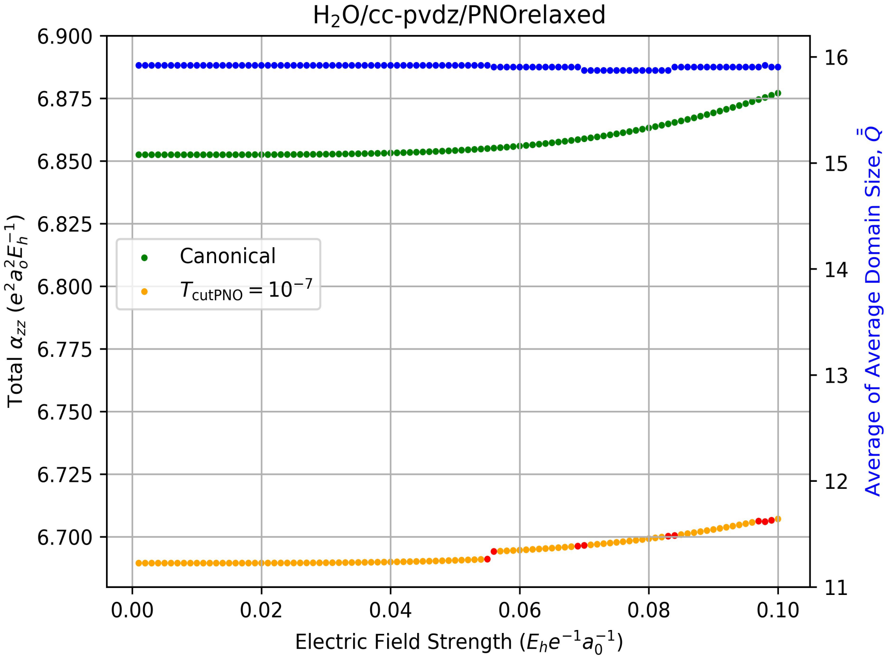

Figure S92: Total contribution to electric polarizability for PNO-relaxed water cc-pVDZ with a $T_{\mathrm{cutPNO}} = 10^{-7}$ as a function of external electric field strength.

8.3 Water/aug-cc-pVDZ

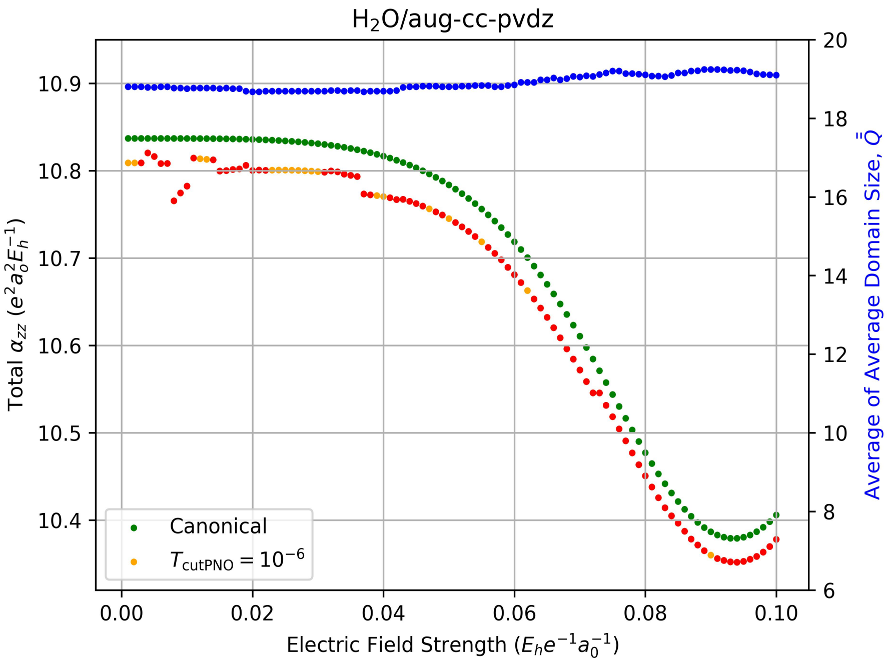

Figure S93: Total contribution to electric polarizability for water aug-cc-pVDZ with a T_{cutPNO} $= 10^{-6}$ as a function of external electric field strength.

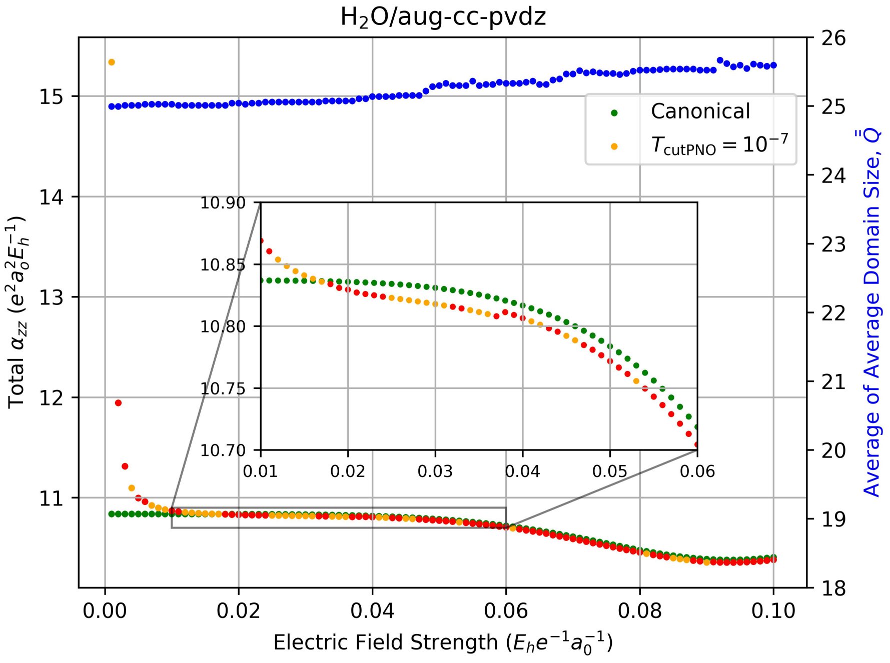

Figure S94: Total contribution to electric polarizability for water aug-cc-pVDZ with a T_{cutPNO} $= 10^{-7}$ as a function of external electric field strength.

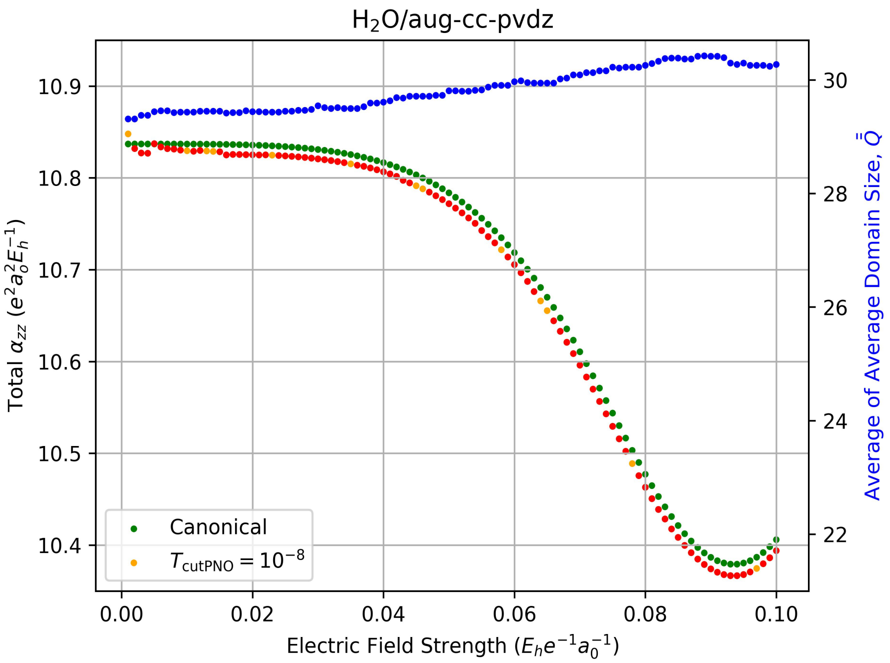

Figure S95: Total contribution to electric polarizability for water aug-cc-pVDZ with a T_{cutPNO} $= 10^{-8}$ as a function of external electric field strength.

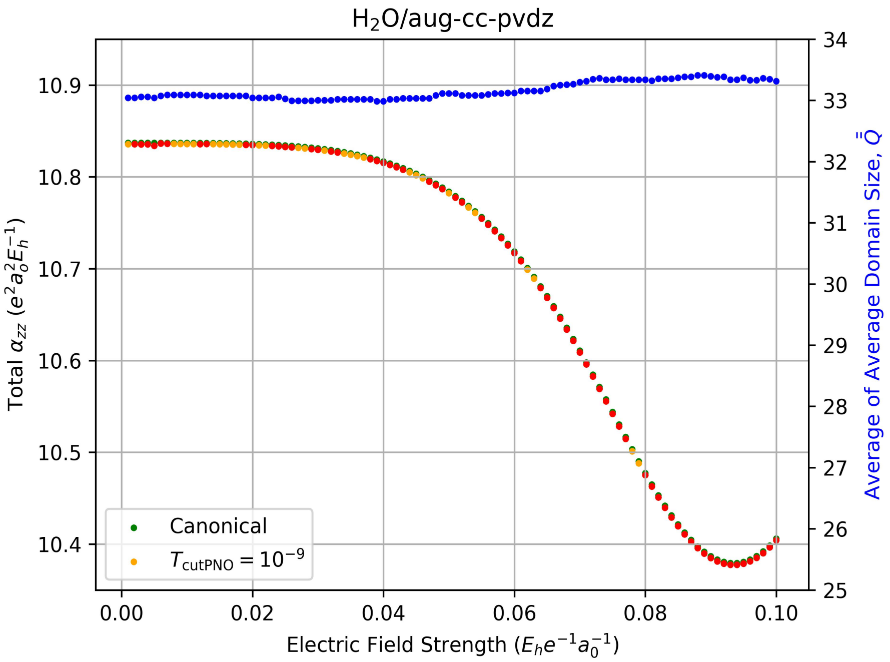

Figure S96: Total contribution to electric polarizability for water aug-cc-pVDZ with a T_{cutPNO} $= 10^{-9}$ as a function of external electric field strength.

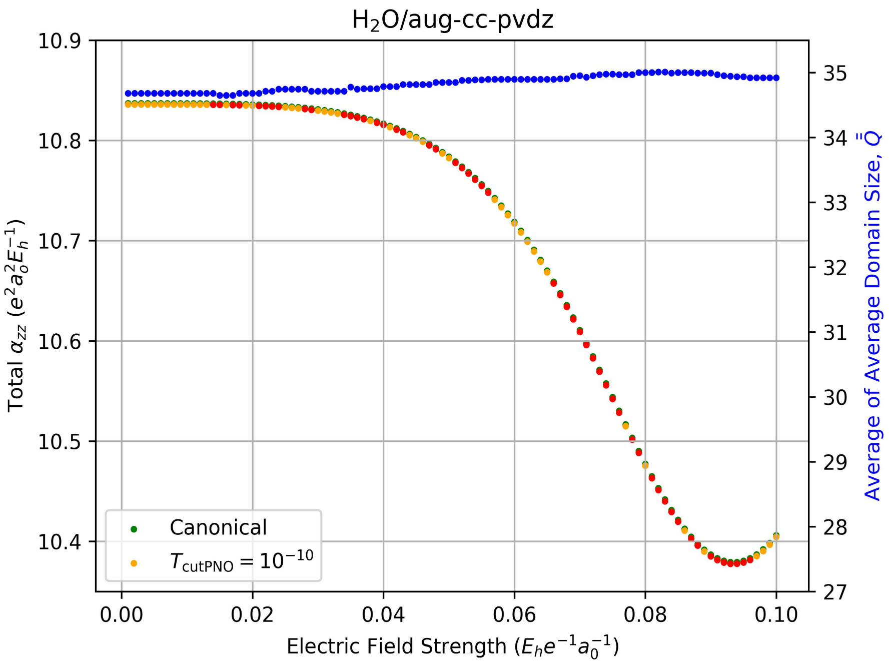

Figure S97: Total contribution to electric polarizability for water aug-cc-pVDZ with a T_{cutPNO} $= 10^{-10}$ as a function of external electric field strength.

8.4 Frozen Core Water/cc-pVDZ

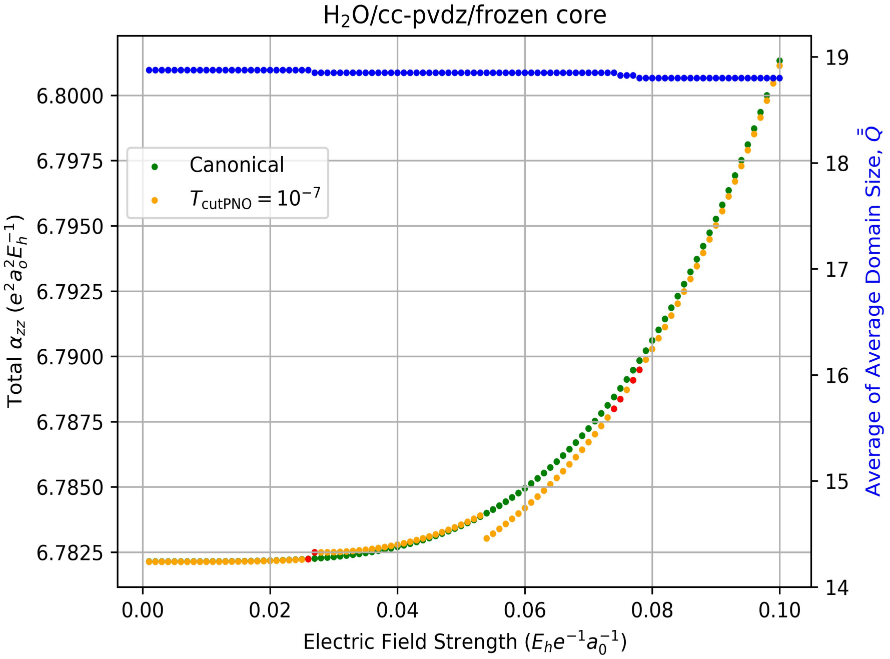

Figure S98: Total contribution to electric polarizability for frozen core water cc-pVDZ with a $T_{\mathrm{cutPNO}} = 10^{-7}$ as a function of external electric field strength.

8.5 Frozen Core Water/aug-cc-pVDZ

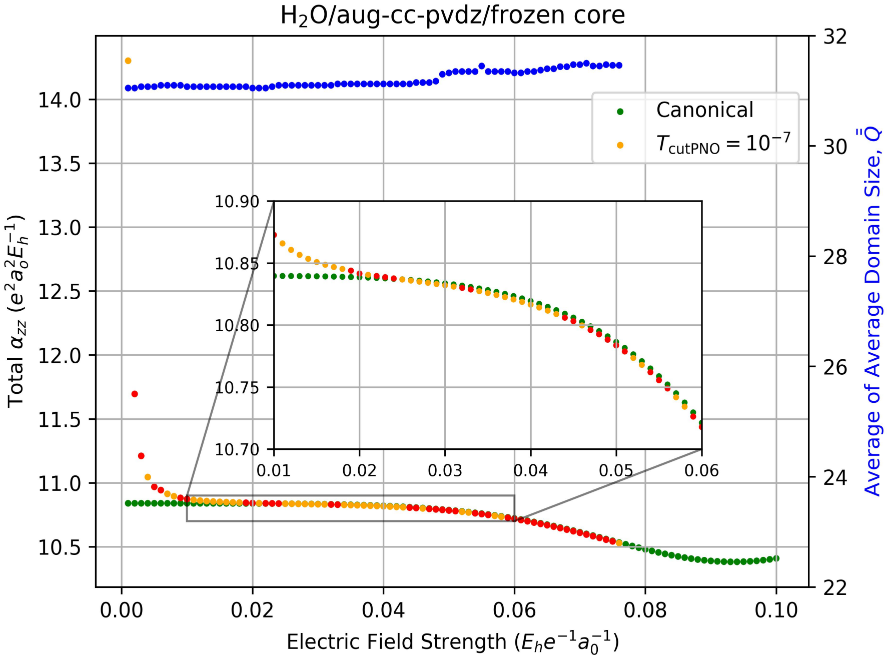

Figure S99: Total contribution to electric polarizability for frozen core water aug-cc-pVDZ with a $T_{\mathrm{cutPNO}} = 10^{-7}$ as a function of external electric field strength.

8.6 HOF/cc-pVDZ

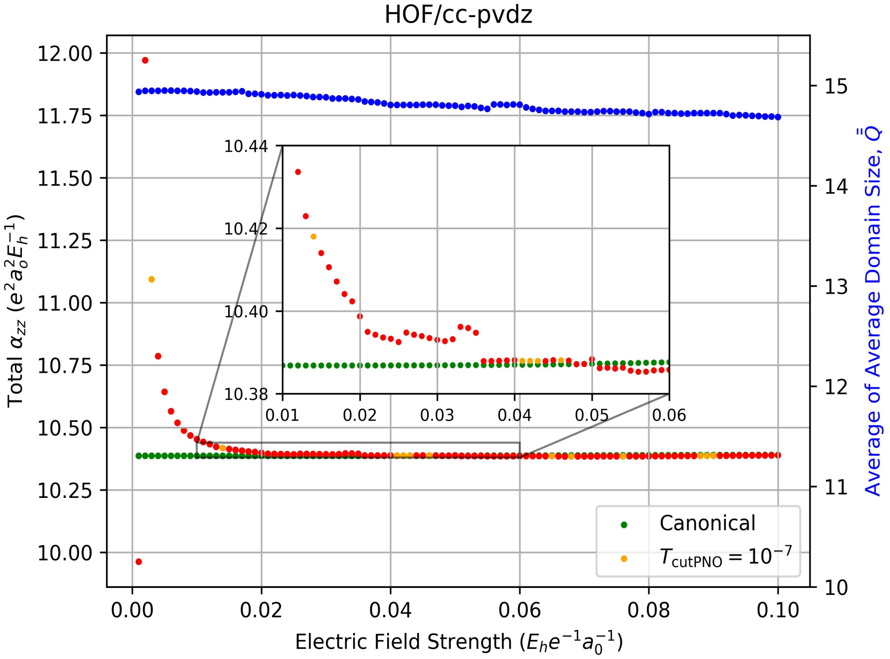

Figure S100: Total contribution to electric polarizability for HOF cc-pVDZ with a T_{cutPNO} $= 10^{-7}$ as a function of external electric field strength.

8.7 HOF/aug-cc-pVDZ

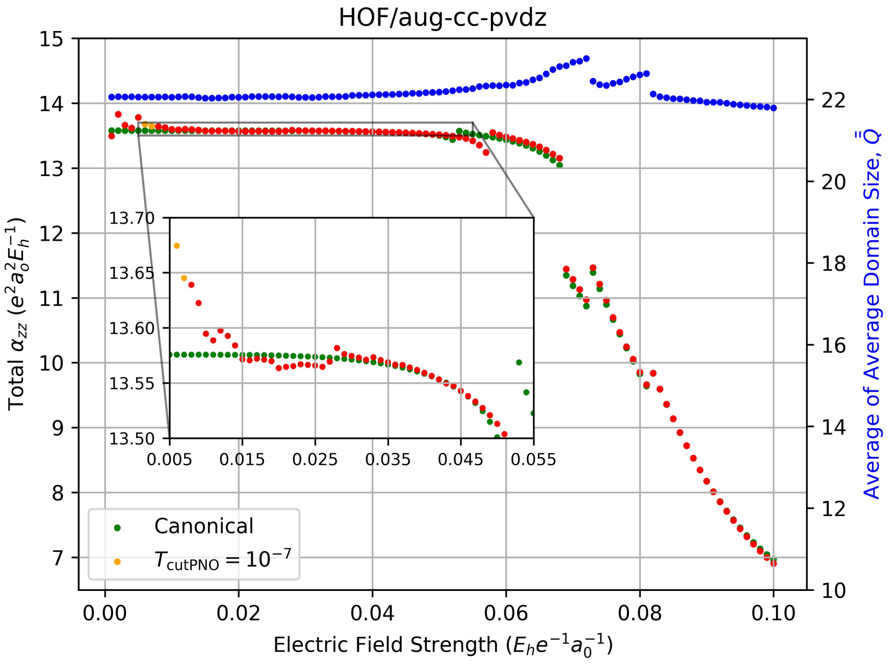

Figure S101: Total contribution to electric polarizability for HOF aug-cc-pVDZ with a $T_{\text{cutPNO}} = 10^{-7}$ as a function of external electric field strength.

8.8 Fluoroethylene/cc-pVDZ

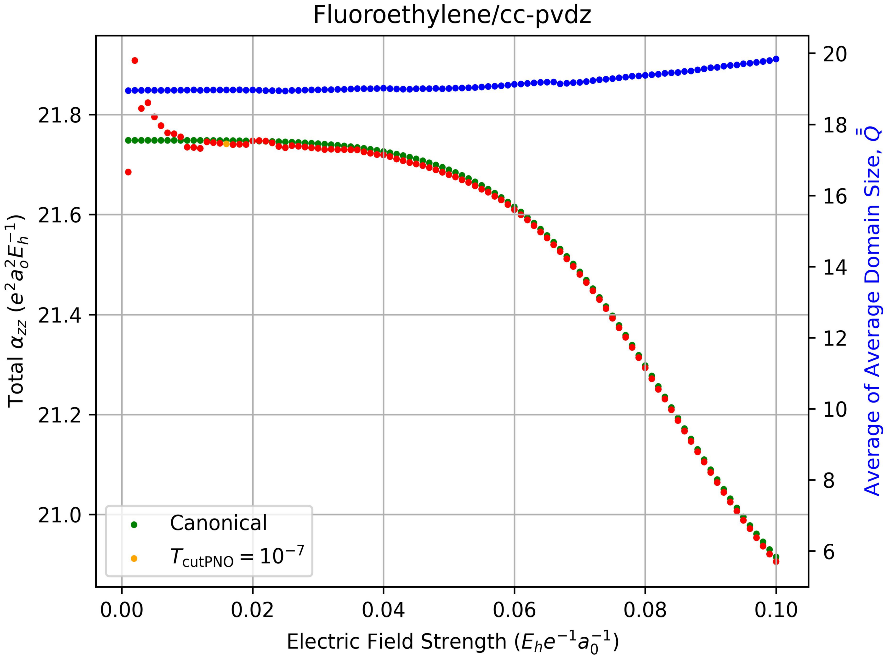

Figure S102: Total contribution to electric polarizability for fluoroethylene cc-pVDZ with a $T_{\text{cutPNO}} = 10^{-7}$ as a function of external electric field strength.

8.9 Fluoroethylene/aug-cc-pVDZ

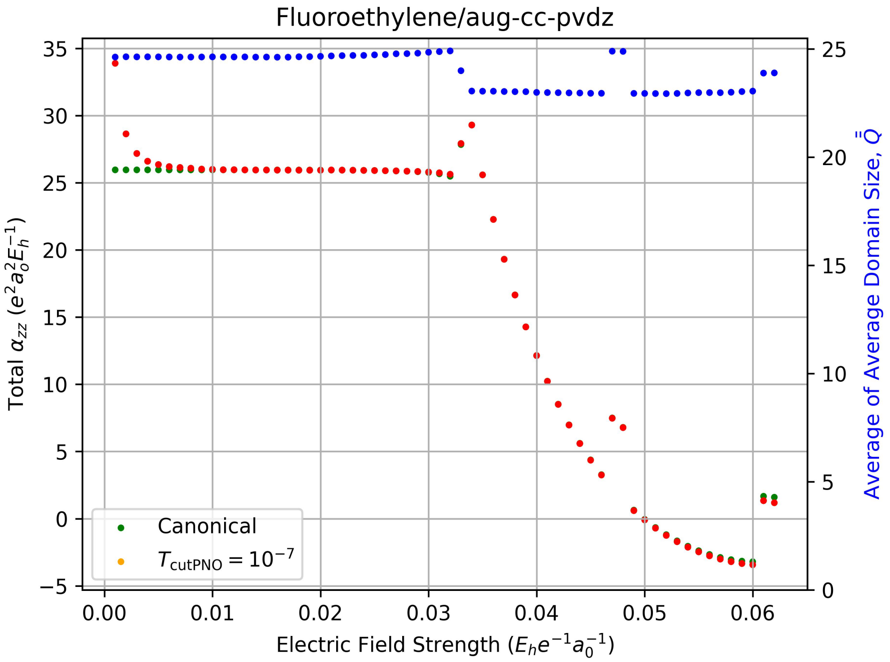

Figure S103: Total contribution to electric polarizability for fluoroethylene aug-cc-pVDZ with a $T_{\mathrm{cutPNO}} = 10^{-7}$ as a function of external electric field strength.

8.10 Butadiene/cc-pVDZ

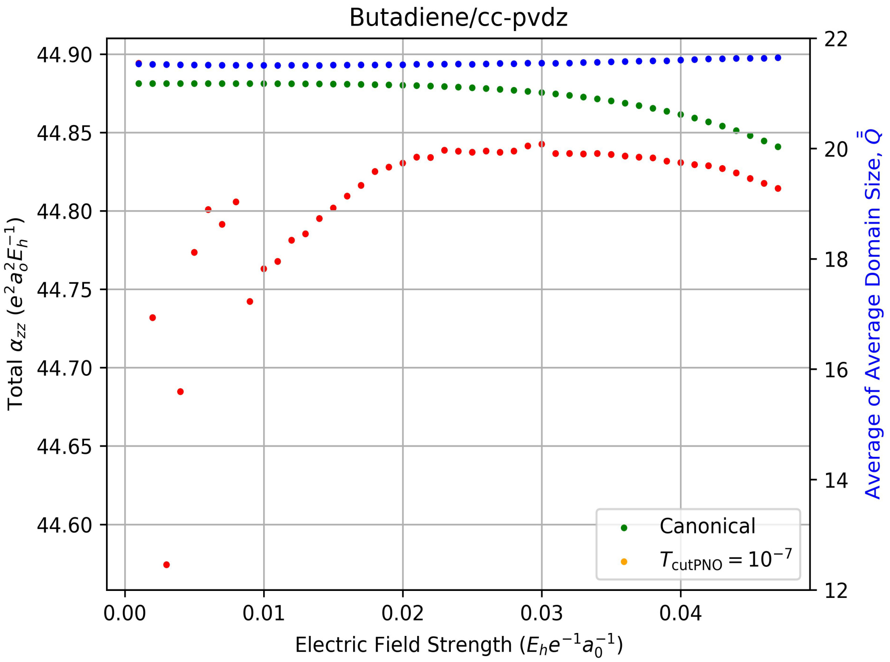

Figure S104: Total contribution to electric polarizability for butadiene cc-pVDZ with a $T_{\text{cutPNO}} = 10^{-7}$ as a function of external electric field strength.

8.11 Butadiene/aug-cc-pVDZ

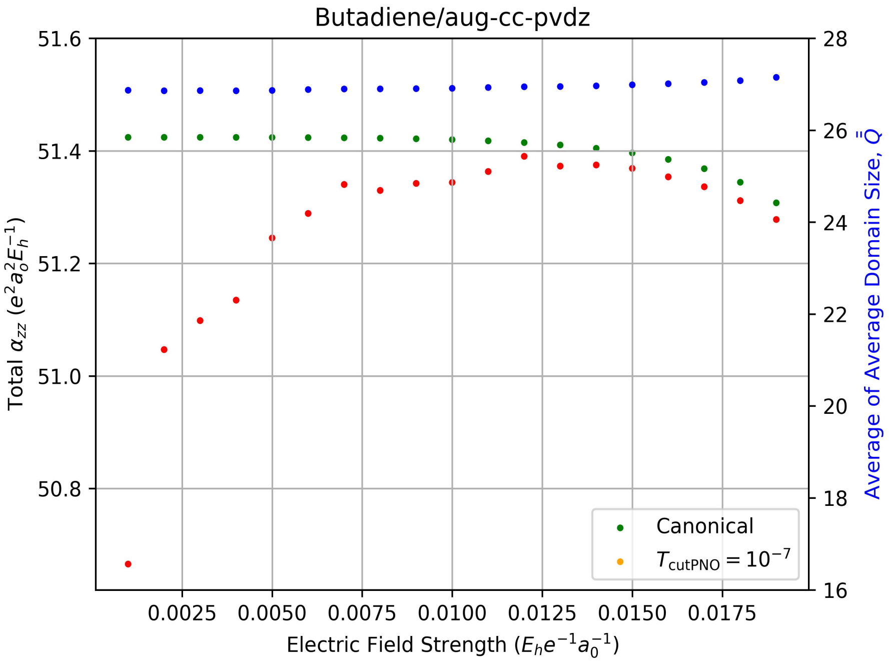

Figure S105: Total contribution to electric polarizability for butadiene aug-cc-pVDZ with a $T_{\text{cutPNO}} = 10^{-7}$ as a function of external electric field strength.

9 Total Contribution to to Electric Hyperpolarizability

9.1 Water/cc-pVDZ

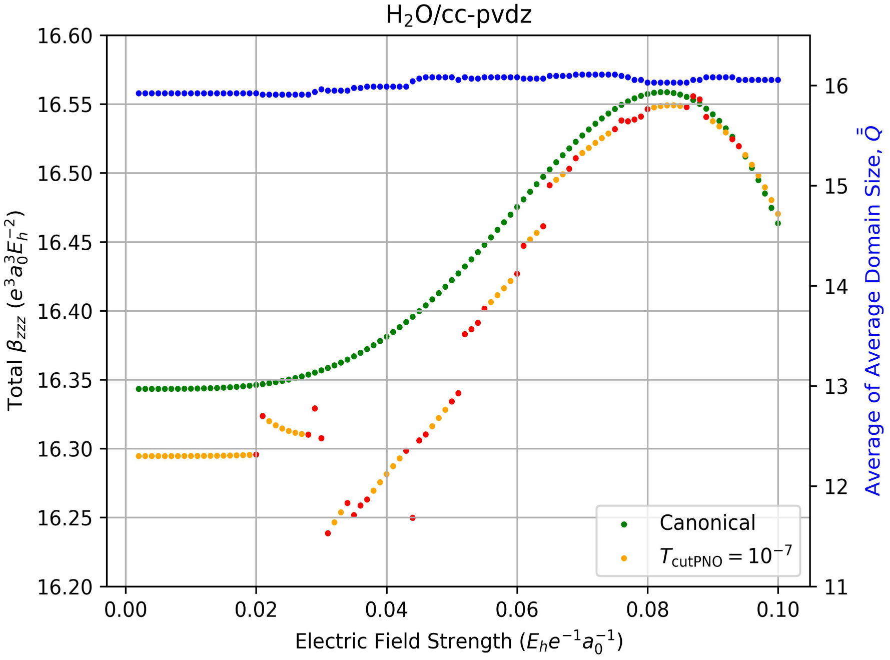

Figure S106: Total contribution to electric hyperpolarizability for water cc-pVDZ with a $T_{\mathrm{cutPNO}} = 10^{-7}$ as a function of external electric field strength.

9.2 PNO-Relaxed Water/cc-pVDZ

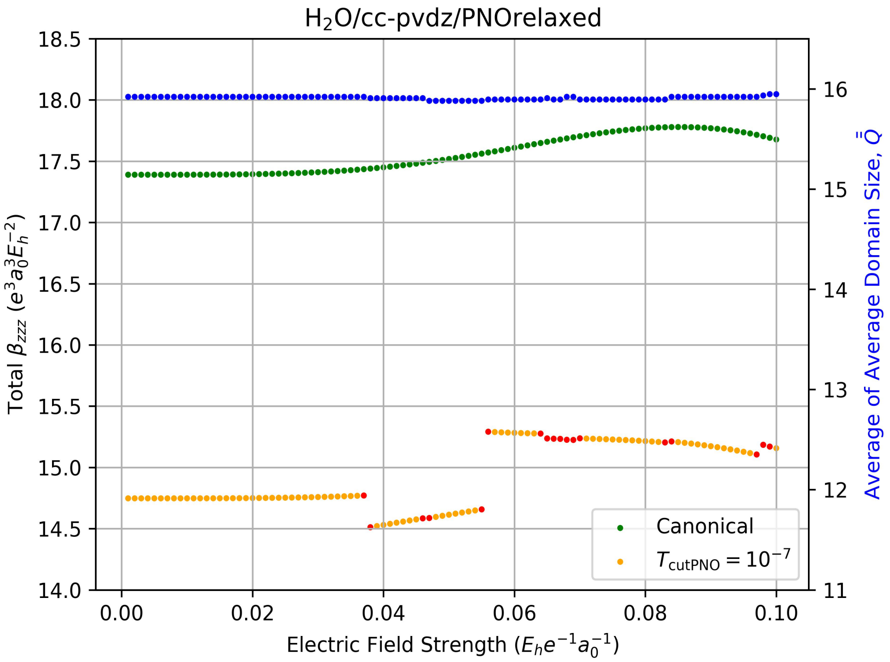

Figure S107: Total contribution to electric hyperpolarizability for PNO-relaxed water cc-pVDZ with a $T_{\mathrm{cutPNO}} = 10^{-7}$ as a function of external electric field strength.

9.3 Water/aug-cc-pVDZ

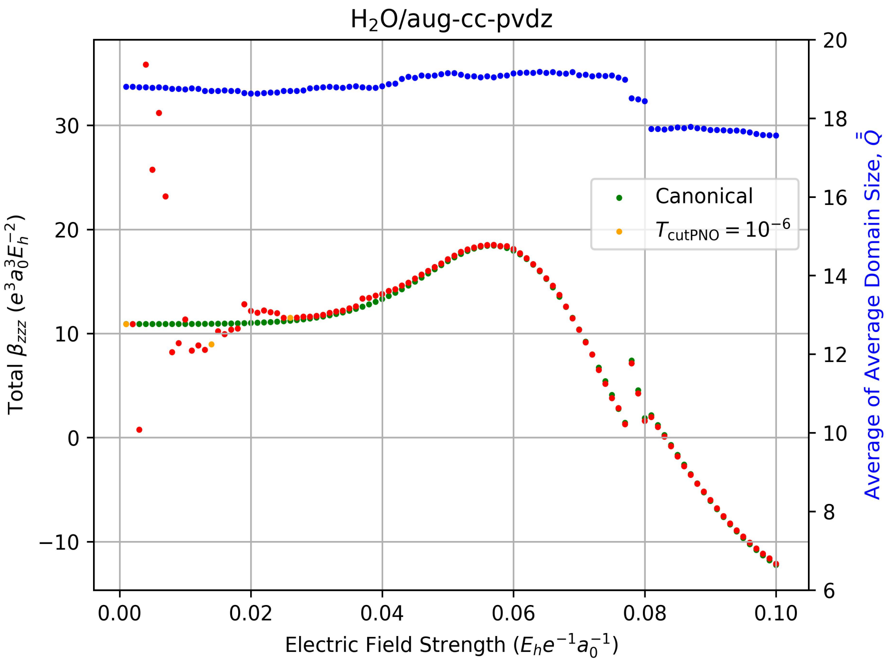

Figure S108: Total contribution to electric hyperpolarizability for water aug-cc-pVDZ with a $T_{\mathrm{cutPNO}} = 10^{-6}$ as a function of external electric field strength.

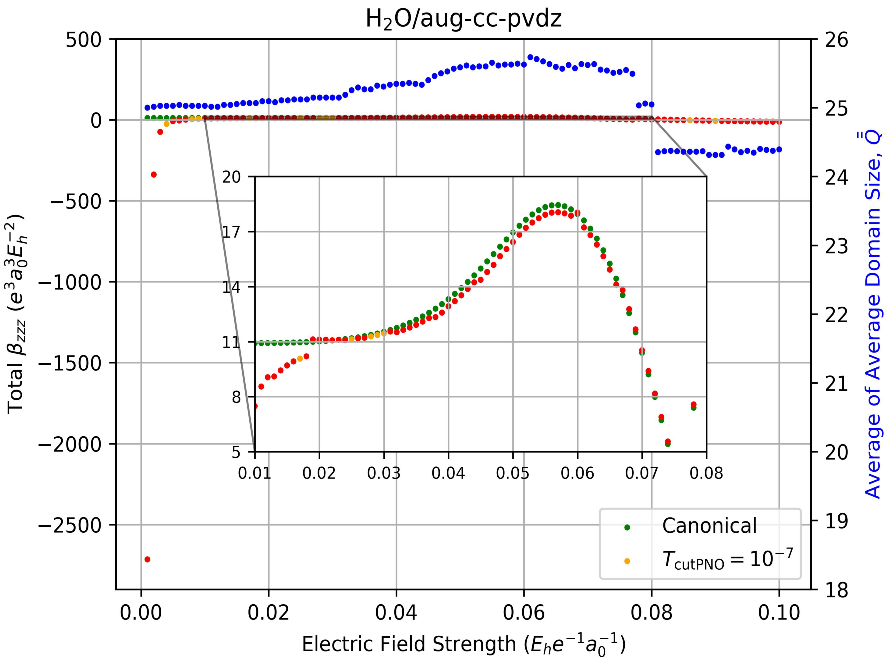

Figure S109: Total contribution to electric hyperpolarizability for water aug-cc-pVDZ with a $T_{\mathrm{cutPNO}} = 10^{-7}$ as a function of external electric field strength.

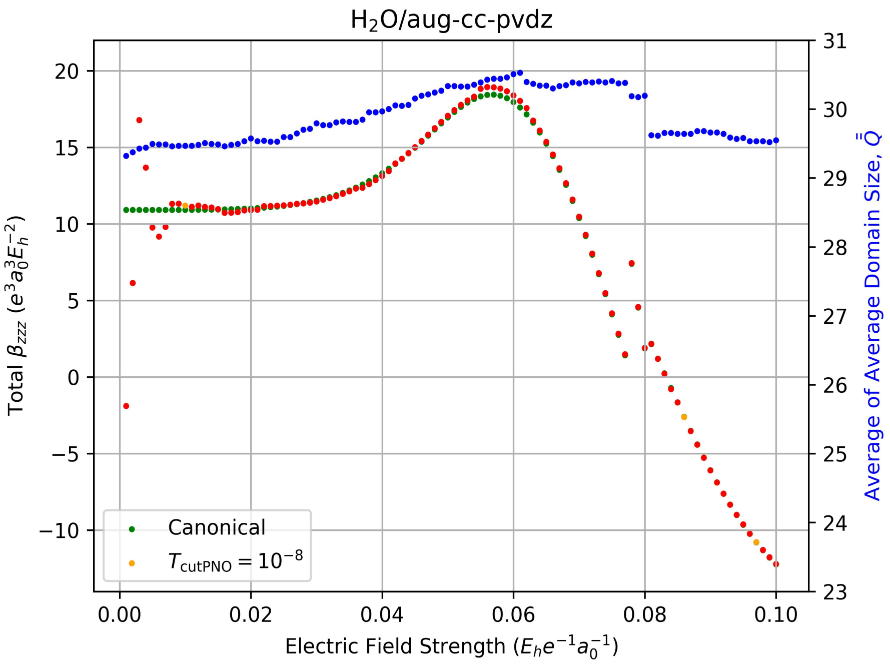

Figure S110: Total contribution to electric hyperpolarizability for water aug-cc-pVDZ with a $T_{\text{cutPNO}} = 10^{-8}$ as a function of external electric field strength.

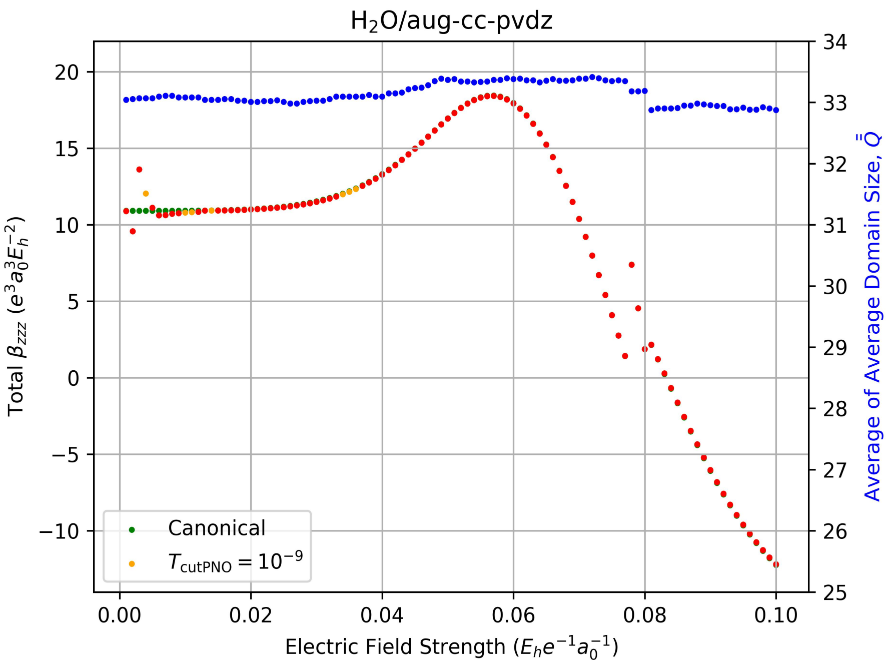

Figure S111: Total contribution to electric hyperpolarizability for water aug-cc-pVDZ with a $T_{\text{cutPNO}} = 10^{-9}$ as a function of external electric field strength.

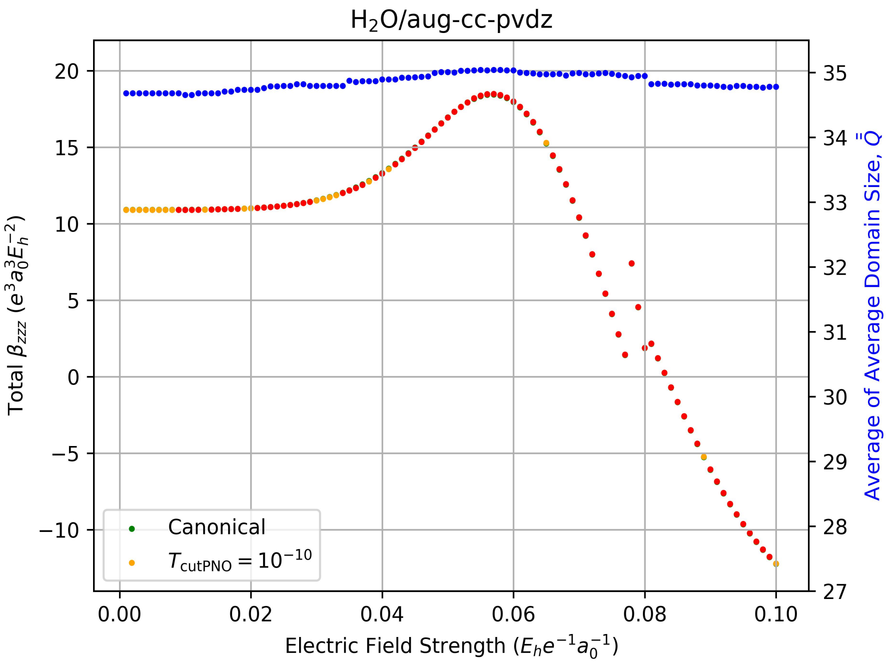

Figure S112: Total contribution to electric hyperpolarizability for water aug-cc-pVDZ with a $T_{\text{cutPNO}} = 10^{-10}$ as a function of external electric field strength.

9.4 Frozen Core Water/cc-pVDZ

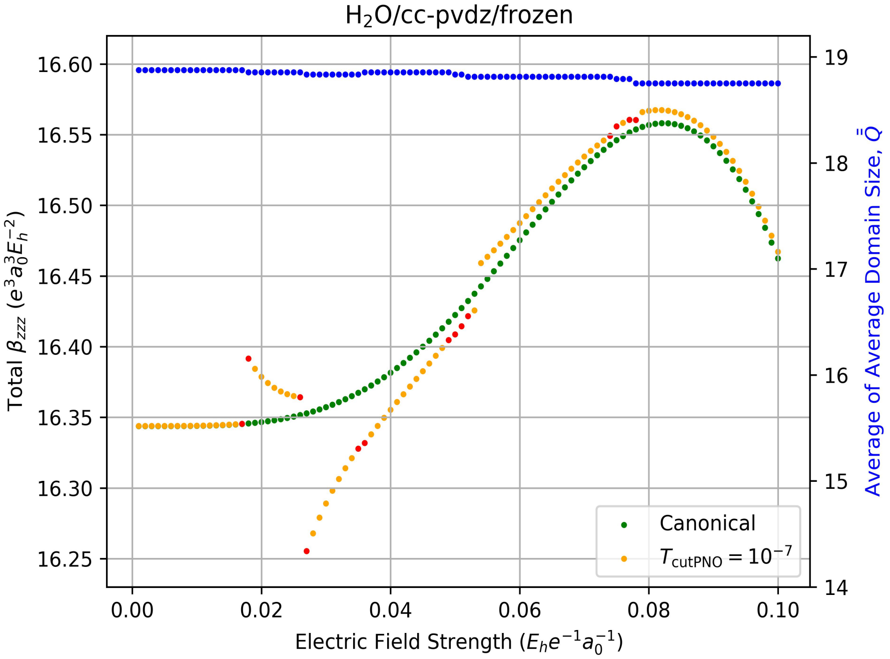

Figure S113: Total contribution to electric hyperpolarizability for frozen core water cc-pVDZ with a $T_{\mathrm{cutPNO}} = 10^{-7}$ as a function of external electric field strength.

9.5 Frozen Core Water/aug-cc-pVDZ

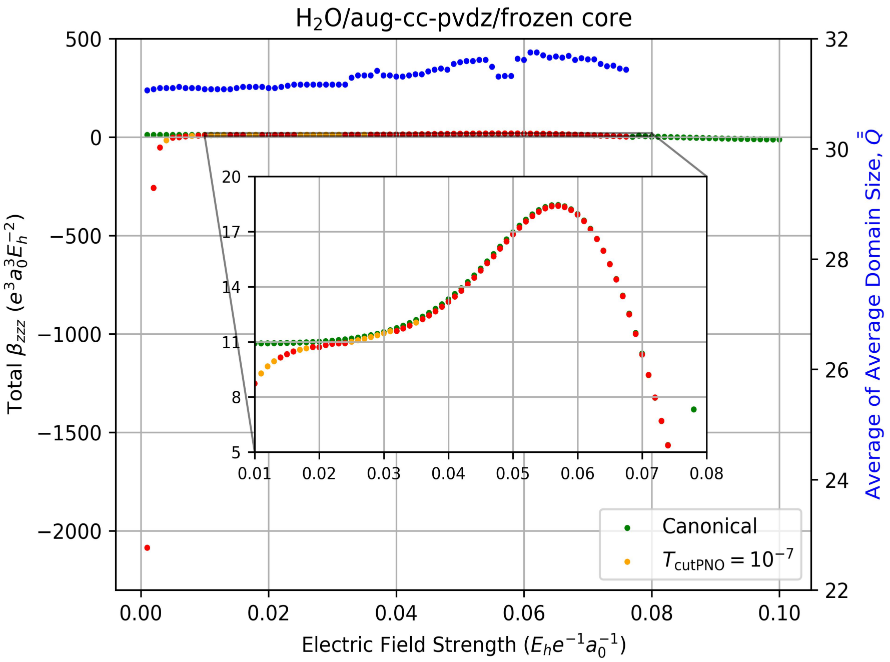

Figure S114: Total contribution to electric hyperpolarizability for frozen core water aug-cc-pVDZ with a $T_{\text{cutPNO}} = 10^{-7}$ as a function of external electric field strength.

9.6 HOF/cc-pVDZ

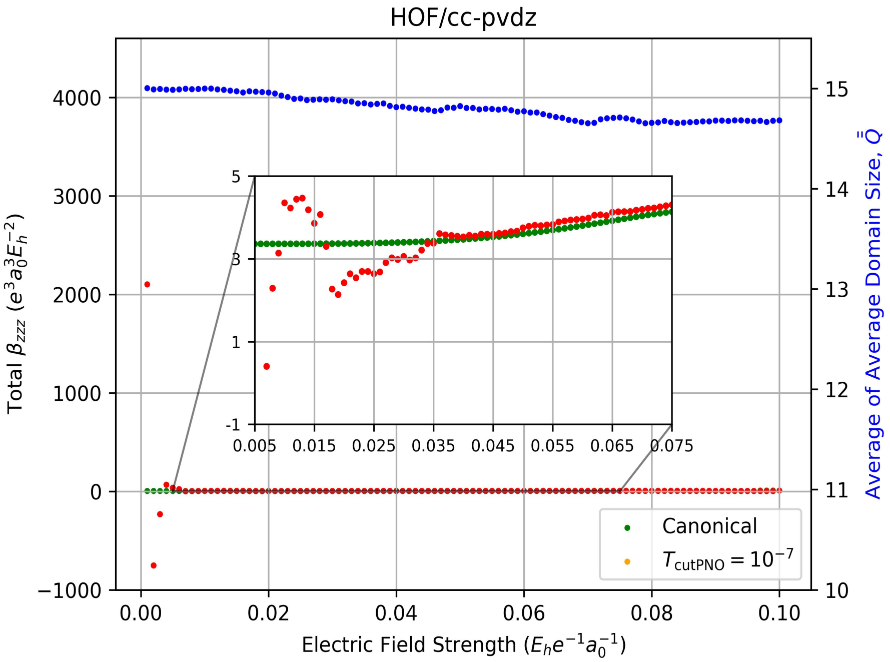

Figure S115: Total contribution to electric hyperpolarizability for HOF cc-pVDZ with a $T_{\text{cutPNO}} = 10^{-7}$ as a function of external electric field strength.

9.7 HOF/aug-cc-pVDZ

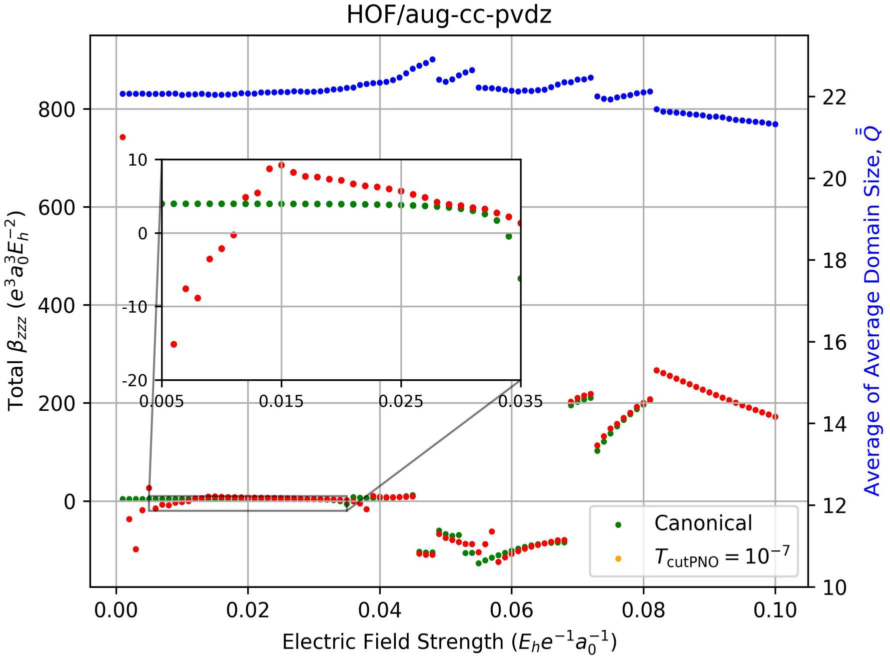

Figure S116: Total contribution to electric hyperpolarizability for HOF aug-cc-pVDZ with a $T_{\text{cutPNO}} = 10^{-7}$ as a function of external electric field strength.

9.8 Fluoroethylene/cc-pVDZ

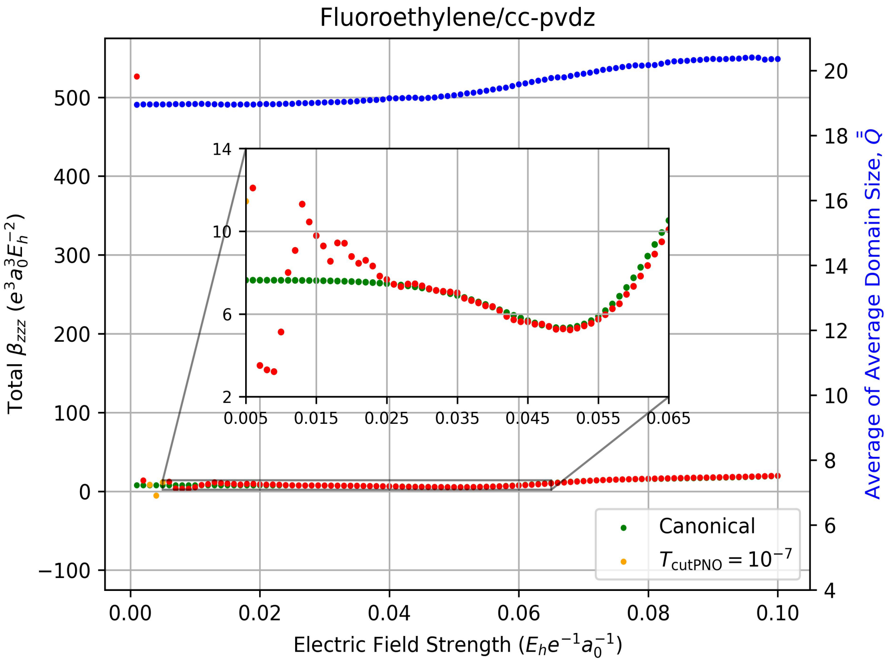

Figure S117: Total contribution to electric hyperpolarizability for fluoroethylene cc-pVDZ with a $T_{\mathrm{cutPNO}} = 10^{-7}$ as a function of external electric field strength.

9.9 Fluoroethylene/aug-cc-pVDZ

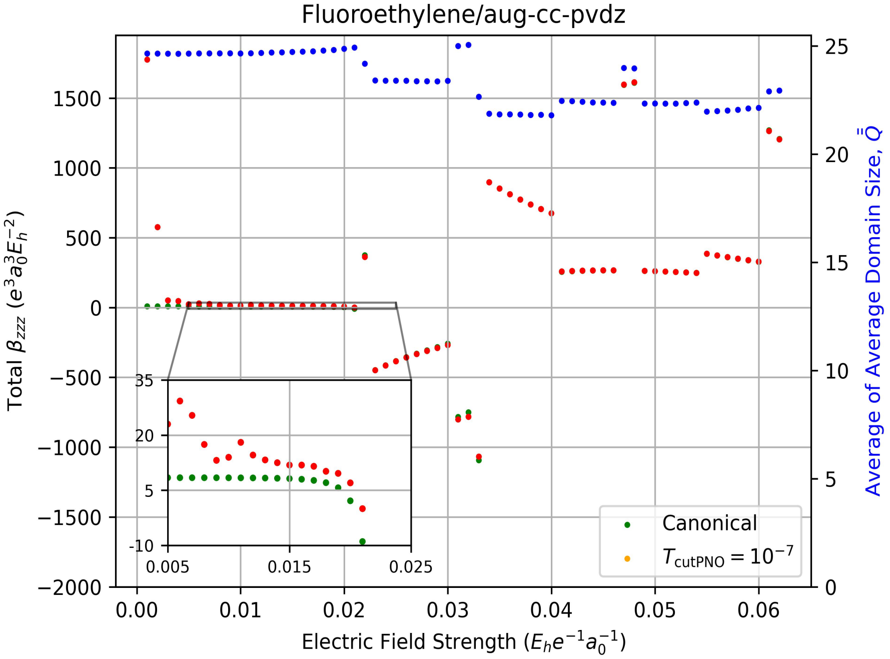

Figure S118: Total contribution to electric hyperpolarizability for fluoroethylene aug-cc-pVDZ with a $T_{\mathrm{cutPNO}} = 10^{-7}$ as a function of external electric field strength.

9.10 Butadiene/cc-pVDZ

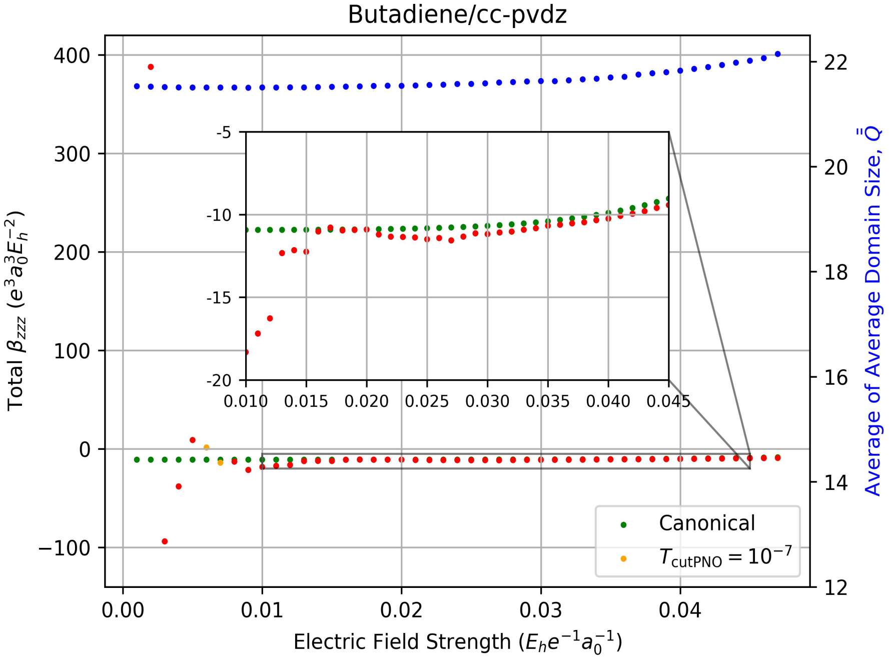

Figure S119: Total contribution to electric hyperpolarizability for butadiene cc-pVDZ with a $T_{\mathrm{cutPNO}} = 10^{-7}$ as a function of external electric field strength.

9.11 Butadiene/aug-cc-pVDZ

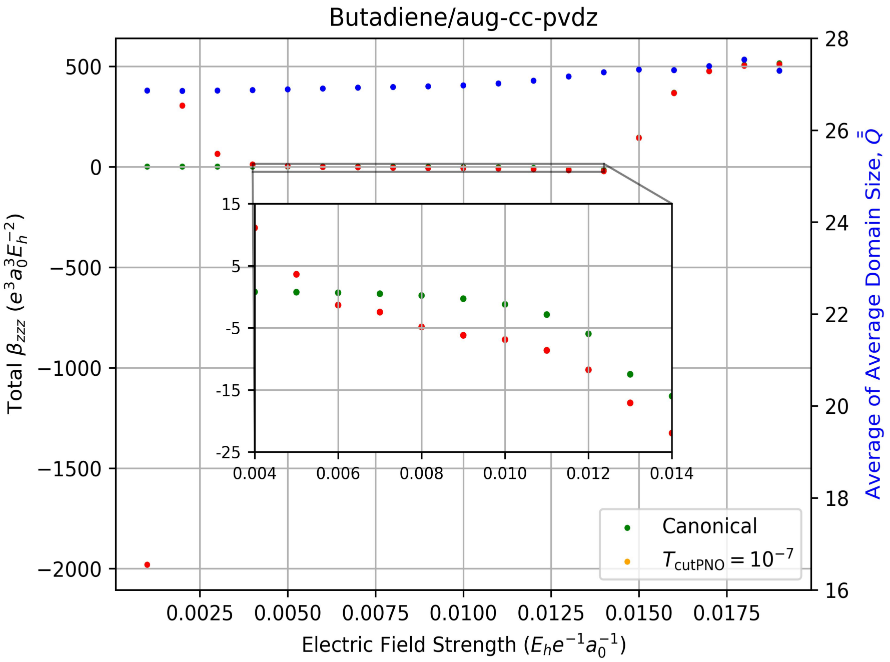

Figure S120: Total contribution to electric hyperpolarizability for butadiene aug-cc-pVDZ with a $T_{\text{cutPNO}} = 10^{-7}$ as a function of external electric field strength.
